# Systems pharmacology reveals type I interferon and myeloid-like B cell reprogramming as candidate druggable axes in antiphospholipid syndrome

**DOI:** 10.1371/journal.pone.0349155

**Published:** 2026-08-26

**Authors:** Bin Sun, Yiqiao Lu, Wei Liu, Chengze Wang

**Affiliations:** 1 Wuxi No. 2 People's Hospital, Jiangnan University Medical Center, Wuxi, China; 2 Women's Hospital School of Medicine Zhejiang University, Zhejiang University, Hangzhou, China; 3 Zhejiang University School of Medicine, Clinical Research Center for Oral Diseases of Zhejiang Province, Key Laboratory of Oral Biomedical Research of Zhejiang Province, Cancer Center of Zhejiang University, Hangzhou, China; Versiti Blood Research Institute, UNITED STATES OF AMERICA

## Abstract

Antiphospholipid syndrome (APS) lacks disease-modifying targeted therapies, and its molecular heterogeneity remains poorly characterized. We employed an integrative systems pharmacology approach combining weighted gene co-expression network analysis (WGCNA), single-cell RNA sequencing, Connectivity Map (CMap) screening, and molecular docking to prioritize candidate therapeutic targets in APS. WGCNA of purified-neutrophil bulk RNA-seq (n = 18), with module preservation confirmed in whole blood (n = 88), identified two disease-associated modules: ME10 (176 genes, r = 0.77, interferon-I signaling) and ME2 (3409 genes, r = 0.79, degranulation/innate activation). Single-cell analysis of 26,936 B cells revealed transitional B cells with elevated ME2 scores and aberrant SPI1 expression, suggesting myeloid-like transcriptional reprogramming. CMap analysis ranked chloroquine — a 4-aminoquinoline antimalarial closely related to hydroxychloroquine, which is recommended as adjunctive therapy in APS — among top ME2 candidates (NCS = −2.07), supporting the biological relevance of the screen. DrugBank mapping identified 14 FDA-approved drugs targeting module genes, and a 3-gene machine learning signature (CORO1A, ANKRD22, IFITM1) achieved cross-tissue validation AUC of 0.802. External datasets supported ME2 pathway modulation by NAPc2 intervention and cross-tissue module conservation in platelets. Patient-level ME10 x ME2 stratification revealed four molecular subtypes with distinct pathway activation profiles. This framework prioritizes candidate targets across both IFN-I and degranulation pathways, generating hypotheses for pathway-guided therapeutic development that require experimental and clinical validation.

## Introduction

Antiphospholipid syndrome (APS) is a systemic autoimmune disorder characterized by arterial and venous thrombosis, recurrent pregnancy loss, and the persistent presence of antiphospholipid antibodies (aPL) [1–3]. Antiphospholipid antibodies are detectable in approximately 1–5% of the general population, whereas APS itself is considerably rarer, with an estimated prevalence of approximately 40–50 per 100,000. As a major cause of acquired thrombophilia, APS is managed largely with anticoagulation, complemented by hydroxychloroquine and low-dose aspirin in selected patients; however, these strategies do not directly target the underlying pathogenic mechanisms [4,5]. The heterogeneity in clinical manifestations and treatment responses among APS patients suggests distinct molecular endotypes that have yet to be systematically characterized [6].

Recent advances in systems biology and computational pharmacology have enabled the identification of disease-associated molecular networks and the rational repositioning of FDA-approved drugs for new therapeutic indications [7,8]. Weighted gene co-expression network analysis (WGCNA) has emerged as a powerful approach for identifying co-expressed gene modules that reflect shared biological functions and regulatory mechanisms [9,10]. When integrated with single-cell transcriptomics, WGCNA can reveal cellular heterogeneity and cell-type-specific pathway dysregulation that may be obscured in bulk tissue analyses [11]. Furthermore, the integration of transcriptomic signatures with drug-target databases such as DrugBank and expression-based screening platforms like the Connectivity Map (CMap) enables systematic identification of therapeutic candidates that can reverse disease-associated gene expression patterns [12–14].

Previous studies have implicated interferon signaling, neutrophil activation, and complement pathways in APS pathogenesis [15–20]. However, these investigations have largely relied on candidate gene approaches or focused on individual cell types, lacking a comprehensive systems-level analysis that integrates multi-omics data to prioritize candidate therapeutic targets. Moreover, the molecular basis for patient-to-patient variability in APS remains poorly understood, limiting the development of precision medicine approaches [21–25].

Recent bioinformatics studies have begun to apply co-expression network approaches specifically to APS transcriptomics. A WGCNA-based analysis of whole blood RNA-seq data from thrombotic APS patients identified five co-expression modules, with STAT1 emerging as a central hub gene and promising pharmacological target through drug-gene interaction analysis [26]. Separately, an integrated bioinformatics and machine learning study employing WGCNA on neutrophil RNA-seq data (GSE102215) uncovered shared transcriptomic biomarkers (CCR1, MNDA, S100A8, CXCL2) between APS and recurrent miscarriage, nominating candidate therapeutic targets [27]. While these studies validate co-expression network analysis as a productive computational strategy in APS, they did not integrate single-cell RNA sequencing to characterize cellular heterogeneity, expression-based drug screening (CMap/LINCS) to identify candidate therapeutics, molecular docking for structural plausibility assessment, or patient-level molecular stratification. The present study addresses these gaps through a comprehensive multi-layer systems pharmacology framework, generating drug-repositioning hypotheses supported by concordance across independent datasets.

In this study, we employed an integrative systems pharmacology framework to: (i) identify core disease-associated gene modules through WGCNA of bulk transcriptomic data from neutrophils and whole blood; (ii) characterize cellular heterogeneity and module-specific functional states using single-cell RNA sequencing of B cells; (iii) stratify patients based on module expression profiles; (iv) perform network-based drug repurposing by integrating DrugBank target predictions and CMap expression-based screening; and (v) assess the structural plausibility of predicted drug-target interactions through molecular docking and corroborate module-level predictions in external datasets. Our approach prioritizes candidate targets across both ME10 (IFN-I) and ME2 (degranulation) pathways, suggesting diverse therapeutic opportunities — requiring experimental validation — that extend beyond traditional kinase inhibition strategies.

## Materials and methods

### Data acquisition and processing

Three transcriptomic datasets were obtained from GEO: neutrophil bulk RNA-seq (GSE102215; 9 APS, 9 controls), whole blood bulk RNA-seq (GSE205465; 60 APS, 28 controls), and single-cell B cell RNA-seq (GSE262240; 27,886 cells, comprising 26,936 B cells across seven subtypes plus 394 myeloid and 556 T cells retained for cross-lineage comparison) [28,29]. For the whole-blood dataset (GSE205465), gene-level counts were taken from the NCBI-generated raw count matrix provided by GEO (GSE205465_raw_counts_GRCh38.p13_NCBI), which contains 88 of the 91 samples registered for the series; NCBI's standardized re-quantification pipeline did not return counts for three samples (GSM6213171, GSM6213208, and GSM6213223), so these were not present in the count matrix used. Analyses were therefore performed on the resulting 88 samples (60 APS, 28 controls). To confirm that this data-source composition did not affect our findings, we independently re-quantified the raw sequencing reads for all 91 samples (STAR aligned to GRCh38); the differential-expression and module-trait results were essentially unchanged, with a genome-wide log2 fold-change concordance of r = 0.99 between the 88- and 91-sample analyses. Bulk data were normalized using DESeq2 (v1.38.0) [28], with variance-stabilizing transformation for downstream analyses. Single-cell data were processed with Seurat (v5.0.0) [29], normalized by SCTransform [30], batch-corrected using Harmony [31], and clustered via the Louvain algorithm. External validation datasets included GSE252972 (in vitro NAPc2 intervention in the MM1 monocytic cell line; 24 of 32 samples were analyzed — Control [unstimulated and IgG], aPL [the HL5B and HL7G anti-phospholipid antibodies], and aPL + NAPc2, 8 per group — whereas the NAPc2-alone and LPS conditions were not used), GSE252397 (mouse splenic dendritic cells; 5 saline and 5 NAPc2 samples), GSE212818 (platelet mRNA-seq), and GSE124565 (neutrophil methylation array) [32]. This study analyzed exclusively publicly available, fully de-identified human transcriptomic datasets from GEO; no new human or animal data were generated. Under institutional policy, secondary analysis of such de-identified public data does not require additional ethics committee approval or informed consent, and the original contributing studies obtained their respective ethical approvals and participant consent.

### WGCNA and functional enrichment

WGCNA was performed on the neutrophil dataset (n = 18; 9 APS, 9 controls) using a signed hybrid network (power β = 12, R² = 0.88) [9]. The purified-neutrophil cohort was chosen for module discovery because co-expression networks derived from whole blood are strongly confounded by inter-individual variation in leukocyte composition, whereas a homogeneous cell population yields modules that better reflect cell-intrinsic co-regulation; this design is also biologically motivated, given the central role of neutrophil effector programs (NETosis and degranulation) in APS. The larger whole-blood cohort (n = 88) was correspondingly reserved as an independent cohort for module-preservation testing. Although n = 18 is below the commonly recommended minimum of 20–30 samples, module robustness was confirmed by preservation analysis in the independent whole blood cohort (n = 88; Z-summary > 10 for both ME10 and ME2) [33]. Module-trait correlations were computed using Pearson correlation. Hub genes were identified by module membership (kME > 0.7) and gene significance (GS > 0.3). Module preservation was assessed with Z-summary scores (Z > 10 = strong preservation) [33]. Differential expression analysis used DESeq2 (|log_2_FC| > 0.5, adjusted P < 0.05) for bulk data and Wilcoxon rank-sum test for single-cell data [28,34]. GO and KEGG enrichment analyses were conducted using clusterProfiler (v4.8.0) [34].

### Drug repositioning and molecular docking

Drug-target interactions were extracted from DrugBank (v5.1.11) [12]. CMap/LINCS screening was performed using signatureSearch (v1.14.0) [13], with negative normalized connectivity scores (NCS) indicating disease signature reversal. Drug ranking used the best NCS per compound (lowest NCS across all profiled cell lines) to maximize sensitivity for drug candidate discovery; mean NCS values were computed as a sensitivity analysis and yielded highly concordant rankings (Spearman rho = 0.65 with original analysis). Molecular docking of four drugs spanning the APS treatment spectrum — prednisone (a corticosteroid reserved for severe or catastrophic APS), hydroxychloroquine and low-dose aspirin (adjuncts to anticoagulation), and fondaparinux (a parenteral anticoagulant) — against 81 module target proteins was performed using AutoDock Vina (v1.2.0) [35] with AlphaFold-predicted structures [36], Open Babel (v3.1.1) [37] for format conversion, and fpocket-detected binding pockets. Twenty expression-matched non-module proteins served as negative controls. Representative complexes were visualized using PyMOL (v3.2.0) [38].

### Machine learning and additional analyses

Diagnostic modeling used the 77 ME10/ME2 core genes present on both transcriptomic platforms as candidate features (training: GSE205465 whole blood, n = 88; independent validation: GSE102215 neutrophils, n = 18). Features were standardized (z-score) using training-set means and standard deviations, which were then applied unchanged to the validation set to avoid information leakage. LASSO logistic regression (glmnet; family = binomial; alpha = 1) was fitted with five-fold internal cross-validation, and the penalty parameter maximizing cross-validated AUC (lambda.min) was selected; genes with non-zero coefficients at lambda.min defined the sparse diagnostic signature. A Random Forest classifier (randomForest; 1,000 trees; mtry = sqrt(p); variable importance by mean decrease in accuracy) was trained on the same feature set as a non-linear comparator. Given the small validation cohort, overfitting was mitigated and assessed by (i) reporting out-of-bag error, (ii) repeated five-fold cross-validation (10 repeats) with mtry tuning over {3, 5, 8, 12} in the training cohort, and (iii) external validation in an independent tissue (neutrophils); the workflow and diagnostics are summarized in S16 Fig. The random seed was fixed at 2026 throughout. In silico SPI1 perturbation analysis used regression-based modeling on SCENIC-identified regulon targets. Methylation validation used Illumina 450K data (GSE124565; 10 APS, 12 controls) with limma-based differential methylation analysis [32]. Patient stratification employed median ME10/ME2 eigengene cutoffs to define four quadrants (Q1–Q4). All statistical tests were two-sided with significance at P < 0.05.

## Results

### WGCNA identifies two core disease modules in APS

**Fig 1:**
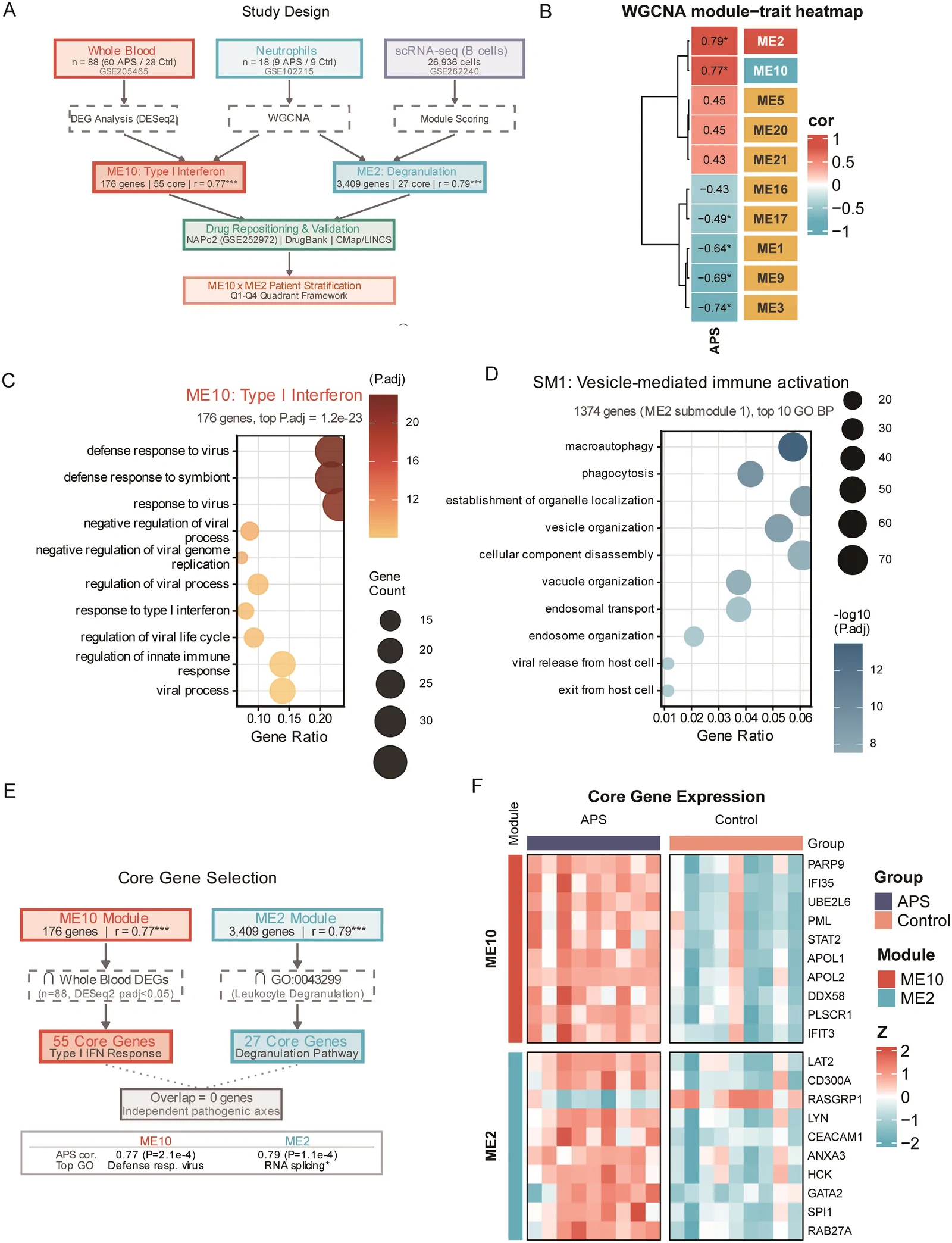
Discovery of ME10 and ME2 disease modules through WGCNA. **(A)** Study design schematic of the integrative multi-omics workflow: bulk RNA-seq from purified neutrophils (n = 18) underwent WGCNA for module discovery, with module preservation confirmed in whole blood (n = 88), single-cell RNA-seq (26,936 B cells) characterized cellular heterogeneity, and drug repositioning integrated DrugBank and CMap analyses with molecular docking for structural plausibility assessment. **(B)** WGCNA module-trait correlation heatmap: module eigengenes correlated with APS status, with ME2 (r = 0.79, P = 1.1 × 10^−4^) and ME10 (r = 0.77, P = 2.1 × 10^−4^) showing the strongest positive correlations. **(C)** GO biological process enrichment for ME10 (176 genes): type I interferon and antiviral defense terms predominate (top adjusted P = 1.2 × 10^−23^). **(D)** GO biological process enrichment for ME2 submodule SM1 (1,374 genes): vesicle-mediated immune activation, including macroautophagy, phagocytosis, and endosomal transport. **(E)** Core gene selection: intersecting ME10 (176 genes) with whole-blood DEGs yielded 55 core genes, and intersecting ME2 (3,409 genes) with leukocyte degranulation (GO:0043299) yielded 27 core genes, with zero overlap defining two distinct disease-associated modules. **(F)** Core gene expression heatmap: z-scored expression of ME10 and ME2 core genes across APS versus control samples.

To identify disease-associated gene networks in APS, we performed module discovery by WGCNA in the purified neutrophil cohort (n = 18: 9 APS patients, 9 controls) and then validated module preservation in the larger whole blood cohort (n = 88: 60 APS, 28 controls) (Fig 1a). Module-trait correlation analysis revealed two modules with the strongest positive association with APS status: ME2 (3,409 genes, r = 0.79, P = 1.1 × 10^−4^) and ME10 (176 genes, r = 0.77, P = 2.1 × 10^−4^) (Fig 1b; S1 and S2 Tables).

Gene Ontology enrichment analysis revealed that ME10 was significantly enriched in interferon-I (IFN-I) signaling pathways, with “defense response to virus” as the top term (adjusted P = 1.2 × 10^−23^), followed by “response to type I interferon” and “regulation of viral process” (Fig 1c). ME2, owing to its large size (3,409 genes), showed broad functional enrichment with top terms including “RNA splicing” (P = 2.9 × 10^−12^), “macroautophagy” (P = 5.6 × 10^−12^), and “vesicle organization” (P = 1.1 × 10^−10^) (Fig 1d; S4 Table). Sub-module analysis of ME2 using TOM-based hierarchical clustering (deepSplit = 3, minClusterSize = 100) identified SM1 (1,374 genes, r=+0.782 with APS) as the functionally relevant core enriched for phagocytosis and vesicle-mediated immune activation, with leukocyte degranulation ranking 51st (P = 2.9 × 10^−5^) among SM1 GO terms (S2 Fig).

To define core gene sets for downstream analyses, we employed two complementary approaches (Fig 1e). For ME10, intersection of 176 module genes with whole blood differentially expressed genes (DESeq2, padj<0.05) yielded 55 data-driven core genes. For ME2, a functionally-guided selection was applied: 27 leukocyte degranulation genes (GO:0043299) were extracted from the module. This GO term was selected a priori based on APS's well-documented degranulation phenotype and neutrophil hyperactivation, rather than post-hoc from enrichment rankings. To confirm the biological coherence of this selection, 19 of 27 genes clustered within the disease-correlated SM1 sub-module (r = +0.785 with APS trait; S2 Fig), and their median module membership (kME) within ME2 was 0.71 (IQR: 0.65–0.79), comparable to ME10 hub gene kME values, supporting their status as high-confidence module members despite the hypothesis-guided strategy. The two core gene sets shared zero overlapping genes, distinguishing ME10 and ME2 as two distinct disease-associated modules. Expression profiling of the top 10 core genes from each module demonstrated coordinated upregulation in APS neutrophil samples compared to controls (Fig 1f; S3 and S14 Tables).

### Single-cell analysis identifies transitional B cells with myeloid-like transcriptional features

**Fig 2:**
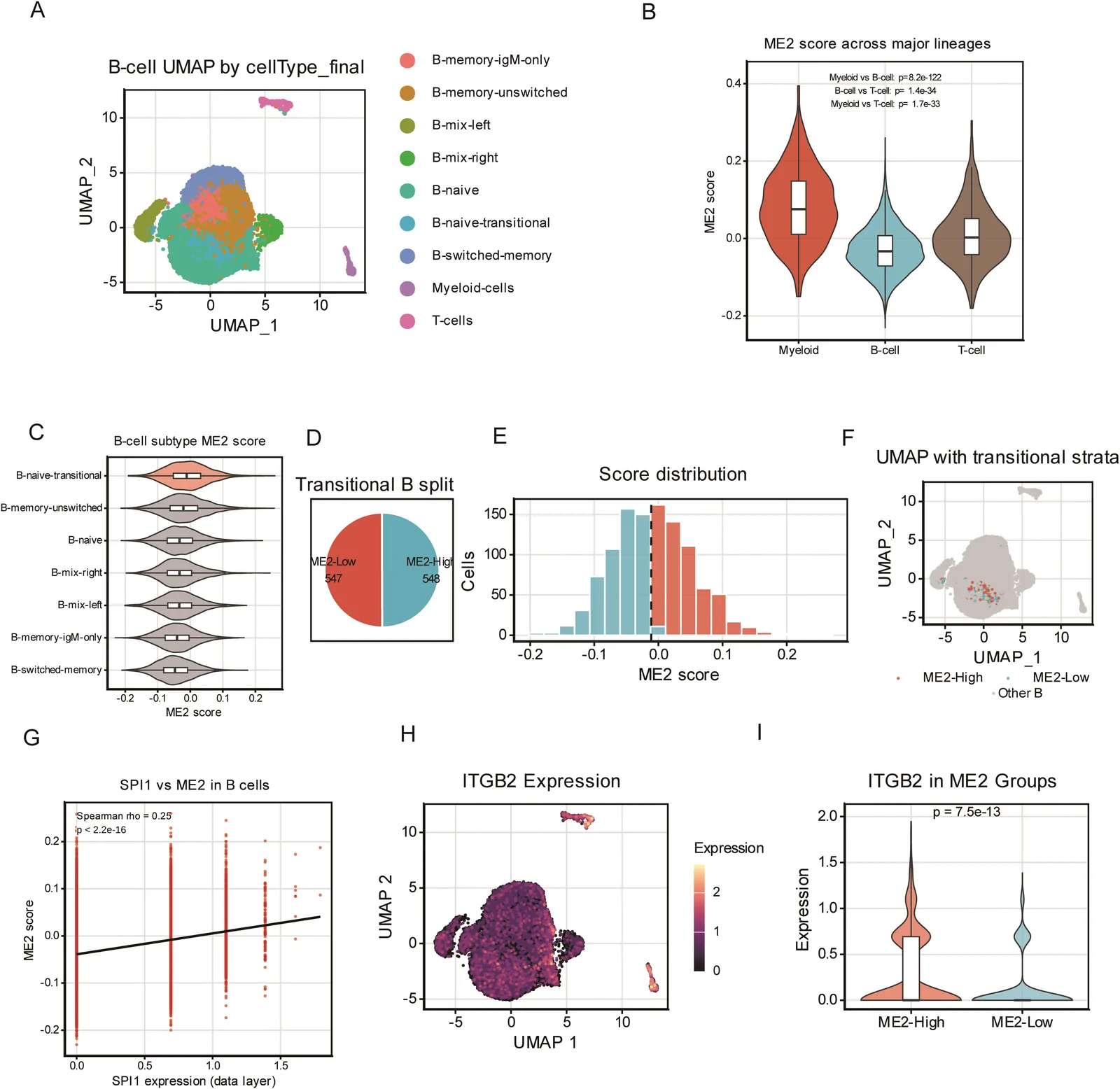
Single-cell profiling reveals myeloid-like B cell reprogramming. **(A)** UMAP of the B-cell-enriched dataset (27,886 cells: 26,936 B cells, 394 myeloid, and 556 T cells retained for cross-lineage comparison) colored by cell type. **(B)** ME2 score distribution comparing B cells and myeloid cells. **(C)** ME2 score heterogeneity within B cell subsets (transitional B cells highest). **(D)** Pie chart of transitional B cell ME2 stratification (ME2-High n = 548, ME2-Low n = 547). **(E)** ME2 score histogram with median cutoff. **(F)** UMAP overlay of ME2-High/Low transitional B cells. **(G)** SPI1 expression correlation with ME2 score (Spearman rho = 0.25, P < 2.2 × 10^−16^). (H) ITGB2 UMAP feature plot. (I) ITGB2 expression in ME2-High vs ME2-Low transitional B cells (P = 7.5 × 10^−13^).

To investigate cellular heterogeneity in ME2 pathway activation, we analyzed single-cell RNA-seq data from 26,936 B cells (GSE262240) (Fig 2a). ME2 degranulation module scores (AddModuleScore, 27 core genes) were calculated for each cell.

Comparison across major cell lineages confirmed that myeloid cells exhibited the highest ME2 scores (Wilcoxon P = 8.2 × 10^−122^ vs B cells), consistent with ME2 capturing a myeloid-associated transcriptional program (Fig 2b). Within B cell subtypes, transitional B cells (B-naive-transitional) showed the highest ME2 scores with a distinctive high-score tail, suggesting a subset with aberrant myeloid-like pathway activation (Fig 2c).

Median-based stratification of 1,095 transitional B cells yielded ME2-High (n = 548) and ME2-Low (n = 547) populations (Fig 2d–2f). SPI1 expression showed a statistically significant but modest positive association with ME2 module score (Spearman rho = 0.25, P < 2.2 × 10^−16^), consistent with, but not establishing, a regulatory role for this myeloid transcription factor [39,40] in ME2 pathway activation, though the modest effect size (R^2^ ≈ 6%) indicates that SPI1 alone does not fully explain ME2 heterogeneity (Fig 2g). At the single-gene level, ITGB2 (CD18) [41] expression was significantly elevated in ME2-High versus ME2-Low transitional B cells (Wilcoxon P = 7.5 × 10^−13^), providing gene-level support for the bulk-derived ME2 signature (Fig 2h–2i). Expression patterns of additional myeloid markers are shown in S15 Fig.

To explore whether SPI1 may contribute to myeloid-like gene expression patterns, we performed in silico perturbation analysis (S3, S4 Fig; S16 Table). Regression modeling predicted that 76 of 120 SPI1 regulon target genes would be significantly affected by simulated SPI1 knockout (FDR < 0.05), including myeloid kinases HCK and FGR. However, we note that these computational predictions require experimental validation (e.g., SPI1 knockdown in primary B cells) to establish a causal relationship.

### Functional characterization of ME2-high B cells

**Fig 3:**
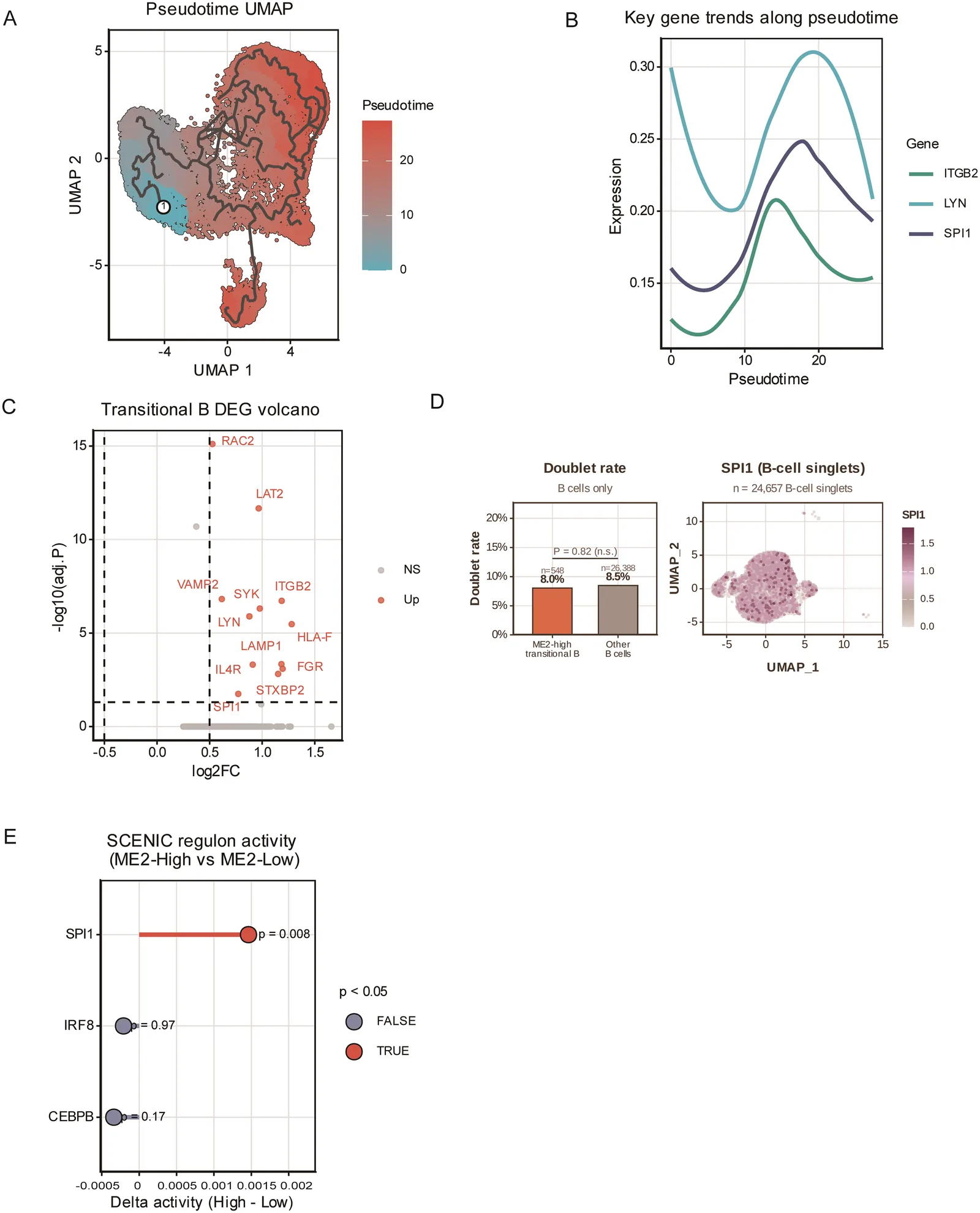
Functional characterization of ME2-high transitional B cells. **(A)** Pseudotime trajectory UMAP (Slingshot) showing B cell developmental progression from naive through transitional to myeloid-like states. **(B)** Key gene trends along pseudotime: smoothed expression of ITGB2, LYN, and SPI1 across pseudotime, reflecting progressive acquisition of myeloid-associated genes. **(C)** Volcano plot of differentially expressed genes in ME2-high versus ME2-low transitional B cells: 12 significantly upregulated genes (|log2FC| > 0.5, adjusted P < 0.05), including myeloid effectors RAC2, LAT2, VAMP2, SYK, ITGB2, LYN, LAMP1, HLA-F, IL4R, FGR, STXBP2, and SPI1. **(D)** Doublet exclusion validation (within the 26,936 B cells): ME2-high transitional B cells (n = 548) show a doublet rate (8.0%) not significantly different from other B cells (8.5%, n = 26,388; Fisher exact P = 0.82), with SPI1 expression shown in B-cell singlets only (n = 24,657). **(E)** SCENIC regulon activity in ME2-high versus ME2-low transitional B cells: the SPI1 regulon is significantly increased (delta activity = 0.0013, P = 0.008), whereas IRF8 (P = 0.97) and CEBPB (P = 0.17) are unchanged.

To further characterize the functional state and developmental trajectory of ME2-High transitional B cells, we performed pseudotime, differential expression, doublet exclusion, and regulon analyses (Fig 3).

Pseudotime trajectory analysis using Slingshot revealed a continuous developmental trajectory from naive B cells through transitional states (Fig 3a). Key myeloid-associated genes — ITGB2, LYN, and SPI1 — showed coordinated upregulation at intermediate pseudotime (range 10–20), consistent with progressive acquisition of myeloid-like features during B cell differentiation (Fig 3b; S5 Fig).

Differential expression analysis (Seurat FindMarkers, Wilcoxon test) between ME2-High and ME2-Low transitional B cells identified 12 significantly upregulated genes (|log_2_FC| > 0.5, adjusted P < 0.05) (Fig 3c; S5 Table). These included myeloid kinases (SYK, LYN, HCK, FGR), degranulation effectors [42] (RAC2, LAT2, VAMP2, LAMP1), immune regulators (IL4R, HLA-F, STXBP2), and the master myeloid transcription factor SPI1, representing coordinated activation of degranulation machinery and myeloid transcriptional programs. KEGG pathway enrichment confirmed significant enrichment in Fc gamma R-mediated phagocytosis and natural killer cell-mediated cytotoxicity (S13 Fig).

To address the potential concern that myeloid-like B cells represent doublet artifacts (B cell-myeloid cell fusions), we performed scDblFinder analysis on the 26,936 B cells. The ME2-high transitional B cells that display myeloid-like reprogramming (n = 548) had a doublet rate of 8.0%, not significantly different from other B cells (8.5%, n = 26,388; Fisher’s exact test P = 0.82, OR=1.06), indicating that the myeloid-like phenotype is unlikely attributable to doublets (Figs 3d, S1; S11 Table). SPI1 expression persisted in singlet-only analysis of the B-cell compartment (n = 24,657 cells).

SCENIC regulon activity analysis comparing ME2-High versus ME2-Low transitional B cells revealed that SPI1 regulon was significantly more active in ME2-High cells (delta activity = 0.0013, P = 0.008), while IRF8 (P = 0.97) and CEBPB (P = 0.17) showed no significant difference (Fig 3e; S6 Table). This association is consistent with, but does not by itself establish, a regulatory role for SPI1 in the myeloid-like transcriptional program (see also S3, S4 Fig for non-circular evidence and virtual knockout analyses).

### Network-based drug repositioning prioritizes candidate therapeutics

**Fig 4:**
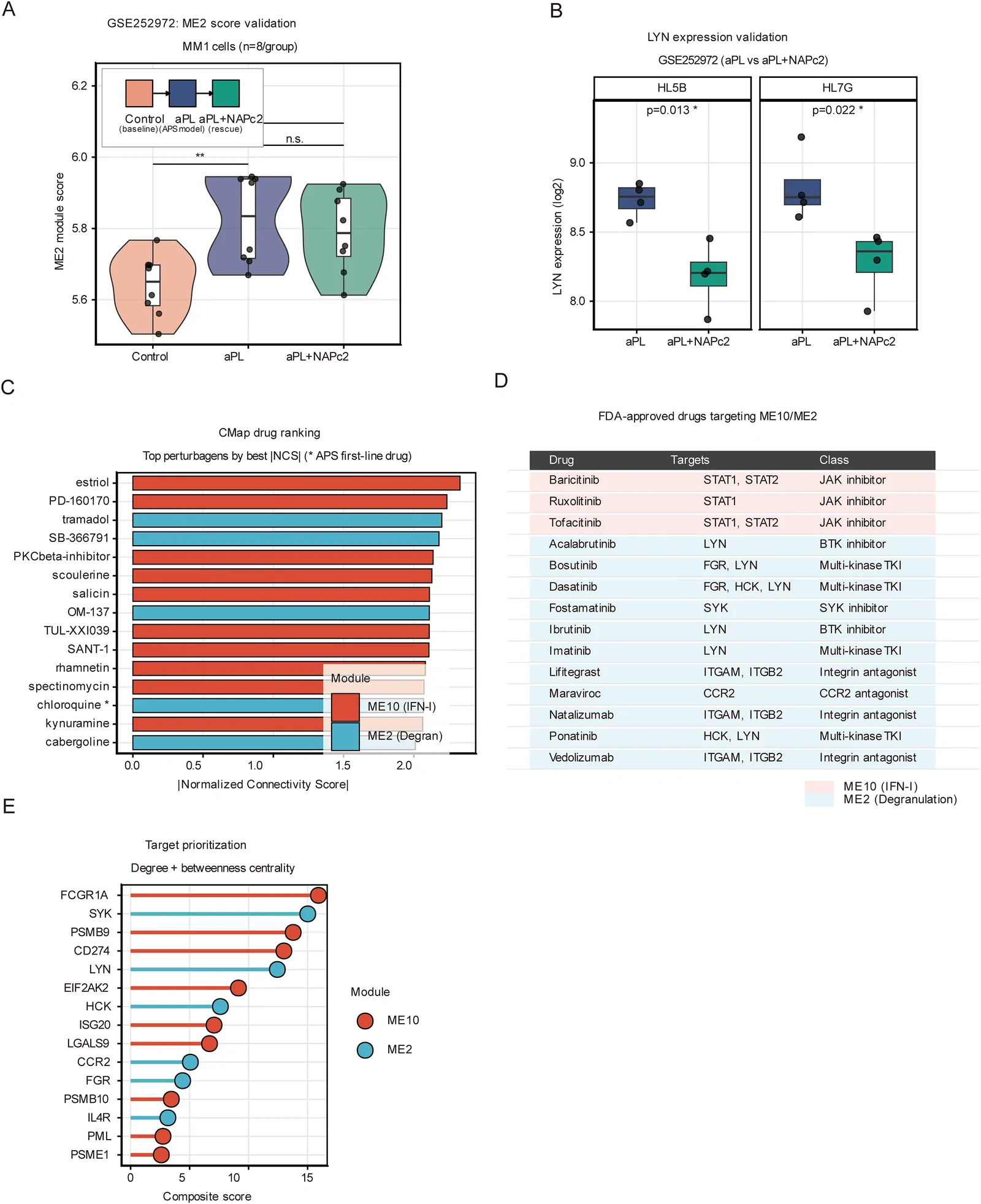
Drug repositioning and external validation. **(A)** External validation of the ME2 module score in an independent dataset (GSE252972): ME2 scores in MM1 monocytic cells (n = 8/group) across Control, aPL, and aPL + NAPc2 conditions; aPL stimulation elevates ME2 score (Wilcoxon P < 0.01) while NAPc2 co-treatment restores it toward baseline (aPL + NAPc2 vs Control: not significant). **(B)** Gene-level validation of LYN from GSE252972: LYN mRNA under the HL5B and HL7G anti-phospholipid antibody conditions, aPL versus aPL + NAPc2 (HL5B P = 0.013; HL7G P = 0.022, Wilcoxon test). **(C)** Connectivity Map (CMap/LINCS) drug ranking: top 15 perturbagens by best |NCS| (most negative NCS across cell lines), colored by module (red = ME10, cyan = ME2). Chloroquine (asterisk) — a 4-aminoquinoline antimalarial closely related to hydroxychloroquine, the guideline-recommended adjunct in APS — ranks #4 among ME2 candidates and #13 overall (NCS = −2.07, ME2); other top candidates include estriol (NCS = −2.33, ME10), PD-160170 (NCS = −2.24, ME10), and tramadol (NCS = −2.20, ME2). **(D)** FDA-approved drugs targeting ME10/ME2 core genes: 14 approved drugs grouped by module — ME10: 3 JAK inhibitors (Baricitinib, Ruxolitinib, Tofacitinib) targeting STAT1/STAT2; ME2: 4 multi-kinase TKIs (Dasatinib, Bosutinib, Ponatinib, Imatinib) targeting FGR/HCK/LYN, 2 BTK inhibitors (Ibrutinib, Acalabrutinib) targeting LYN, 1 SYK inhibitor (Fostamatinib), 3 integrin antagonists (Lifitegrast, Natalizumab, Vedolizumab) targeting ITGAM/ITGB2, and 1 CCR2 antagonist (Maraviroc); interactions from DrugBank (v5.1.11). (E) Target prioritization by protein-protein interaction network topology (STRING): top 15 drug-annotated candidate genes by composite score (degree + betweenness centrality), colored by module; top-ranked FCGR1A (ME10), SYK (ME2), PSMB9 (ME10), CD274 (ME10), and LYN (ME2).

To prioritize candidate therapeutics targeting ME10 and ME2 pathways, we performed systematic drug repositioning analysis combining external experimental validation, DrugBank target mapping, CMap expression-based screening, and network-based target prioritization (Fig 4).

External Validation. Analysis of the GSE252972 dataset showed that aPL stimulation elevated ME2 module scores in MM1 cells compared to control (P < 0.01), while NAPc2 co-treatment brought ME2 scores back toward baseline levels (aPL + NAPc2 vs Control: not significant), suggesting potential pharmacological reversibility of the ME2 pathway (Fig 4a). At the gene level, LYN expression was reduced by NAPc2 under both the HL5B (P = 0.013) and HL7G (P = 0.022) anti-phospholipid antibody conditions, though the fold changes were modest (~6.6%) (Fig 4b; S9 Table).

Connectivity Map Analysis. CMap analysis using best cell-line NCS (the most negative NCS per drug across cell lines, reflecting strongest disease signature reversal) ranked 2,282 annotated compounds. The top 15 candidates included estriol (NCS = −2.33, ME10), PD-160170 (NCS = −2.24, ME10), and notably chloroquine (NCS = −2.07, ME2) — a 4-aminoquinoline antimalarial closely related to hydroxychloroquine, the antimalarial recommended by current guidelines as an adjunct to anticoagulation in APS [4,5] — ranked 4th among ME2 candidates (13th overall), supporting the biological relevance of the CMap screen (Fig 4c). Top ME2-specific candidates included tramadol (NCS = −2.20) and cabergoline (NCS = −2.01). A balanced sensitivity analysis using equal-sized 27-gene hub sets for both modules yielded rankings concordant with the original analysis (Spearman rho = 0.653 across 8,140 shared perturbagens), indicating that the predominance of ME10-associated candidates was not driven by input gene-set size (S6 Fig).

DrugBank Analysis. Direct mapping of FDA-approved drugs to ME10/ME2 core genes identified 14 approved drugs with established drug-target relationships (Fig 4d; S7 Table): 3 JAK inhibitors targeting ME10 (Baricitinib [43–45], Ruxolitinib, Tofacitinib targeting STAT1/STAT2) [46]) and 11 drugs targeting ME2, including multi-kinase TKIs (Dasatinib [47], Bosutinib, Ponatinib, Imatinib targeting FGR/HCK/LYN), BTK inhibitors (Ibrutinib [48], Acalabrutinib targeting LYN), SYK inhibitor (Fostamatinib [49–51]), integrin antagonists (Lifitegrast, Natalizumab, Vedolizumab targeting ITGAM/ITGB2), and CCR2 antagonist (Maraviroc).

Target Prioritization. Network topology analysis of ME10/ME2 core genes in the STRING PPI network prioritized candidate targets by composite score (degree + betweenness centrality). Top-ranked targets included FCGR1A (ME10), SYK (ME2) [52], PSMB9 (ME10), CD274 (ME10), and LYN (ME2), combining high network centrality with database-annotated druggability (Fig 4e; S12 Fig; S8 and S13 Tables).

### Cross-tissue, cross-species, and epigenetic validation

Cross-tissue validation in an independent platelet mRNA-seq dataset (GSE212818; n = 3 APS, 3 controls) showed elevated ME2 activity in APS platelets (P = 0.032, Cohen’s d = 2.75), with ME10 showing a concordant trend (P = 0.097) (S14 Fig; S15 Table). In vivo validation using splenic DCs from NAPc2-treated lupus mice (GSE252397; n = 5/group) revealed significant suppression of the ME10 IFN-I program (GSEA NES = −1.53, P = 0.005).

Methylation analysis of an independent neutrophil cohort (GSE124565; 10 APS, 12 controls) identified 134 differentially methylated CpG probes (FDR < 0.05). Among ME2 genes, HCK was the only gene reaching significance (FDR = 0.041), converging with its identification as a candidate SPI1-associated target in virtual knockout analysis (S8, S9 Fig; S17 Table).

### Patient stratification and diagnostic model

**Fig 5:**
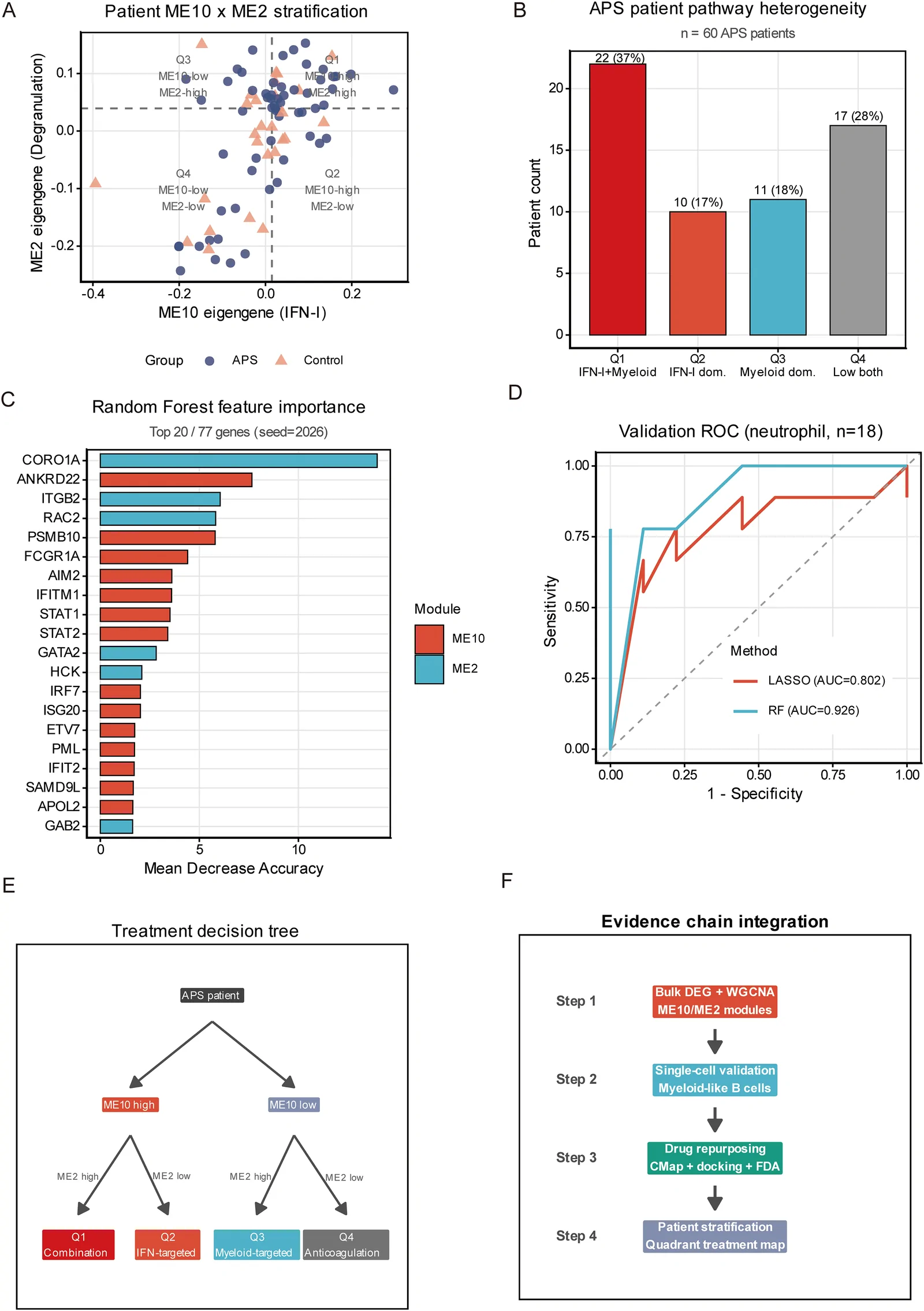
Computational patient stratification and candidate pathway mapping. **(A)** Patient-level ME10 × ME2 stratification: scatter of module eigengene values for 88 whole blood samples (GSE205465; 60 APS, 28 controls), with median-based cutoffs defining Q1 (ME10-high/ME2-high), Q2 (ME10-high/ME2-low), Q3 (ME10-low/ME2-high), and Q4 (ME10-low/ME2-low). **(B)** Distribution of 60 APS patients across quadrants: Q1 (n = 22, 37%), Q2 (n = 10, 17%), Q3 (n = 11, 18%), Q4 (n = 17, 28%). **(C)** Random Forest feature importance: top 20 genes by mean decrease in accuracy (ntree = 1,000; mtry = 8; seed = 2026) trained on 77 ME10/ME2 core genes in whole blood (n = 88); top features CORO1A, ANKRD22, ITGB2, RAC2, and PSMB10. **(D)** Diagnostic ROC for independent validation: LASSO (3 genes: CORO1A, ANKRD22, IFITM1) versus Random Forest (77 genes); training set (GSE205465, n = 88; LASSO AUC = 0.857, RF AUC = 1.000) and neutrophil validation set (GSE102215, n = 18; LASSO AUC = 0.802, RF AUC = 0.926); Random Forest repeated five-fold cross-validation mean AUC = 0.757 ± 0.141, indicating that the higher apparent RF performance reflects overfitting. **(E)** Hypothesized, pathway-guided decision tree mapping quadrants to candidate strategies (requiring prospective clinical validation): Q1 → combined IFN-I and myeloid targeting; Q2 → JAK inhibitors; Q3 → TKIs/SYK inhibitors; Q4 → standard anticoagulation. **(F)** Evidence-chain integration: four-step framework (bulk DEG + WGCNA modules → single-cell myeloid-like B cell discovery → drug repurposing → quadrant-guided treatment mapping).

ME10 × ME2 stratification of 88 whole blood samples revealed pathway heterogeneity among 60 APS patients: Q1 (dual-activation, n = 22, 37%), Q2 (IFN-dominant, n = 10, 17%), Q3 (myeloid-dominant, n = 11, 18%), and Q4 (quiescent, n = 17, 28%) (Fig 5a–b; Supplementary S11 Fig; S10 Table). LASSO logistic regression selected a 3-gene diagnostic signature (CORO1A, ANKRD22, IFITM1) achieving cross-tissue validation AUC = 0.802; Random Forest achieved AUC = 0.926 (Fig 5c, 5d). Notably, repeated five-fold cross-validation within the training cohort yielded a mean Random Forest AUC of 0.757 ± 0.141 (S16 Fig), consistent with overfitting given the small validation sample. This quadrant scheme is a hypothetical, computationally derived framework mapping each molecular subtype to candidate targeted therapies (Fig 5e, 5f). Immune infiltration analysis further demonstrated that ME10/ME2 module scores correlated with distinct immune cell profiles (S10 Fig; S12 Table). Because the quadrants are defined solely by transcriptomic signatures and were not linked to thrombosis, pregnancy morbidity, antibody profile, disease activity, or treatment response (clinical annotations were unavailable in the public datasets), this stratification represents a computational classification requiring prospective clinical validation before any clinical application.

### Molecular docking: structural plausibility assessment

**Fig 6:**
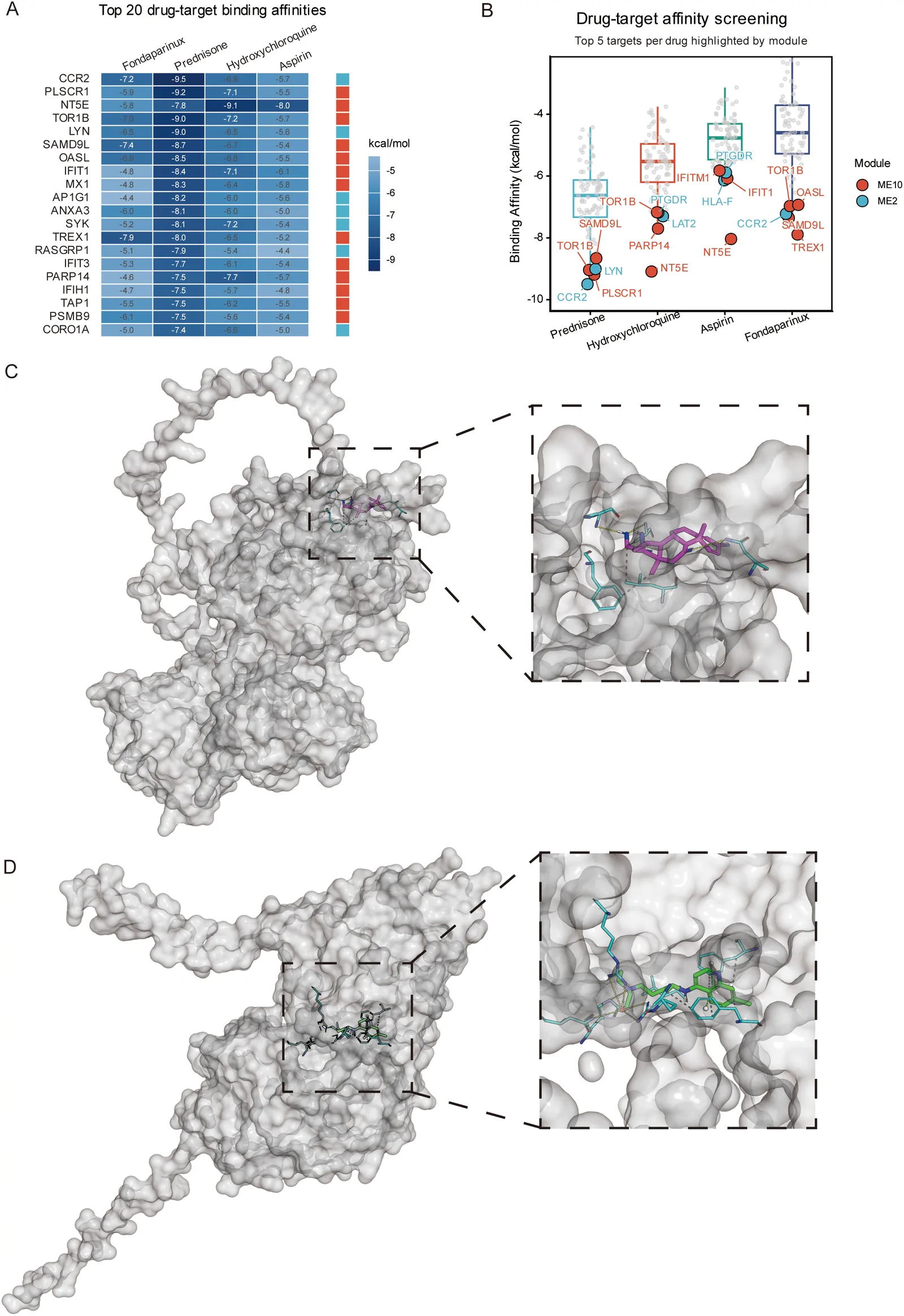
Molecular docking assesses structural plausibility of drug-target interactions. **(A)** Binding affinity heatmap of top 20 strongest drug-target pairs across four APS-relevant drugs and 81 module targets (54 ME10 + 27 ME2). Color indicates binding energy (kcal/mol). Strongest pairs: CCR2-Prednisone (−9.5), NT5E-HCQ (−9.1). **(B)** Box plot of binding affinity distributions for four drugs against all 81 targets, with lollipop annotations highlighting top ME10 and ME2 binding partners. **(C)** SYK-Prednisone 3D ghost-surface rendering (ΔG = −7.6 kcal/mol; AlphaFold structure; druggability = 0.612); full view and binding pocket zoom. **(D)** NT5E-Hydroxychloroquine 3D ghost-surface rendering (ΔG = −9.1 kcal/mol; AlphaFold structure; druggability = 0.639); full view and binding pocket zoom. Negative control analysis showed no module-specific binding selectivity (S7 Fig).

Systematic molecular docking of four APS-relevant drugs against 81 module targets revealed the strongest binding pairs as CCR2–Prednisone (ΔG = −9.5 kcal/mol) and NT5E–Hydroxychloroquine (ΔG = −9.1 kcal/mol) (Fig 6a). Prednisone showed the broadest strong binding profile across targets (Fig 6b). Representative 3D structures illustrated SYK–Prednisone (ΔG = −7.6 kcal/mol; Fig 6c) and NT5E–Hydroxychloroquine (ΔG = −9.1 kcal/mol; Fig 6d) binding modes. Negative control analysis using 20 expression-matched non-module proteins showed no module-specific binding selectivity (all Wilcoxon P > 0.05; S7 Fig; S18 Table), indicating that docking scores should be interpreted as purely computational, hypothesis-generating structural plausibility evidence rather than confirmation of target engagement or therapeutic activity.

## Discussion

This study presents a computational systems pharmacology framework integrating WGCNA, single-cell transcriptomics, CMap screening, and molecular docking to nominate candidate targets in APS [53,54]. Recent precision medicine initiatives in APS have underscored the need for molecularly guided therapeutic strategies [55], which our dual-module approach seeks to inform.

### Drug repositioning and structural plausibility

The present study extends recent WGCNA-based analyses in APS [26,27] by additionally integrating single-cell transcriptomics, CMap expression-based drug screening, molecular docking for structural plausibility assessment, and patient-level stratification. CMap analysis revealed a predominance of ME10-targeting candidates, reflecting the greater druggability of the IFN-I/JAK-STAT pathway [46,56,57] rather than differential pathogenic importance [58]; consistent with the balanced sensitivity analysis reported above, this predominance was robust to input gene-set size (S6 Fig). The recovery of chloroquine — the class-mate of hydroxychloroquine, which is used clinically as an adjunct in APS [4,5] — among top-ranked candidates supports the biological plausibility of the screen. Beyond established kinase inhibitors, exploratory targets emerged including the ROCK pathway (fasudil), adenosine A3 receptor (IB-MECA), and GSK-3β (AR-A014418). We emphasize that all repositioning candidates are computational, hypothesis-generating predictions requiring experimental and clinical validation before any therapeutic inference.

The clinical efficacy of heparinoids in APS has long been suspected to exceed their anticoagulant action, and one mechanistic possibility is modulation of the type I interferon axis through IRF7, its master transcriptional regulator. Our docking screen did not reveal strong binding between fondaparinux and IRF7, and a synthetic pentasaccharide acting indirectly through antithrombin would not be expected to engage an intracellular transcription factor directly; we therefore advance this connection as a literature-motivated hypothesis rather than a docking-supported finding. Nonetheless, given the documented pleiotropic immunomodulatory effects of heparinoids and recent epigenetic evidence of distinct inflammatory chromatin landscapes in APS monocytes [59], the possibility that antithrombotic agents modulate IFN-I signaling warrants dedicated experimental testing before any mechanistic or therapeutic claim can be made [60–62].

### Myeloid-like B cell reprogramming

The identification of transitional B cells with elevated ME2 scores and SPI1 expression suggests myeloid-like transcriptional features in a subset of APS B cells. Multiple lines of evidence converge on SPI1 as a candidate regulator — a modest positive correlation with the ME2 score, higher SCENIC regulon activity in ME2-high cells, and in silico perturbation (Results); these analyses are correlative and support association rather than experimentally established causal regulation. Recent characterization of CD19+CD14+ atypical B cells in SLE [63] and EBV-driven B cell reprogramming into antigen-presenting cells [64] support the broader concept of B cell functional plasticity in autoimmunity [65–67]. A concurrent single-cell study in thrombotic APS found transcriptional alterations concentrated in early B cell compartments [68], consistent with our ME2-high transitional B cell findings. Methylation validation independently identified HCK as the only ME2 gene with significant promoter methylation changes, consistent with a role in the SPI1-associated reprogramming axis [32].

### Patient stratification and candidate pathway mapping

The ME10 × ME2 stratification exposed substantial molecular heterogeneity among APS patients, spanning dual-pathway, IFN-predominant, degranulation-predominant, and quiescent subtypes. The parsimonious three-gene LASSO signature (CORO1A, ANKRD22, IFITM1) retained cross-tissue discriminative performance in the independent neutrophil cohort, whereas the non-linear Random Forest achieved a higher apparent AUC on the same data. We interpret this gap cautiously: with only 18 validation samples, the higher Random Forest performance is most consistent with optimistic bias and overfitting—indeed its cross-validated AUC within the training cohort was substantially lower than its apparent value—so the sparse, interpretable LASSO model is preferred as the candidate diagnostic signature, with Random Forest serving primarily to corroborate feature importance. Importantly, external validation relied on only 18 neutrophil samples drawn from a different biological source than the whole-blood training data; such cross-tissue validation demonstrates signal portability but cannot substitute for validation in an independent clinical cohort profiled from the same biological material. All diagnostic estimates should therefore be regarded as preliminary and require prospective validation in larger, clinically annotated cohorts. Concordant module activation in independent platelet and murine dendritic-cell datasets further supports cross-tissue and cross-species conservation of the ME10/ME2 programs [69–71].

### Limitations

Key limitations include: (i) computational predictions require prospective clinical validation; (ii) molecular docking provides supportive structural observations only and does not demonstrate physical binding, target engagement, or therapeutic efficacy — no molecular dynamics simulations, binding free-energy calculations, or functional assays were performed, and although the expression-matched negative-control panel showed no module-specific selectivity, the docking results should be regarded as supportive structural observations rather than an independent validation strategy; (iii) single-cell data were limited to B cells; (iv) ME2 (3,409 genes) is larger than typical WGCNA modules, though sub-module decomposition confirmed that 70% of core genes cluster within the disease-relevant SM1 (S2 Fig); and (v) the neutrophil WGCNA was performed with n = 18 samples, below the commonly recommended minimum of 20–30 for robust module detection; module preservation in the larger whole blood cohort (n = 88) partially mitigates this concern (Z-summary > 10); (vi) CMap analysis used best-NCS scoring to maximize discovery sensitivity, which may overestimate rankings compared with mean-NCS approaches; and (vii) the four-quadrant stratification is defined solely by transcriptomic signatures and was not associated with clinical parameters (thrombosis, pregnancy morbidity, antibody profile, disease activity, or treatment response), which were unavailable in the public datasets, so it should be regarded as a computational classification requiring prospective clinical validation rather than a clinically actionable tool.

Future directions include: (i) mechanistic studies to understand the estriol paradox in pregnancy-associated APS; (ii) prospective clinical validation of ME10-targeting agents in stratified patient populations; (iii) functional validation of ROCK, A3 receptor, and GSK-3β targeting in patient-derived cells or animal models; (iv) biomarker development for clinical implementation of ME10/ME2 stratification; (v) investigation of potential ME10 → ME2 hierarchical relationship; and (vi) clinical trials employing biomarker-driven patient stratification.

## Conclusions

This integrative systems pharmacology study prioritizes candidate therapeutic targets across ME10 (IFN-I) and ME2 (degranulation) pathways in APS, supported by the recovery of the antimalarial chloroquine among top CMap candidates and by cross-tissue/cross-species module conservation. The identification of SPI1-associated myeloid-like transcriptional features in B cells, together with an exploratory heparin–IRF7 hypothesis, provides computationally-derived mechanistic leads that warrant experimental validation. Patient-level ME10 × ME2 stratification offers a computational framework for generating pathway-guided treatment hypotheses, which require prospective clinical validation before any clinical application.

## Supporting information

S1 Fig Doublet exclusion quality control. (A) UMAP visualization of scDblFinder-predicted singlets and doublets across 27,886 cells (GSE262240). Doublets (n = 2,317, 8.31%) are uniformly distributed across clusters with no enrichment in specific cell populations. (B) Doublet rates across B cell subtypes. Bar plot shows subtype-level doublet rates from 2.7% (T-cells) to 14.7% (B-mix-right); the B-naive-transitional subset — which contains the ME2-high myeloid-like cells — shows an intermediate rate (8.1%), comparable to the overall average. (C) Myeloid marker expression in singlets only. FeaturePlots of CD14, CD33, ITGAM, and SPI1 restricted to 25,569 singlet cells. Myeloid marker expression persists in a subset of transitional B cells after doublet exclusion, confirming the biological authenticity of the myeloid-like B cell state.
(PDF)

S2 Fig ME2 sub-module decomposition. The ME2 module (3,409 genes) was decomposed using TOM-based hierarchical clustering (deepSplit = 2, minClusterSize = 50) into two sub-modules (SM1 and SM2). (A) Sub-module correlation with APS status. Bar plot of Pearson correlations between sub-module eigengenes and APS phenotype. SM1 (n = 2,079 genes) is positively correlated with APS (r = +0.785), while SM2 (n = 1,330 genes) is negatively correlated (r = −0.816). (B) Top 5 GO Biological Process terms per sub-module. Bubble plot comparing functional enrichment across SM1 and SM2. SM1 is enriched in immune-relevant vesicle-trafficking processes (vesicle-mediated transport, autophagy, process utilizing autophagic mechanism, cellular response to chemical stimulus, protein catabolic process); SM2 is dominated by RNA metabolic housekeeping processes (ncRNA metabolic process, ncRNA processing, tRNA metabolic process, rRNA processing, rRNA metabolic process). Bubble size indicates gene count; color indicates -log10(adjusted p-value). (C) Distribution of 27 core degranulation genes across sub-modules. Bar plot showing 23/27 (85%) core degranulation genes cluster in SM1, and 4/27 (15%) in SM2, supporting SM1 as the functionally coherent degranulation-enriched sub-module. Key SM1 genes include LYN, SPI1, ITGB2, RAC2, HCK, and CORO1A.
(PDF)

S3 Fig Non-circular SPI1-ME2 evidence. Independent evidence for SPI1-ME2 association, avoiding circular reasoning from module score computation. (A) ME2 module scores in SPI1-positive versus SPI1-negative B cells. Violin plot shows significantly higher ME2 scores in SPI1+ cells (Wilcoxon P = 5.2 x 10^-8). (B) GSEA of ME2 gene set in SPI1-ranked genes. Enrichment plot demonstrates significant enrichment of ME2 module genes among SPI1-correlated transcripts, independent of the original module scoring. (C) Volcano plot of SPI1+ vs SPI1- DEGs (excluding SPI1 itself). 150 nominally significant upregulated genes identified; 57/150 (38%) overlap with ME2 module genes. (D) Convergent evidence summary. Diagram showing four independent lines of evidence supporting SPI1-driven myeloid program: ME2 score (P = 5.2 x 10^-8), SCENIC regulon (P = 0.008), doublet exclusion (P = 0.82, n.s., confirming non-artifact), and nominal DEGs (57/150 ME2 overlap). Color indicates prior analysis (blue) versus current analysis (red).
(PDF)

S4 Fig SPI1 in silico perturbation (virtual knockout). Regression-based prediction of transcriptional changes upon SPI1 knockout in transitional B cells. (A) Waterfall plot of regression coefficients (Beta_SPI1) for 86 significantly affected genes (FDR < 0.05). Positive Beta indicates genes predicted to decrease upon SPI1 loss. Top affected genes: CRIP1 (Beta = 0.378), RAC2 (Beta = 0.133, ME2 gene), CORO1A (Beta = 0.112, ME2 gene), HCK (Beta = 0.098, ME2 gene). Bars colored by category: SPI1 regulon (dark blue), ME2 gene (red), immune activation (teal). (B) SPI1 expression versus ME2 module score scatter plot. Spearman rho = 0.191, P = 4.9 x 10^-31, confirming the linear relationship underlying the regression model. (C) Heatmap of SPI1 regulon gene expression comparing SPI1-High versus SPI1-zero transitional B cells. Clear separation of expression patterns validates the virtual knockout predictions.
(PDF)

S5 Fig Pseudotime supplementary analysis. (A) ME2 module score dynamics along pseudotime. Scatter plot with LOESS smoothing shows ME2 score increasing from early to intermediate pseudotime, consistent with progressive myeloid-like reprogramming. (B) Gene detection rate along pseudotime. Confirms that increasing ME2 scores are not confounded by technical dropout variation.
(PDF)

S6 Fig CMap balanced sensitivity analysis. Balanced CMap/LINCS analysis using equal-sized gene sets (27 hub genes each for ME10 and ME2) to control for input size bias. (A) Gene set comparison between original (55 ME10, 27 ME2) and balanced (27 ME10, 27 ME2) queries. (B) NCS rank correlation between original and balanced analyses. Spearman rho = 0.653 across 8,140 shared perturbagens, indicating good overall concordance. (C) Top 15 drug comparison. 11/20 top drugs are stable across both analyses (Jaccard = 0.38), confirming that the drug repositioning results are robust to gene set size effects.
(PDF)

S7 Fig Molecular docking negative control framework. Expression-matched non-module proteins (n = 20) were used as negative controls for molecular docking validation. (A) Binding affinity distributions for ME10 and ME2 target proteins. (B) Expression matching validation confirming control proteins have similar expression levels to module targets. (C) Docking comparison boxplots. ME10/ME2 target proteins versus expression-matched controls for three drugs (Aspirin, Hydroxychloroquine, Prednisone). No significant differences: Aspirin ME10 vs Ctrl P = 0.219, ME2 vs Ctrl P = 0.598; HCQ ME10 P = 0.519, ME2 P = 0.583; Prednisone ME10 P = 0.613, ME2 P = 0.707. Fondaparinux negative controls were not computed (P = NA) due to prohibitive docking time (MW ~ 1,728 Da). (D) Overall binding affinity distribution showing comparable affinity profiles across module and control proteins. Conclusion: docking scores reflect general drug-protein binding properties rather than module-specific selectivity.
(PDF)

S8 Fig GSE124565 methylation validation — module level. Independent neutrophil cohort (GSE124565, Illumina 450K methylation array; 10 APS, 12 controls) assessing promoter methylation of module genes. (A) ME10 module promoter methylation. Box plot comparing mean promoter beta values between APS and Control. Delta-beta = +0.0007, Wilcoxon P = 0.31 (not significant). (B) ME2 module promoter methylation. Delta-beta = +0.003, Wilcoxon P = 0.093 (borderline significant), suggesting a trend toward promoter hypermethylation in APS.
(PDF)

S9 Fig GSE124565 methylation validation — integration. (A) Expression-methylation integration scatter plot. Delta-beta (methylation change) versus log2FC (expression change) for module genes, showing the expected inverse relationship for a subset of genes. Spearman rho = −0.049. (B) Key gene promoter methylation heatmap. HCK (ME2 gene) shows significant differential methylation (FDR = 0.041, delta-beta = +0.007), with 347 upregulated DEGs showing concomitant promoter hypomethylation.
(PDF)

S10 Fig Immune infiltration analysis. (A) Immune cell type-module correlation heatmap. Pearson correlations between TIMER/Immunedeconv-estimated immune cell fractions and ME10/ME2 module scores in whole blood data (n = 88). (B) APS versus Control immune infiltration box plots. Comparison of estimated immune cell fractions across clinical groups.
(PDF)

S11 Fig Cell-level quadrant stratification. Original cell-level ME10 x ME2 stratification (moved from main Fig 5 to supplementary after patient-level analysis was adopted). (A) Cell-level ME10 x ME2 scatter plot. 27,886 B cells stratified into four quadrants by median ME10 and ME2 module scores. (B) Quadrant cell proportion distribution. Q1 24.1%, Q2 25.9%, Q3 25.9%, Q4 24.1% (approximately equal distribution at cell level).
(PDF)

S12 Fig PPI network and druggable hubs. (A) Protein-protein interaction network (STRING database) of ME10 and ME2 core genes. Network visualization highlights druggable hub nodes (degree >= 10, betweenness centrality in top quartile). Key hubs: STAT1 (degree = 43), IRF7 (degree = 33), DDX58 (degree = 32), SYK (degree = 14), LYN (degree = 12). Nodes colored by module (red = ME10, cyan = ME2) with druggable targets marked.
(PDF)

S13 Fig KEGG pathway enrichment and SCENIC regulon details. Panels originally in main Fig 3, moved to supplementary to accommodate pseudotime and doublet analyses. (A) KEGG pathway enrichment for ME2-High transitional B cell DEGs. Top enriched pathways include Fc gamma R-mediated phagocytosis and natural killer cell-mediated cytotoxicity, confirming myeloid/phagocytic features. (B) SCENIC regulon activity box plots. Detailed distribution of SPI1, CEBPB, and IRF8 regulon activity scores in ME2-High versus ME2-Low transitional B cells. See Table S6 for statistics.
(PDF)

S14 Fig Cross-tissue and cross-species validation. (A) GSE212818 platelet module scores. In platelet mRNA-seq (n = 3 APS, 3 controls), ME10 (26/176 genes detected) shows higher scores in APS (t-test P = 0.097, Cohen’s d = 2.08). ME2 (4/27 genes: LYN, STXBP2, PTGDR, RAB27A) is significantly elevated (P = 0.032, d = 2.75). (B) GSE212818 platelet heatmap. Z-score normalized expression of 30 platelet-detected module genes fully separates APS from control samples by hierarchical clustering. (C) GSE252397 GSEA of ME10 orthologs in mouse splenic dendritic cells (NAPc2 vs saline, n = 5/group). ME10: NES = −1.53, P = 0.005, confirming in vivo suppression of the IFN-I program by NAPc2. (D) GSE252397 GSEA of ME2 orthologs. ME2: NES = −1.33, P = 0.116, directionally concordant but not statistically significant. (E) GSE252397 volcano plot with module gene overlay. DESeq2 results for 14,150 expressed genes with ME10 (cyan) and ME2 (teal) orthologs highlighted. Notable downregulated genes: SCARF1 (ME10, P = 0.002), BATF2 (ME10, P = 0.021), CD300A (ME2, P = 0.019).
(PDF)

S15 Fig Myeloid marker FeaturePlots in B cells. Original Fig 2 myeloid marker content, moved to supplementary after replacement with focused ITGB2 validation (now Fig 2H, 2I). (A-D) UMAP feature plots of myeloid marker genes CD14 (A), CD33/SIGLEC3 (B), ITGAM/CD11b (C), and SPI1 (D) across all B cells (n = 26,936). Expression is concentrated in the Myeloid-cell cluster and a subset of transitional B cells, with overall low expression levels in most B cells. Combined with doublet exclusion (Fig 3D, Supplementary S1 Fig), these data support non-doublet-origin aberrant myeloid gene expression in transitional B cells.
(PDF)

S16 Fig Machine learning methodology. Diagnostic model development and overfitting diagnostics using the 77 ME10/ME2 core genes present on both transcriptomic platforms (training cohort: GSE205465 whole blood, n = 88; random seed = 2026 throughout). (A) LASSO logistic regression 5-fold cross-validation curve (cross-validated AUC versus log lambda), with the penalty parameters lambda.min and lambda.1se indicated; lambda.min was used to define the sparse signature. (B) LASSO coefficient shrinkage path showing the standardized coefficients of candidate genes across the lambda regularization range. (C) Random Forest out-of-bag (OOB) error as a function of the number of trees (1,000 trees; mtry = 8). (D) Random Forest hyperparameter tuning by repeated 5-fold cross-validation (10 repeats) over mtry values of 3, 5, 8, and 12 (mean AUC ± SD). The optimal mtry = 8 yielded a cross-validated AUC of 0.757 ± 0.141 in the training cohort, substantially below the apparent validation AUC of 0.926, consistent with overfitting given the small validation sample.
(PDF)

S1 Table Differentially expressed genes (DEG results). (CSV)

S2 Table WGCNA module summary. (CSV)

S3 Table ME10 and ME2 gene lists. (PDF)

S4 Table GO/KEGG enrichment. (CSV)

S5 Table Transitional B cell DEGs. (CSV)

S6 Table SCENIC regulon activity. (CSV)

S7 Table FDA-approved drug-target pairs. (CSV)

S8 Table Target druggability ranking. (CSV)

S9 Table GSE252972 external pharmacological validation (NAPc2 treatment). (A) NAPc2 gene-level response. (B) ME2 module validation scores. (C) LYN validation expression.
(XLSX)

S10 Table Patient-level ME10 × ME2 quadrant stratification. (A) Patient quadrant assignments. (B) Quadrant enrichment statistics.
(XLSX)

S11 Table scDblFinder doublet analysis. (A) Doublet rate by cell type. (B) Myeloid-like transitional B cell doublet enrichment test.
(XLSX)

S12 Table Immune infiltration–module correlations. (CSV)

S13 Table STRING PPI network analysis. (A) PPI network topology metrics. (B) PPI druggable hub genes.
(XLSX)

S14 Table ME10 hub genes. (CSV)

S15 Table Cross-dataset overlap genes. (CSV)

S16 Table SPI1 virtual knockout results. (CSV)

S17 Table Differential methylation (GSE124565). (CSV)

S18 Table Docking negative controls. (CSV)
