# Supplementary material for: Systems pharmacology reveals type I interferon and myeloid-like B cell reprogramming as candidate druggable axes in antiphospholipid syndrome
Source: PLoS One. 2026 Aug 26;21(8):e0349155. doi: 10.1371/journal.pone.0349155 (PMC13514046; doi:10.1371/journal.pone.0349155)
Supplement: Supplementary file 1 [file pone.0349155.s001.pdf]

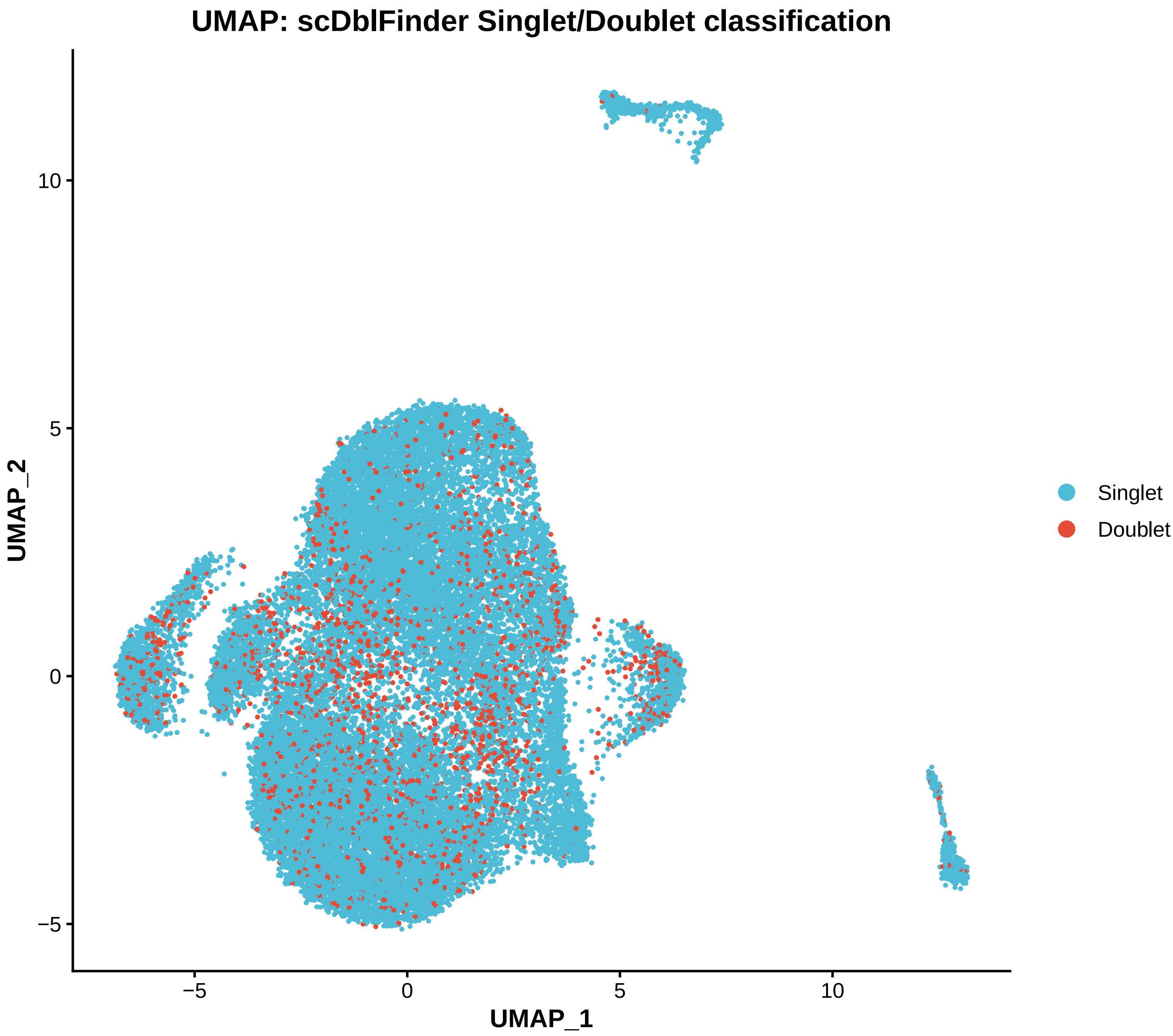
UMAP: scDblFinder Singlet/Doublet classification
UMAP_2
UMAP_1
10
5
0
−5
−5
0
5
10
Singlet
Doublet

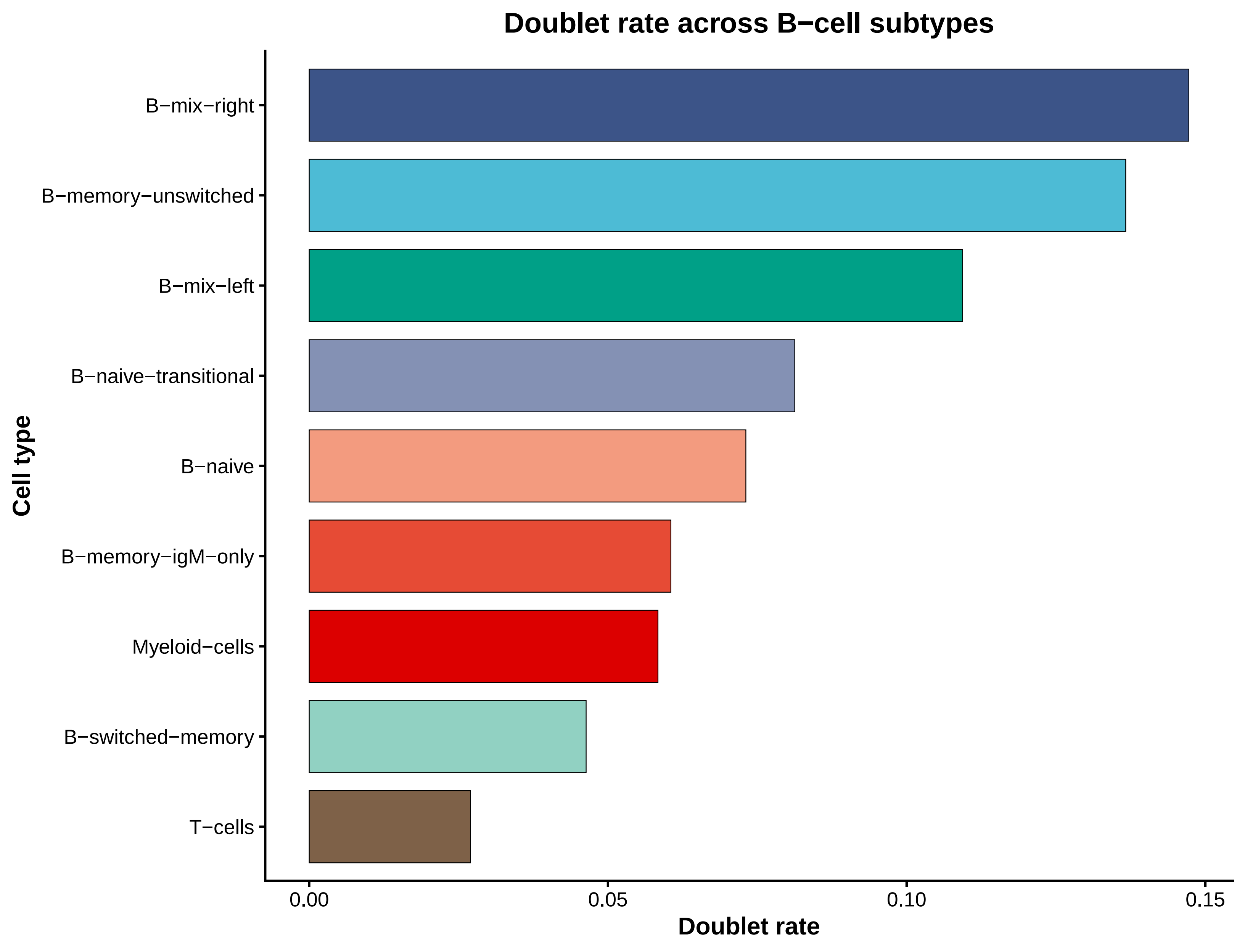
Doublet rate across B−cell subtypes
Cell type
B−mix−right
B−memory−unswitched
B−mix−left
B−naive−transitional
B−naive
B−memory−igM−only
Myeloid−cells
B−switched−memory
T−cells
0.00
0.05
0.10
0.15
Doublet rate

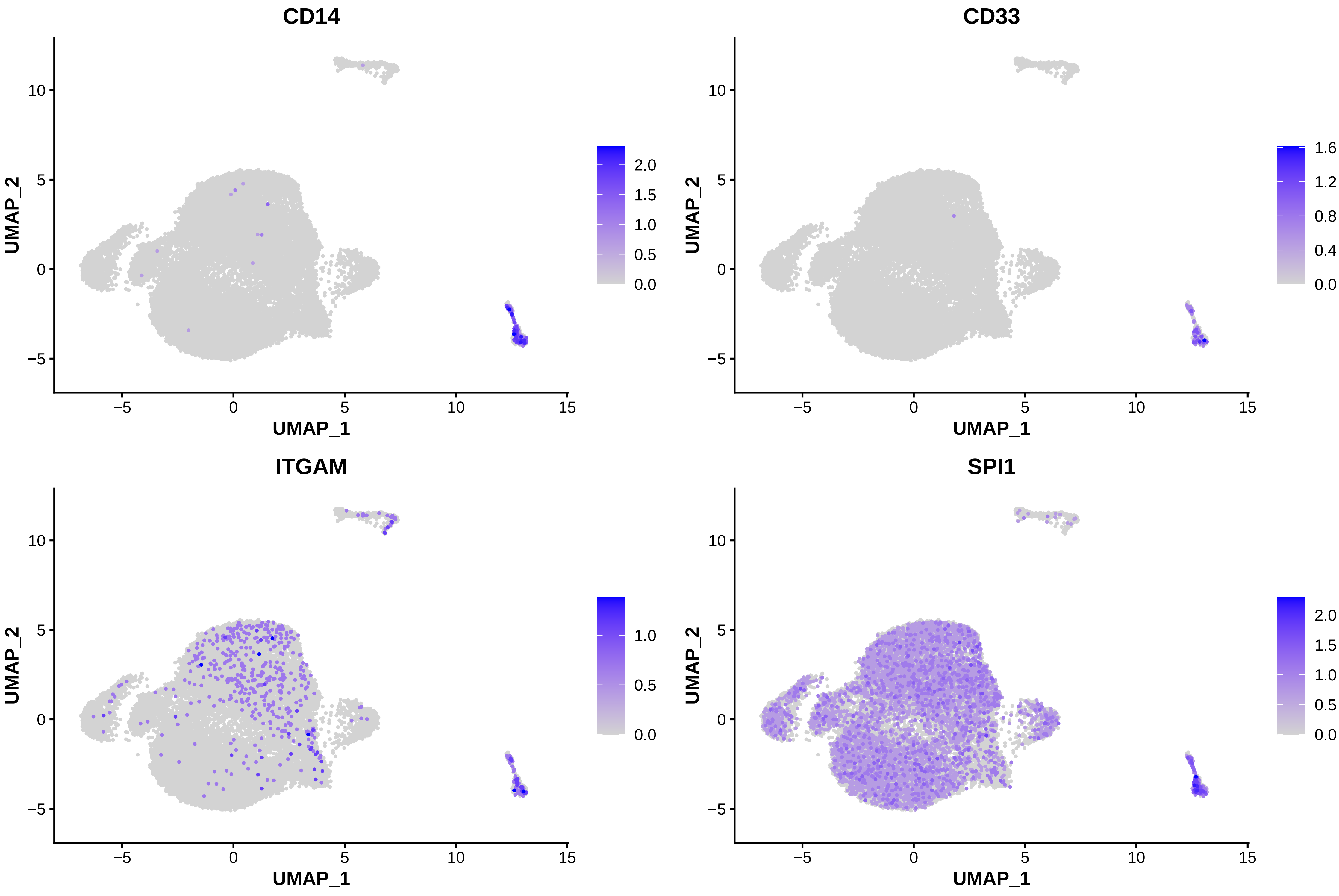

CD14
CD33
ITGAM
SPI1
UMAP_1
UMAP_2
