# Supplementary material for: Systems pharmacology reveals type I interferon and myeloid-like B cell reprogramming as candidate druggable axes in antiphospholipid syndrome
Source: PLoS One. 2026 Aug 26;21(8):e0349155. doi: 10.1371/journal.pone.0349155 (PMC13514046; doi:10.1371/journal.pone.0349155)
Supplement: Supplementary file 2 [file pone.0349155.s002.pdf]

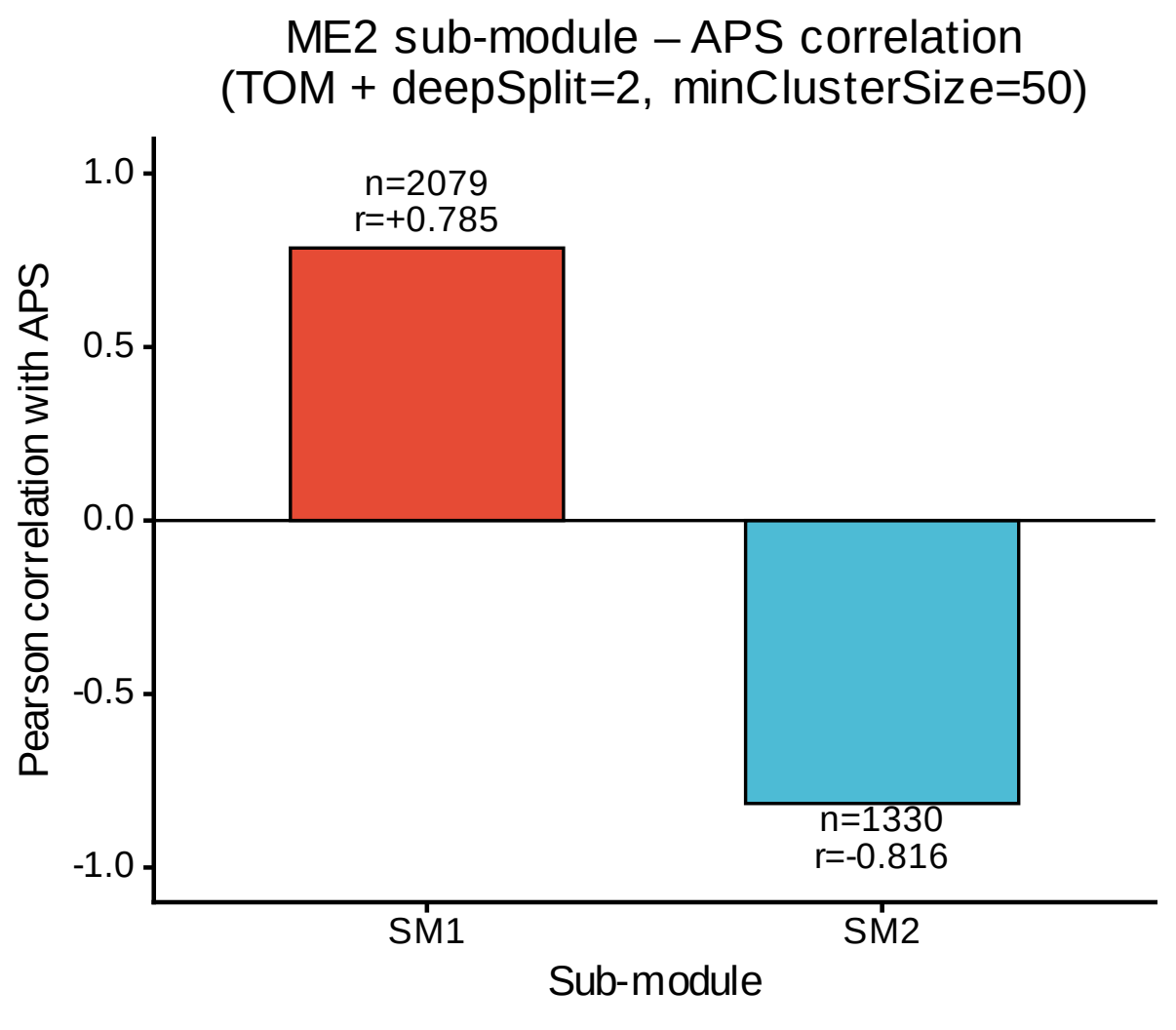

ME2 sub-module – APS correlation
(TOM + deepSplit=2, minClusterSize=50)
Pearson correlation with APS
1.0
0.5
0.0
-0.5
-1.0
n=2079
r=+0.785
n=1330
r=-0.816
SM1
SM2
Sub-module

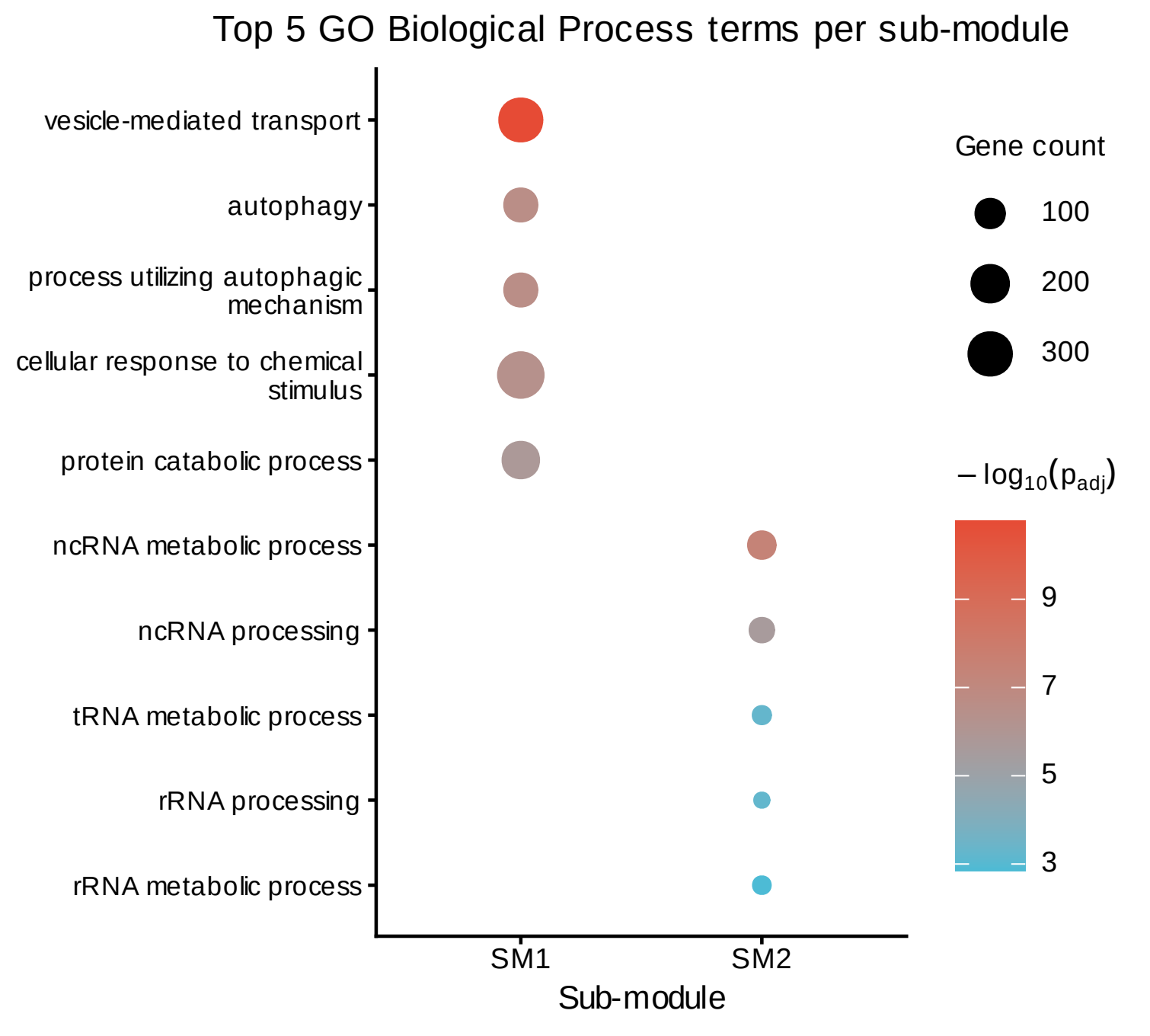

Top 5 GO Biological Process terms per sub-module
vesicle-mediated transport
autophagy
process utilizing autophagic mechanism
cellular response to chemical stimulus
protein catabolic process
ncRNA metabolic process
ncRNA processing
tRNA metabolic process
rRNA processing
rRNA metabolic process
SM1
SM2
Sub-module
Gene count
100
200
300
$-\log_{10}(p_{adj})$
9
7
5
3

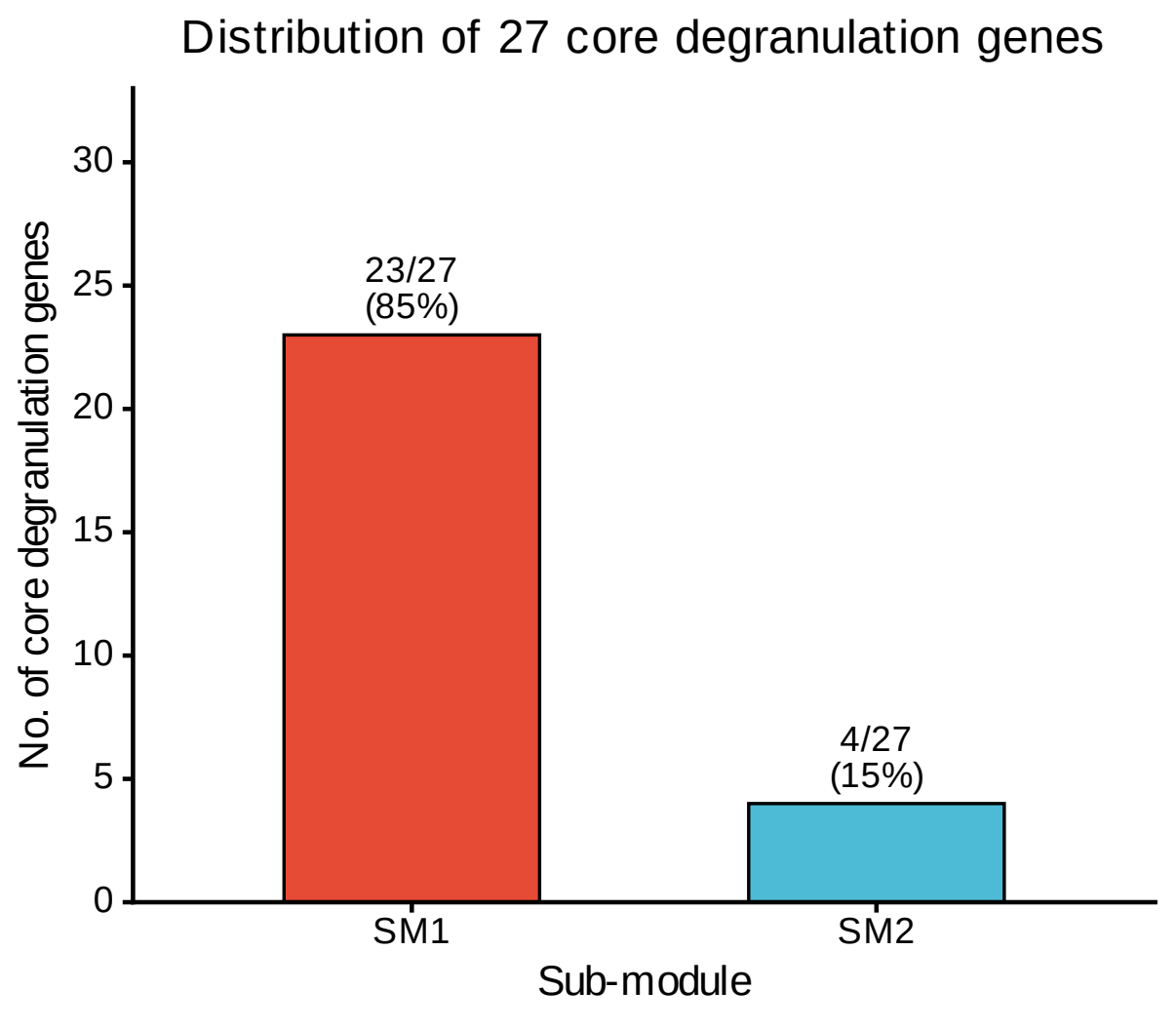
Distribution of 27 core degranulation genes
No. of core degranulation genes
0
5
10
15
20
25
30
23/27
(85%)
4/27
(15%)
SM1
SM2
Sub-module
