# Supplementary material for: Systems pharmacology reveals type I interferon and myeloid-like B cell reprogramming as candidate druggable axes in antiphospholipid syndrome
Source: PLoS One. 2026 Aug 26;21(8):e0349155. doi: 10.1371/journal.pone.0349155 (PMC13514046; doi:10.1371/journal.pone.0349155)

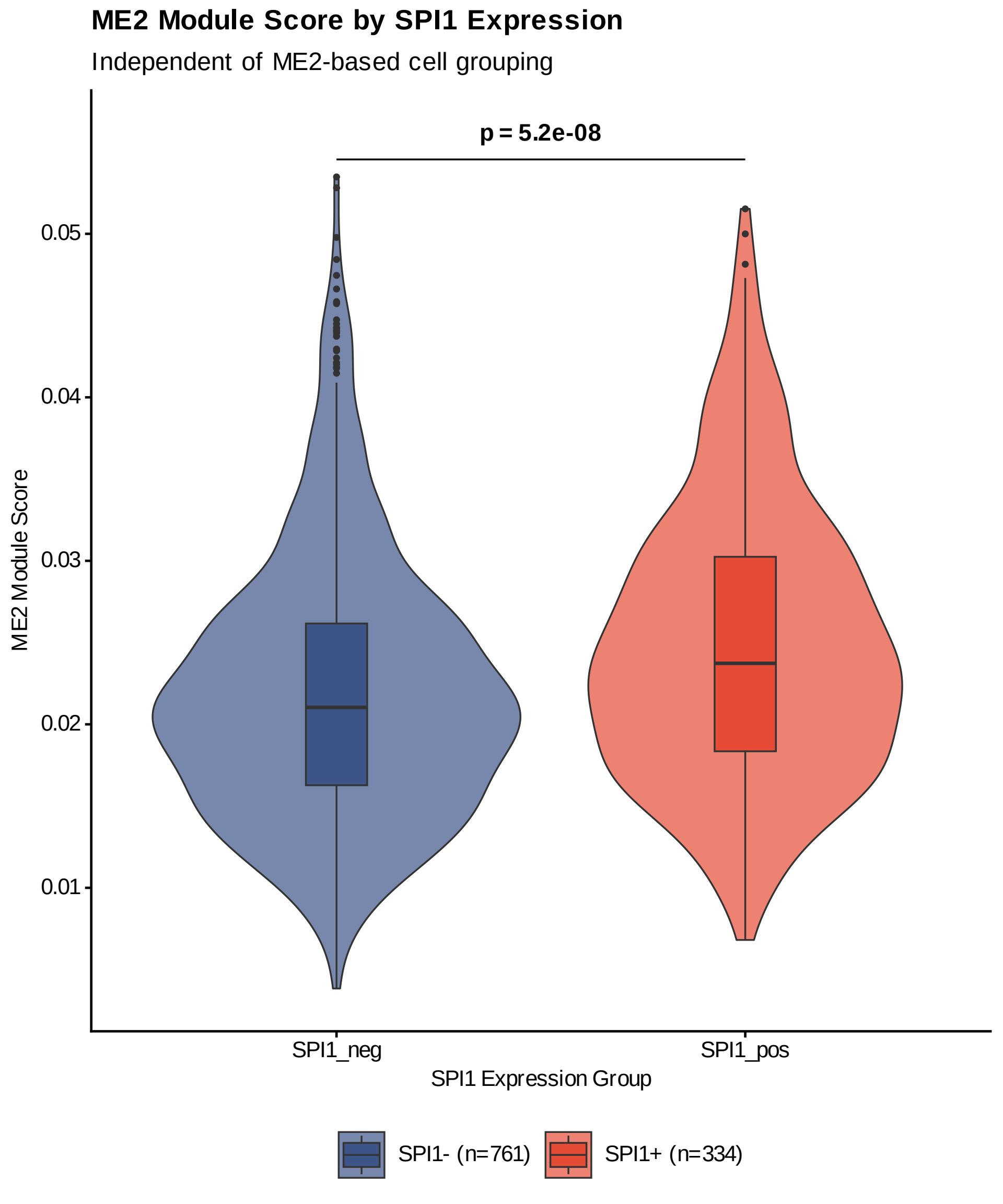
ME2 Module Score by SPI1 Expression
Independent of ME2-based cell grouping
p = 5.2e-08
0.05
0.04
0.03
0.02
0.01
ME2 Module Score
SPI1_neg
SPI1_pos
SPI1 Expression Group
SPI1- (n=761)
SPI1+ (n=334)

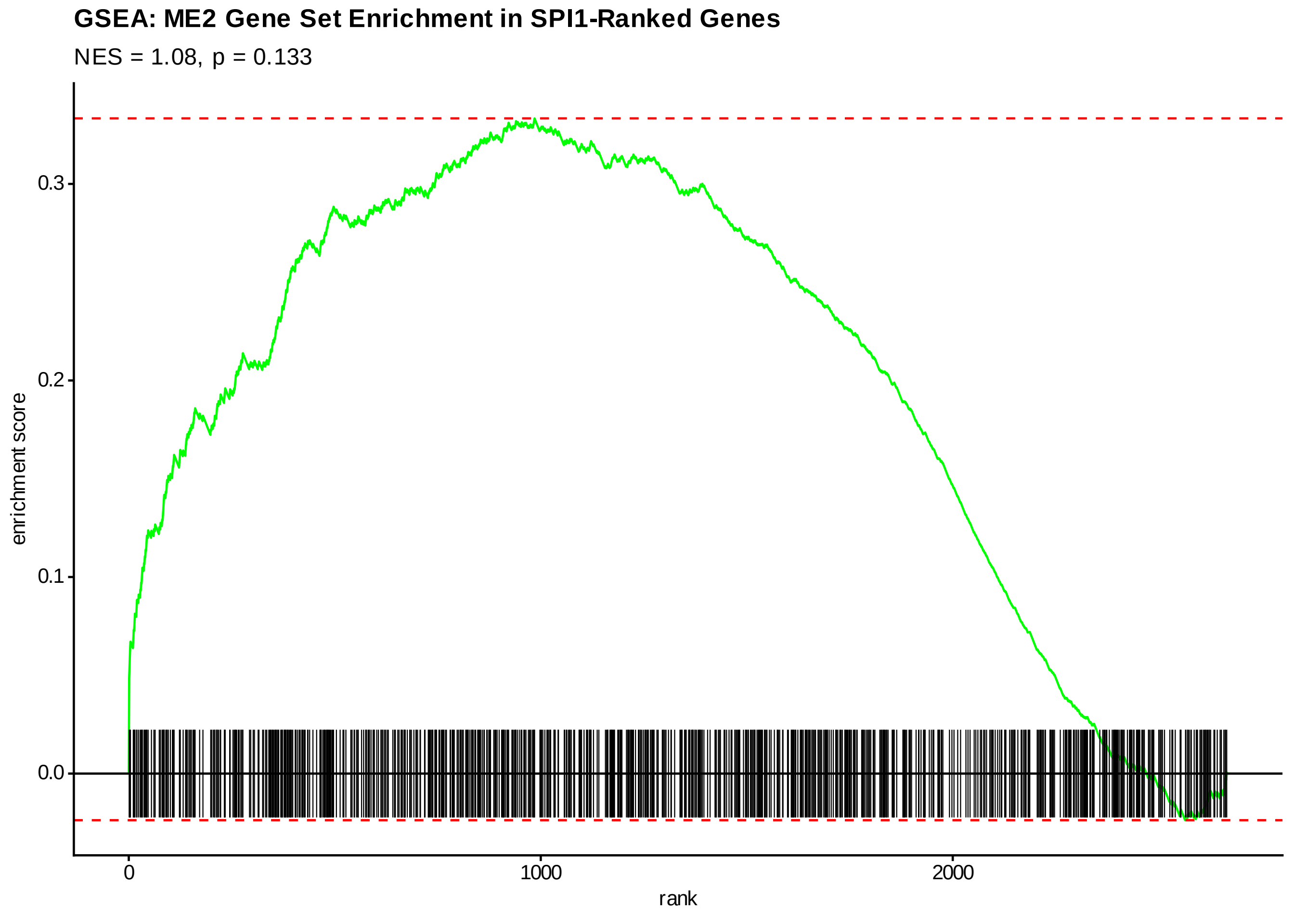
GSEA: ME2 Gene Set Enrichment in SPI1-Ranked Genes
NES = 1.08, p = 0.133
enrichment score
0.3
0.2
0.1
0.0
0
1000
2000
rank

SPI1+ vs SPI1- Transitional B Cells (SPI1 Excluded)

Volcano excludes SPI1 (grouping variable); nominal p < 0.01; 150 upregulated genes

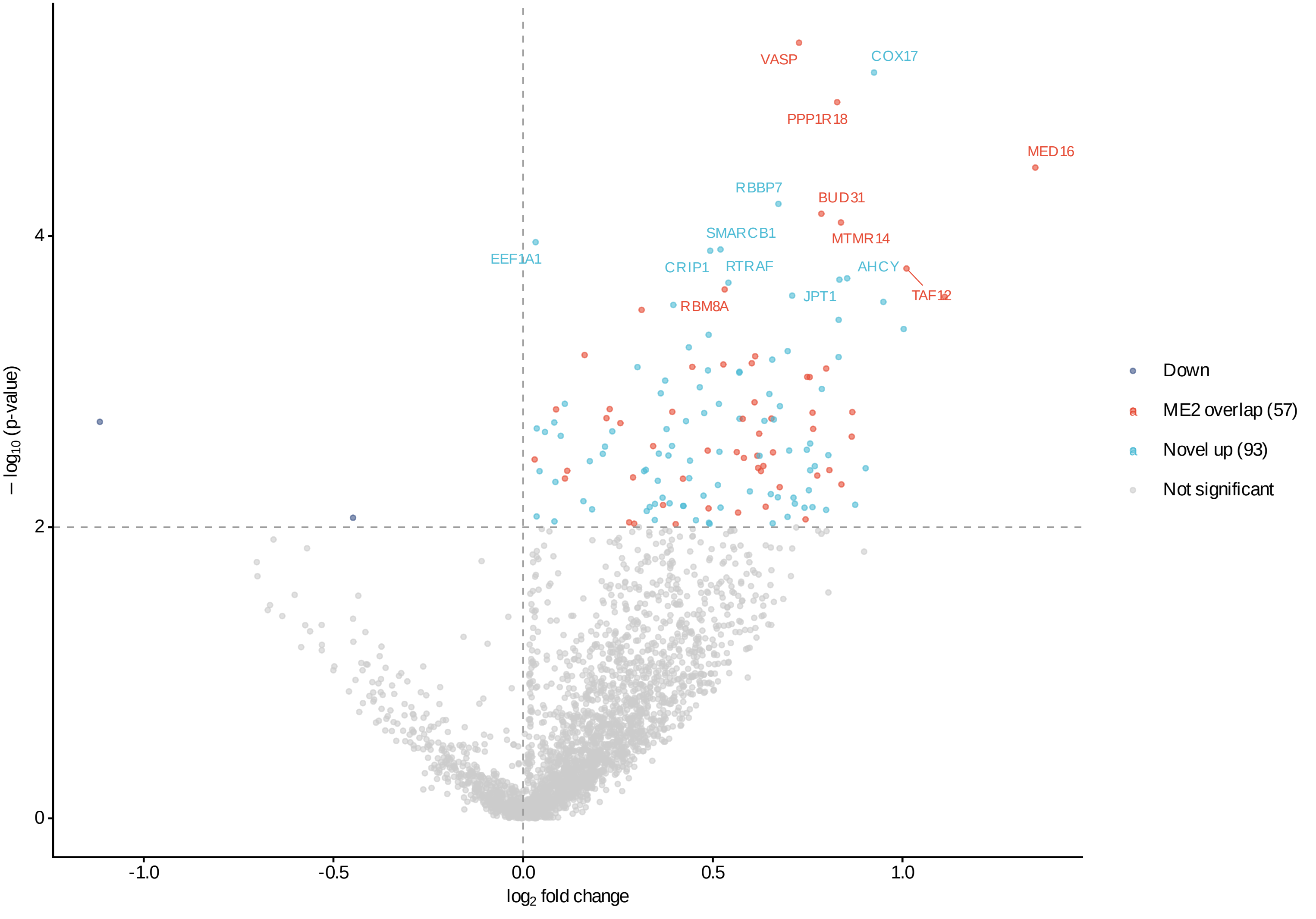

Convergent Evidence for SPI1-Driven Myeloid Program

Independent lines of evidence, no circular reasoning

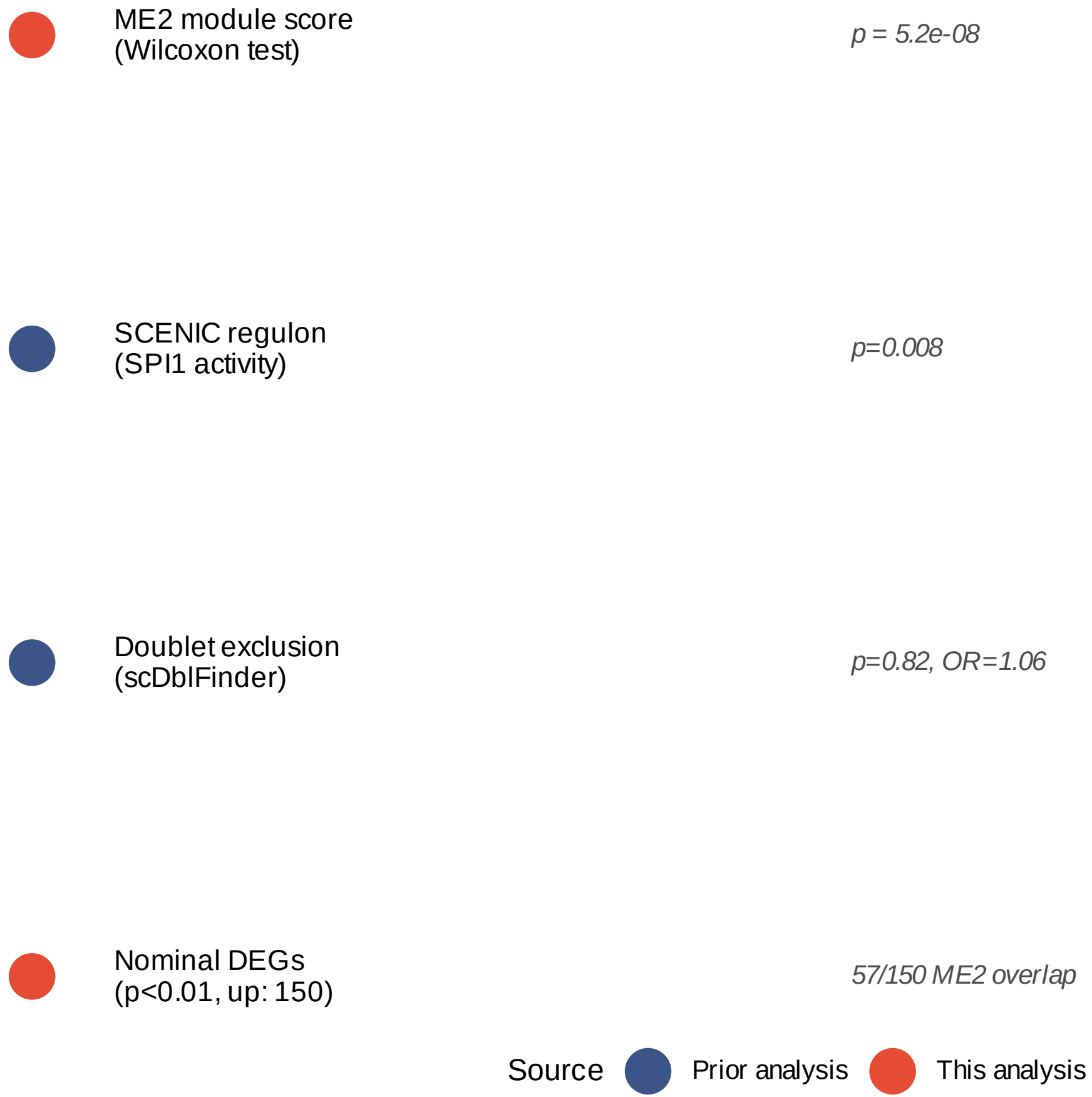

Supplement: Supplementary file 3 [file pone.0349155.s003.pdf]
