# Supplementary material for: Systems pharmacology reveals type I interferon and myeloid-like B cell reprogramming as candidate druggable axes in antiphospholipid syndrome
Source: PLoS One. 2026 Aug 26;21(8):e0349155. doi: 10.1371/journal.pone.0349155 (PMC13514046; doi:10.1371/journal.pone.0349155)
Supplement: Supplementary file 4 [file pone.0349155.s004.pdf]

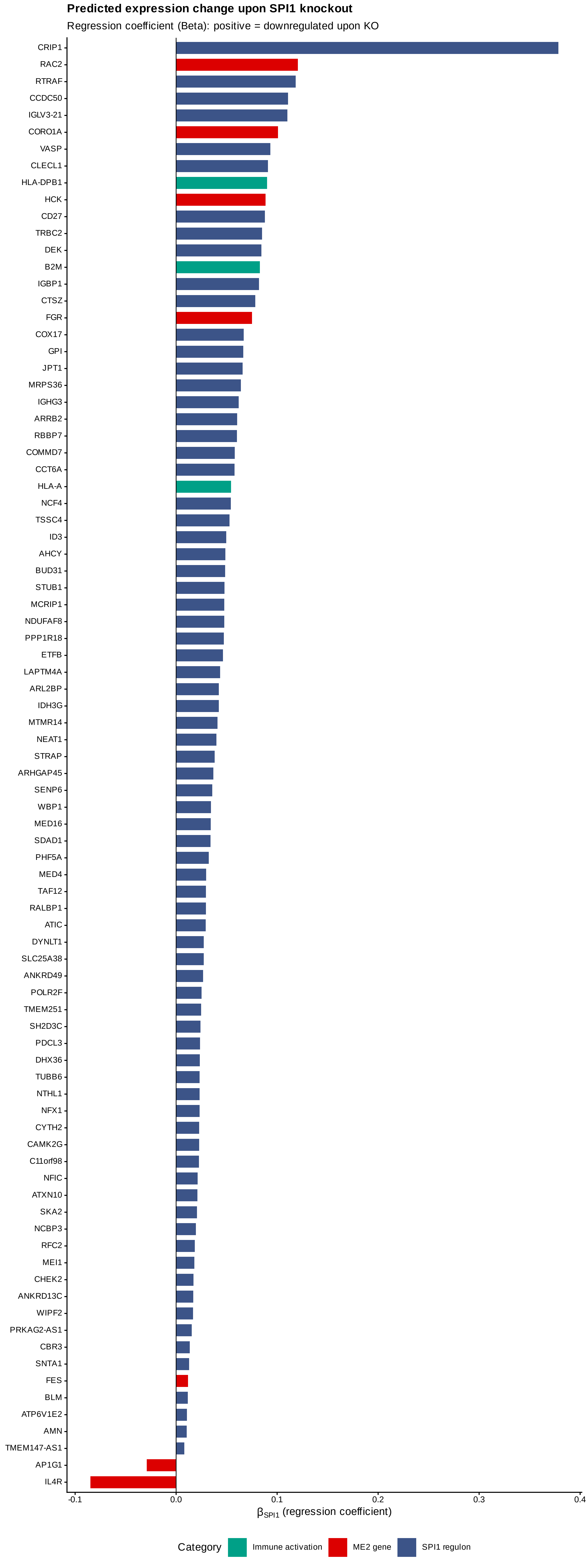

Predicted expression change upon SPI1 knockout
Regression coefficient (Beta): positive = downregulated upon KO
CRIP1
RAC2
RTRAF
CCDC50
IGLV3-21
CORO1A
VASP
CLECL1
HLA-DPB1
HCK
CD27
TRBC2
DEK
B2M
IGBP1
CTSZ
FGR
COX17
GPI
JPT1
MRPS36
IGHG3
ARRB2
RBBP7
COMMD7
CCT6A
HLA-A
NCF4
TSSC4
ID3
AHCY
BUD31
STUB1
MCRIP1
NDUFAF8
PPP1R18
ETFB
LAPTM4A
ARL2BP
IDH3G
MTMR14
NEAT1
STRAP
ARHGAP45
SENP6
WBP1
MED16
SDAD1
PHF5A
MED4
TAF12
RALBP1
ATIC
DYNLT1
SLC25A38
ANKRD49
POLR2F
TMEM251
SH2D3C
PDCL3
DHX36
TUBB6
NTHL1
NFX1
CYTH2
CAMK2G
C11orf98
NFIC
ATXN10
SKA2
NCBP3
RFC2
MEI1
CHEK2
ANKRD13C
WIPF2
PRKAG2-AS1
CBR3
SNTA1
FES
BLM
ATP6V1E2
AMN
TMEM147-AS1
AP1G1
IL4R
-0.1
0.0
0.1
0.2
0.3
0.4
β_{SPI1} (regression coefficient)
Category
Immune activation
ME2 gene
SPI1 regulon

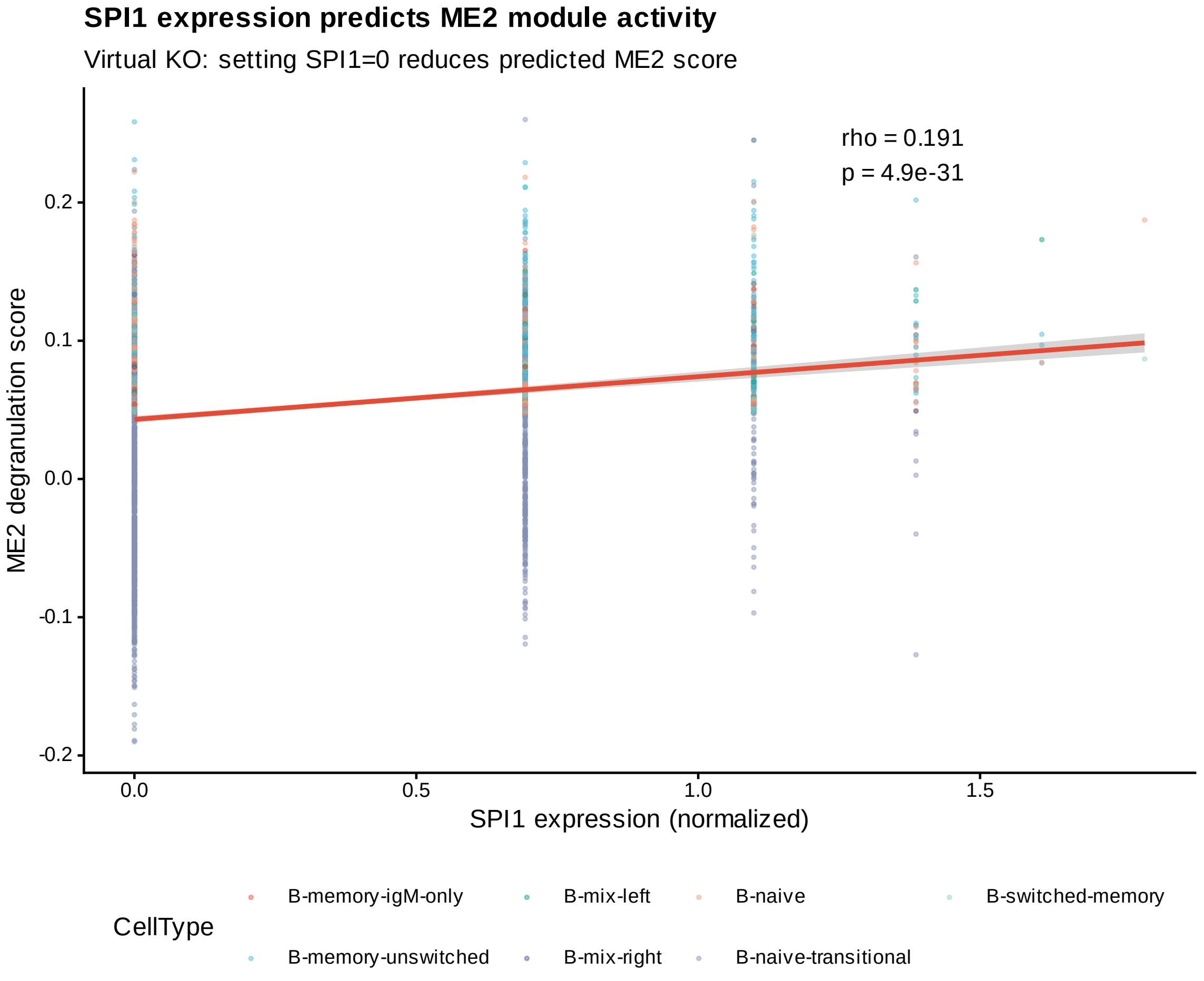
SPI1 expression predicts ME2 module activity
Virtual KO: setting SPI1=0 reduces predicted ME2 score
rho = 0.191
p = 4.9e-31
ME2 degranulation score
0.2
0.1
0.0
-0.1
-0.2
0.0
0.5
1.0
1.5
SPI1 expression (normalized)
CellType
B-memory-igM-only
B-mix-left
B-naive
B-switched-memory
B-memory-unswitched
B-mix-right
B-naive-transitional

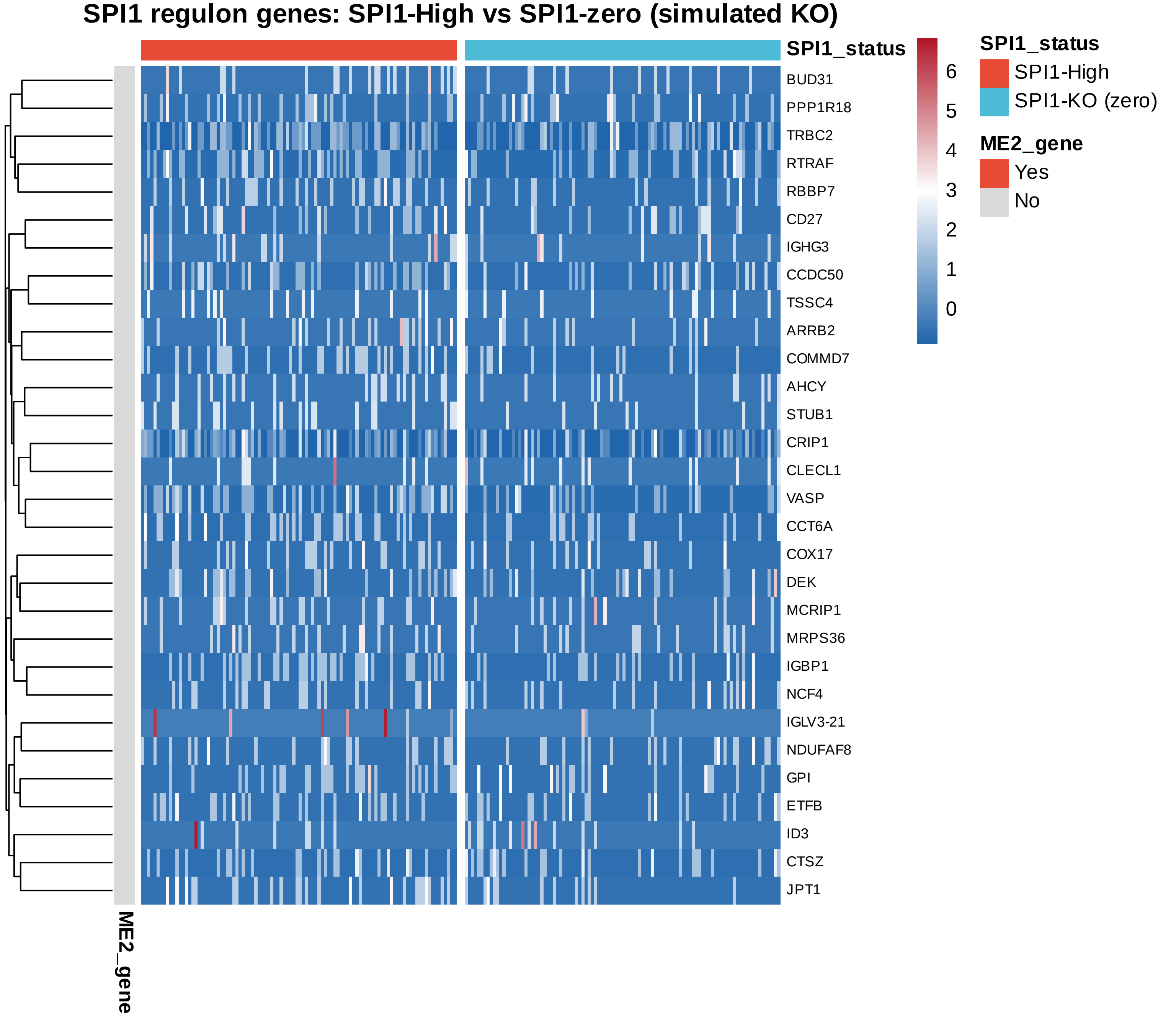
SPI1 regulon genes: SPI1-High vs SPI1-zero (simulated KO)
SPI1_status
BUD31
PPP1R18
TRBC2
RTRAF
RBBP7
CD27
IGHG3
CCDC50
TSSC4
ARRB2
COMMD7
AHCY
STUB1
CRIP1
CLECL1
VASP
CCT6A
COX17
DEK
MCRIP1
MRPS36
IGBP1
NCF4
IGLV3-21
NDUFAF8
GPI
ETFB
ID3
CTSZ
JPT1
ME2_gene
6
5
4
3
2
1
0
SPI1_status
SPI1-High
SPI1-KO (zero)
ME2_gene
Yes
No
