# Supplementary material for: Systems pharmacology reveals type I interferon and myeloid-like B cell reprogramming as candidate druggable axes in antiphospholipid syndrome
Source: PLoS One. 2026 Aug 26;21(8):e0349155. doi: 10.1371/journal.pone.0349155 (PMC13514046; doi:10.1371/journal.pone.0349155)

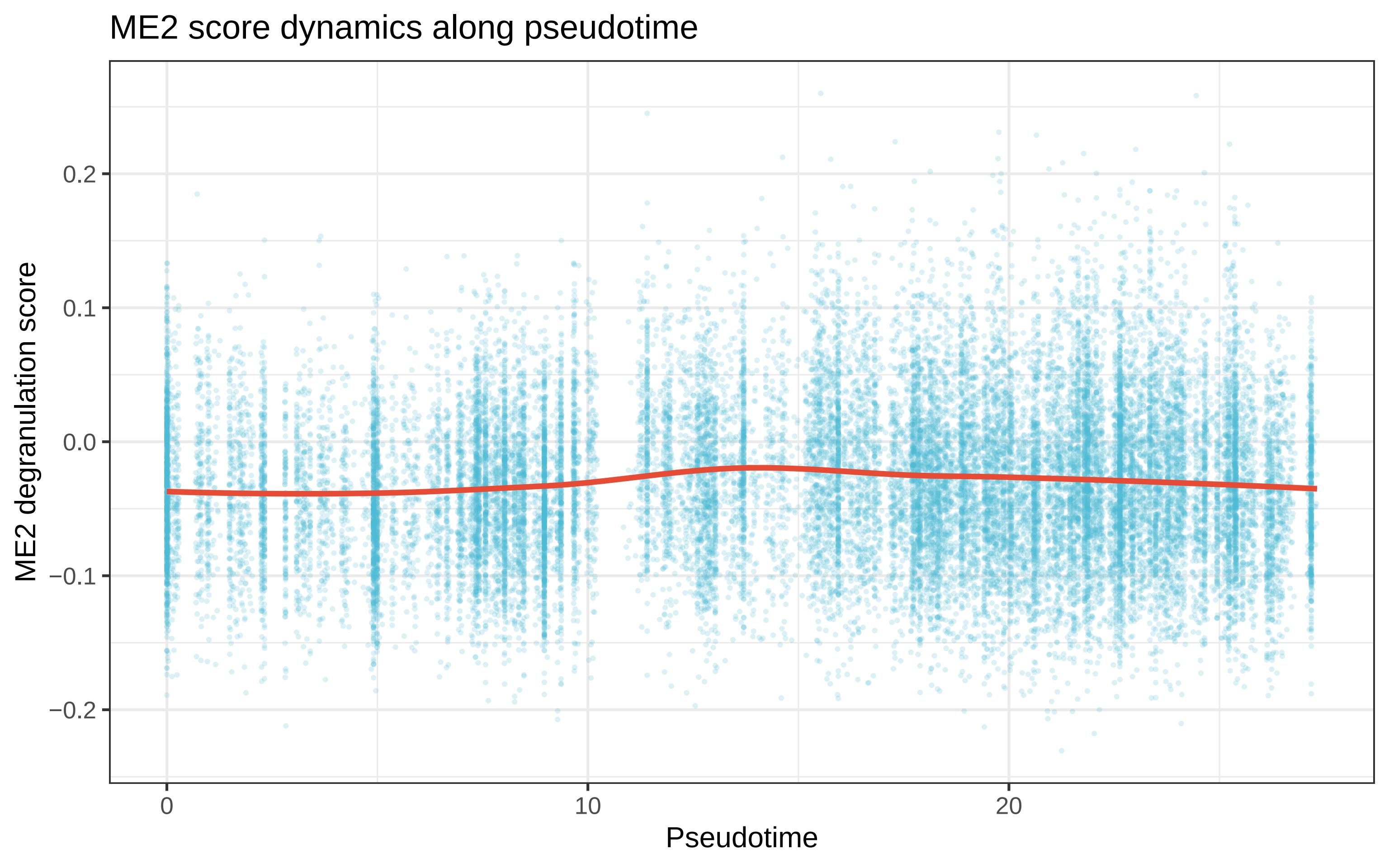

ME2 score dynamics along pseudotime
ME2 degranulation score
0.2
0.1
0.0
−0.1
−0.2
0
10
20
Pseudotime

Myeloid gene detection rate along pseudotime

Transitional B cells (n=1,095)

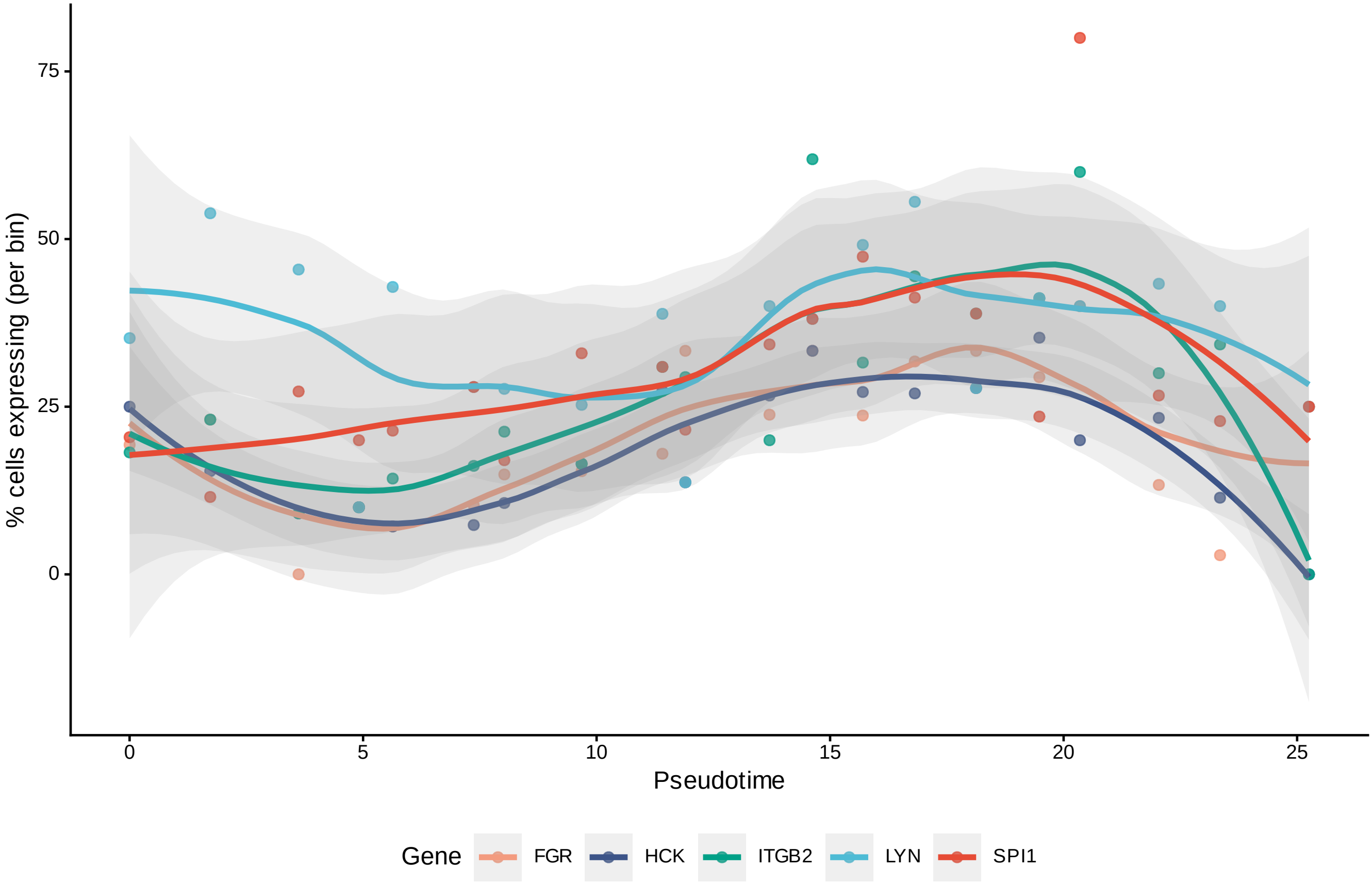

Supplement: Supplementary file 5 [file pone.0349155.s005.pdf]
