# Supplementary material for: Systems pharmacology reveals type I interferon and myeloid-like B cell reprogramming as candidate druggable axes in antiphospholipid syndrome
Source: PLoS One. 2026 Aug 26;21(8):e0349155. doi: 10.1371/journal.pone.0349155 (PMC13514046; doi:10.1371/journal.pone.0349155)
Supplement: Supplementary file 6 [file pone.0349155.s006.pdf]

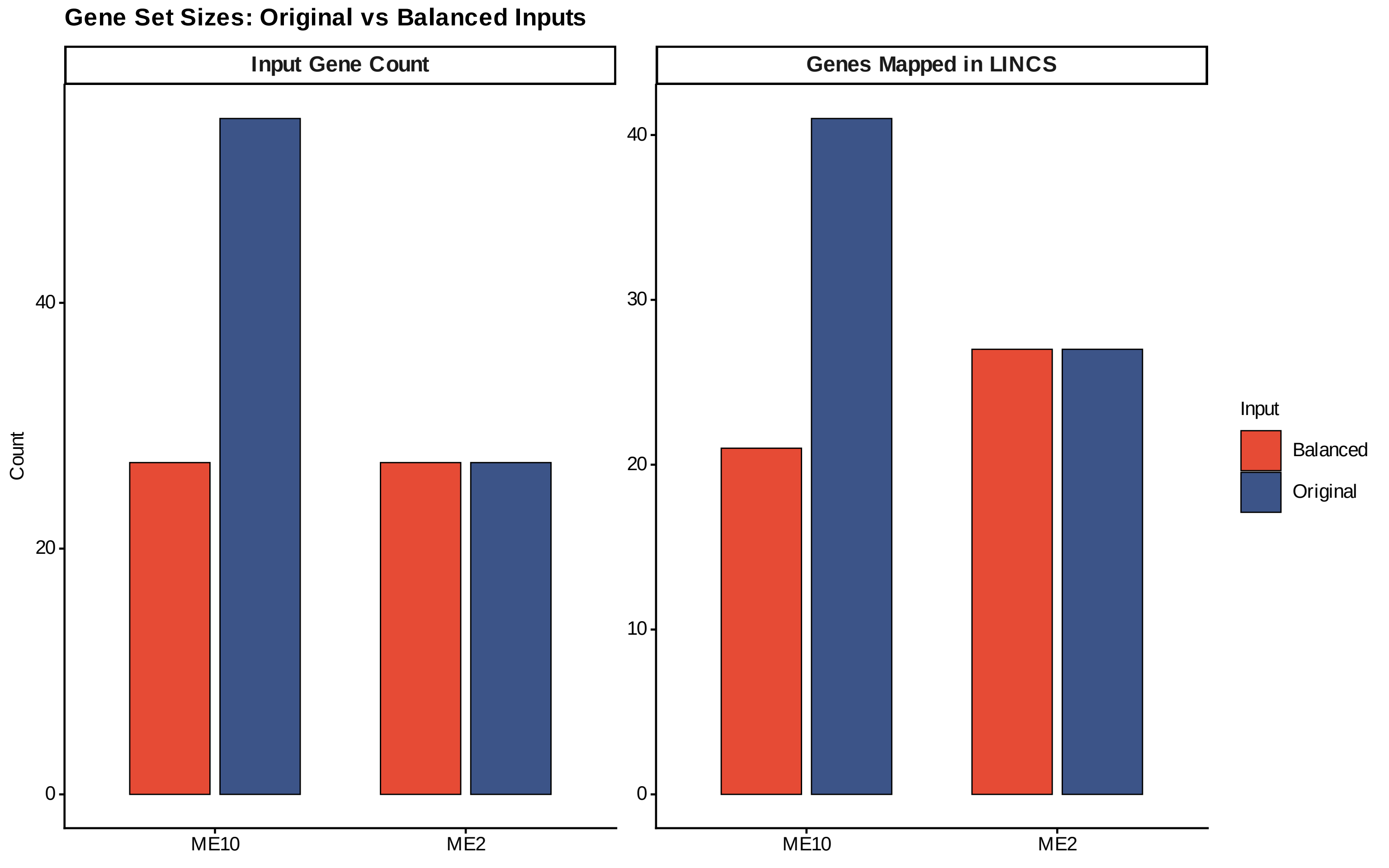
Gene Set Sizes: Original vs Balanced Inputs
Input Gene Count
Genes Mapped in LINCS
Count
0
20
40
0
10
20
30
40
ME10
ME2
ME10
ME2
Input
Balanced
Original

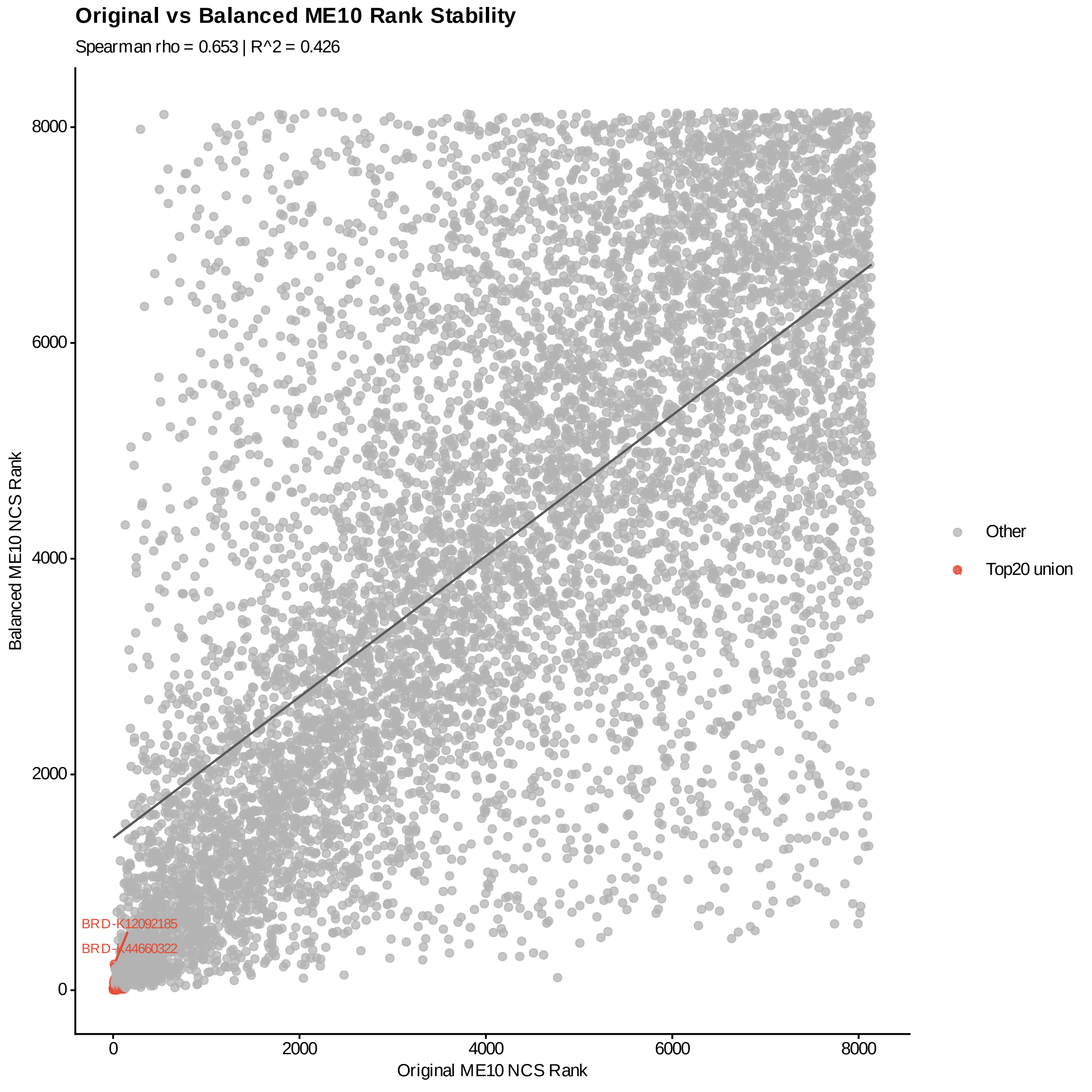

Original vs Balanced ME10 Rank Stability
Spearman rho = 0.653 | R^2 = 0.426
Balanced ME10 NCS Rank
Original ME10 NCS Rank
0
2000
4000
6000
8000
0
2000
4000
6000
8000
BRD-K12092185
BRD-K44660322
Other
Top20 union

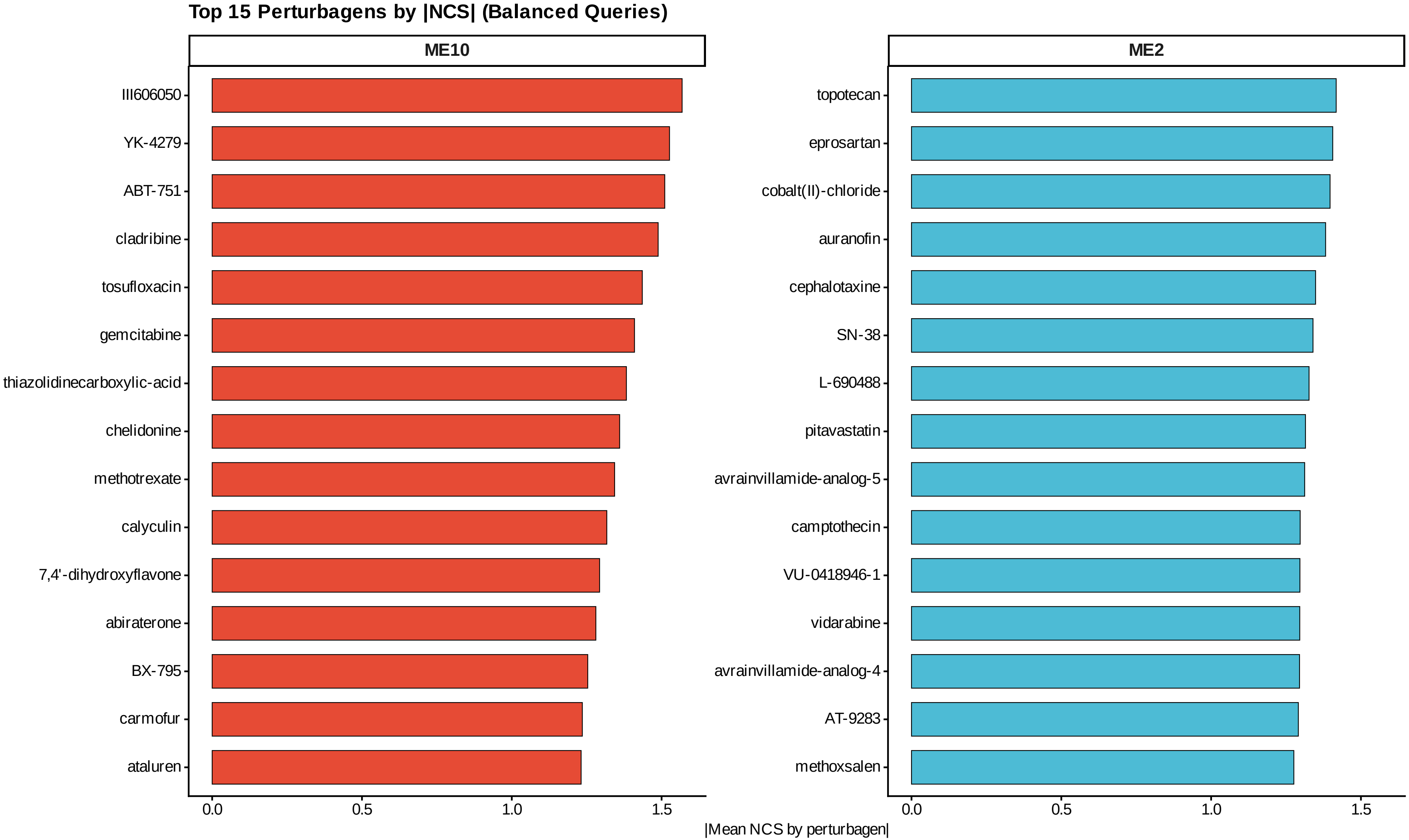

Top 15 Perturbagens by |NCS| (Balanced Queries)
ME10
III606050
YK-4279
ABT-751
cladribine
tosufloxacin
gemcitabine
thiazolidinecarboxylic-acid
chelidonine
methotrexate
calyculin
7,4'-dihydroxyflavone
abiraterone
BX-795
carmofur
ataluren
ME2
topotecan
eprosartan
cobalt(II)-chloride
auranofin
cephalotaxine
SN-38
L-690488
pitavastatin
avrainvillamide-analog-5
camptothecin
VU-0418946-1
vidarabine
avrainvillamide-analog-4
AT-9283
methoxsalen
0.0
0.5
1.0
1.5
|Mean NCS by perturbagen|
