# Supplementary material for: Systems pharmacology reveals type I interferon and myeloid-like B cell reprogramming as candidate druggable axes in antiphospholipid syndrome
Source: PLoS One. 2026 Aug 26;21(8):e0349155. doi: 10.1371/journal.pone.0349155 (PMC13514046; doi:10.1371/journal.pone.0349155)
Supplement: Supplementary file 7 [file pone.0349155.s007.pdf]

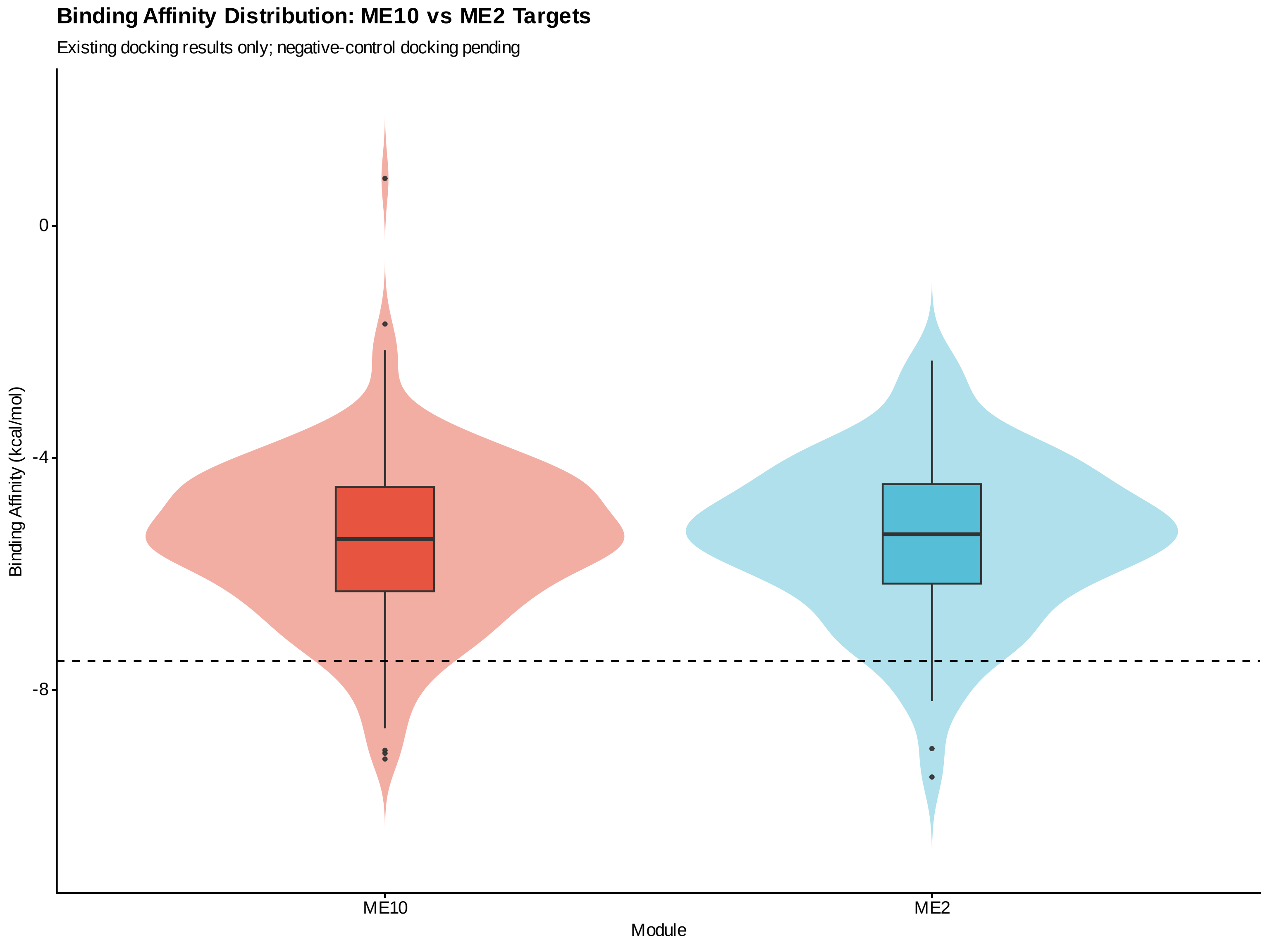
Binding Affinity Distribution: ME10 vs ME2 Targets
Existing docking results only; negative-control docking pending
0
-4
-8
Binding Affinity (kcal/mol)
ME10
ME2
Module

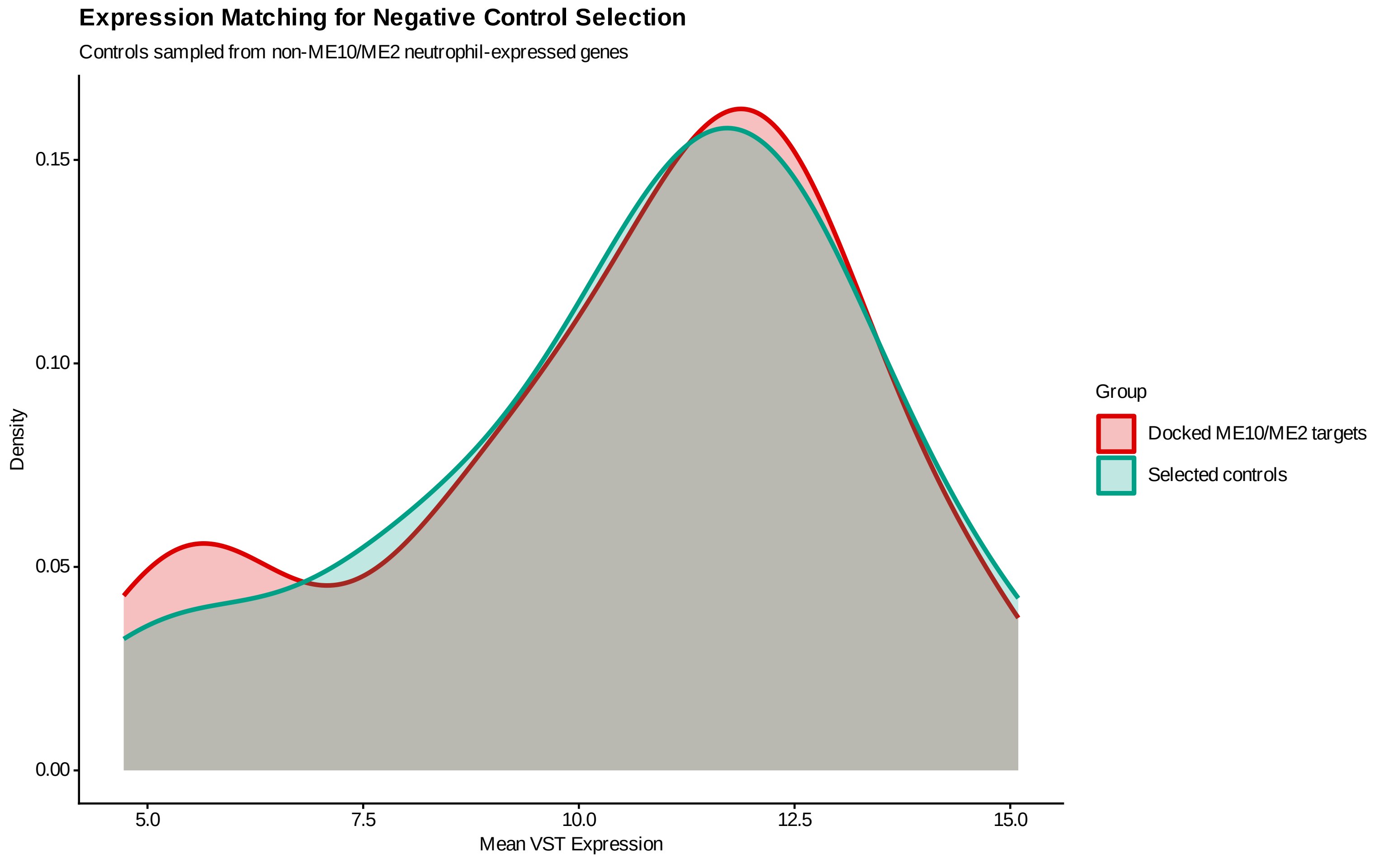

Expression Matching for Negative Control Selection
Controls sampled from non-ME10/ME2 neutrophil-expressed genes
Density
0.15
0.10
0.05
0.00
5.0
7.5
10.0
12.5
15.0
Mean VST Expression
Group
Docked ME10/ME2 targets
Selected controls

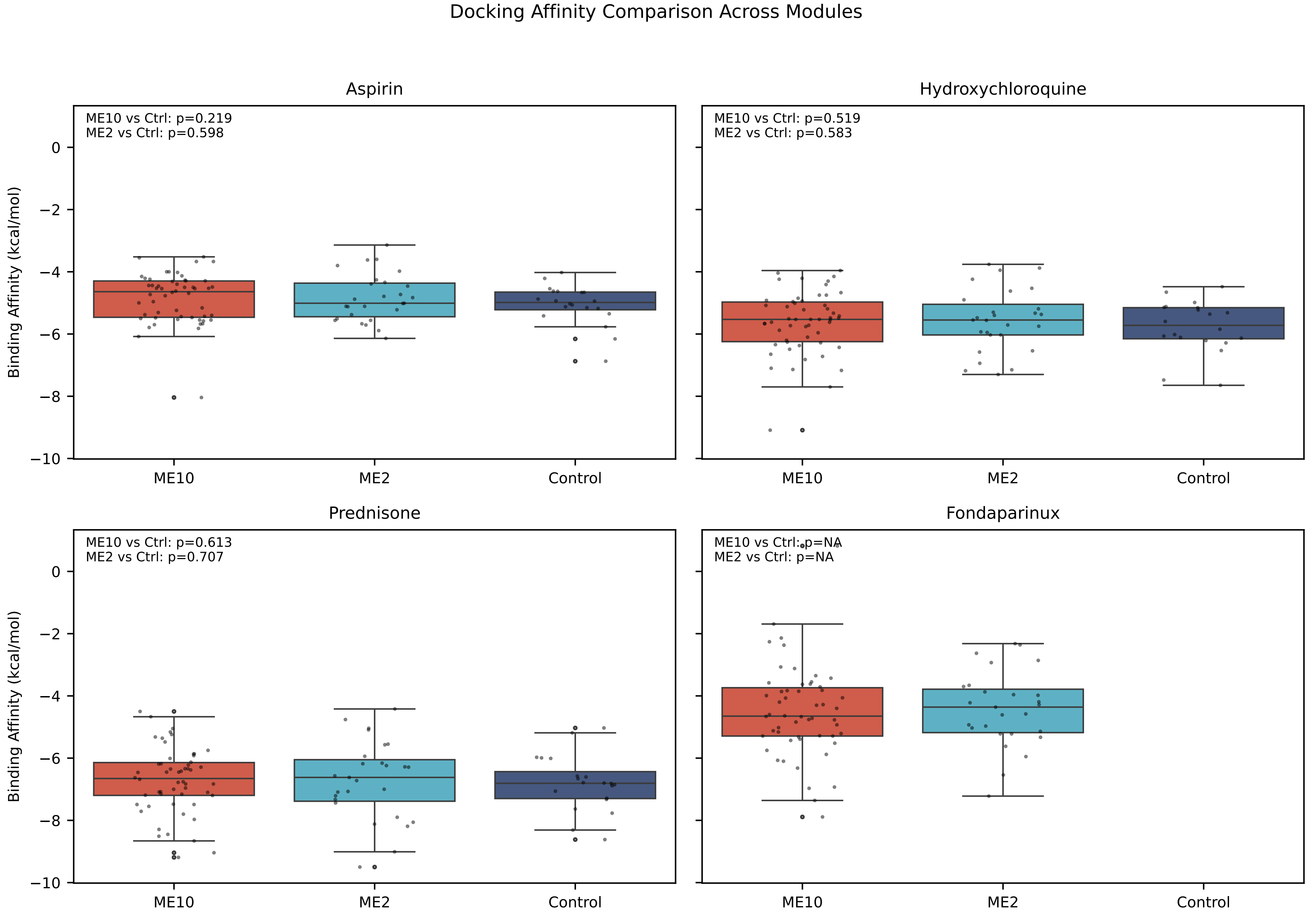
Docking Affinity Comparison Across Modules
Aspirin
ME10 vs Ctrl: p=0.219
ME2 vs Ctrl: p=0.598
Hydroxychloroquine
ME10 vs Ctrl: p=0.519
ME2 vs Ctrl: p=0.583
Prednisone
ME10 vs Ctrl: p=0.613
ME2 vs Ctrl: p=0.707
Fondaparinux
ME10 vs Ctrl: p=NA
ME2 vs Ctrl: p=NA
Binding Affinity (kcal/mol)
0
−2
−4
−6
−8
−10
ME10
ME2
Control

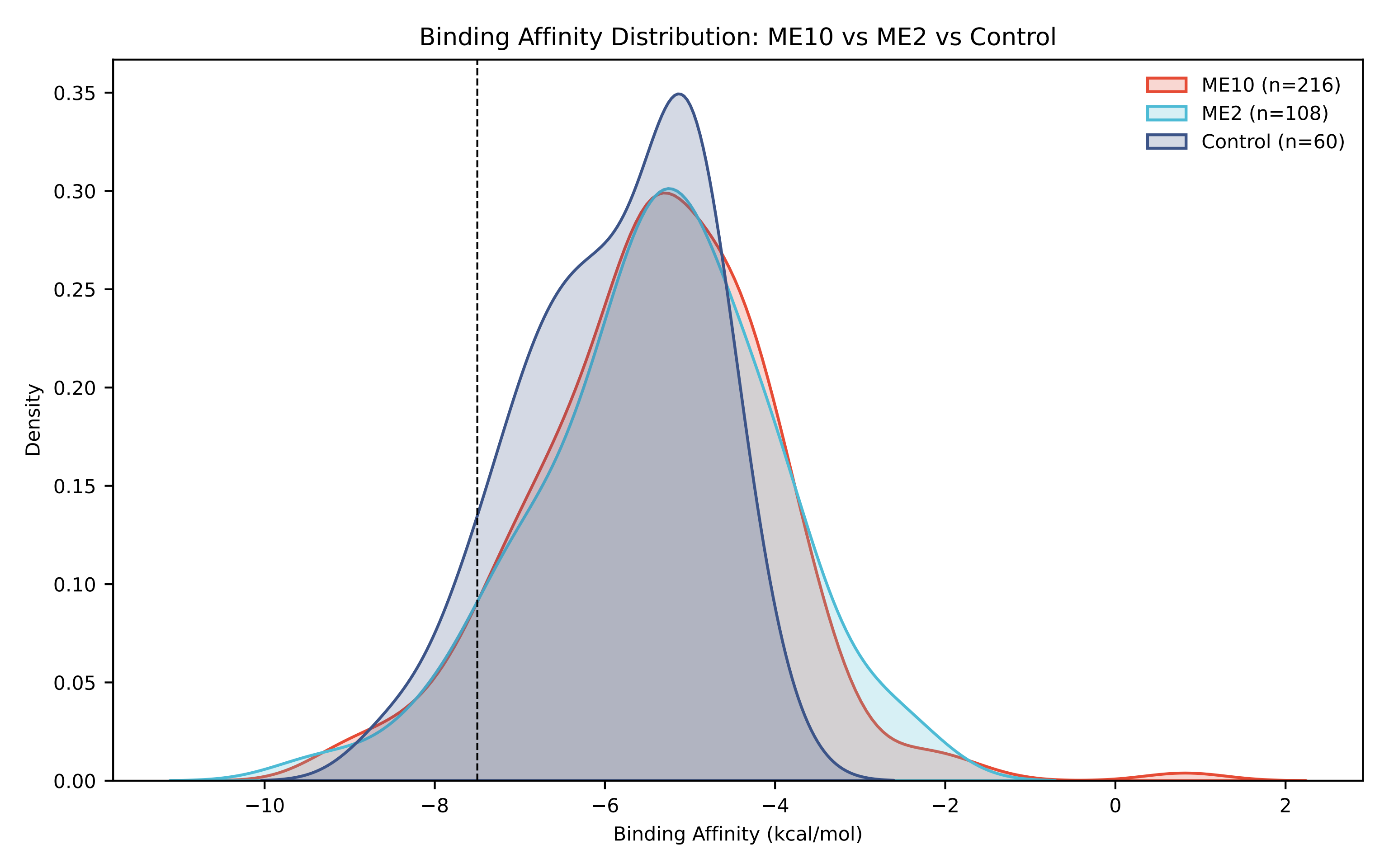

Binding Affinity Distribution: ME10 vs ME2 vs Control
ME10 (n=216)
ME2 (n=108)
Control (n=60)
Density
0.00
0.05
0.10
0.15
0.20
0.25
0.30
0.35
−10
−8
−6
−4
−2
0
2
Binding Affinity (kcal/mol)
