# Supplementary material for: Systems pharmacology reveals type I interferon and myeloid-like B cell reprogramming as candidate druggable axes in antiphospholipid syndrome
Source: PLoS One. 2026 Aug 26;21(8):e0349155. doi: 10.1371/journal.pone.0349155 (PMC13514046; doi:10.1371/journal.pone.0349155)

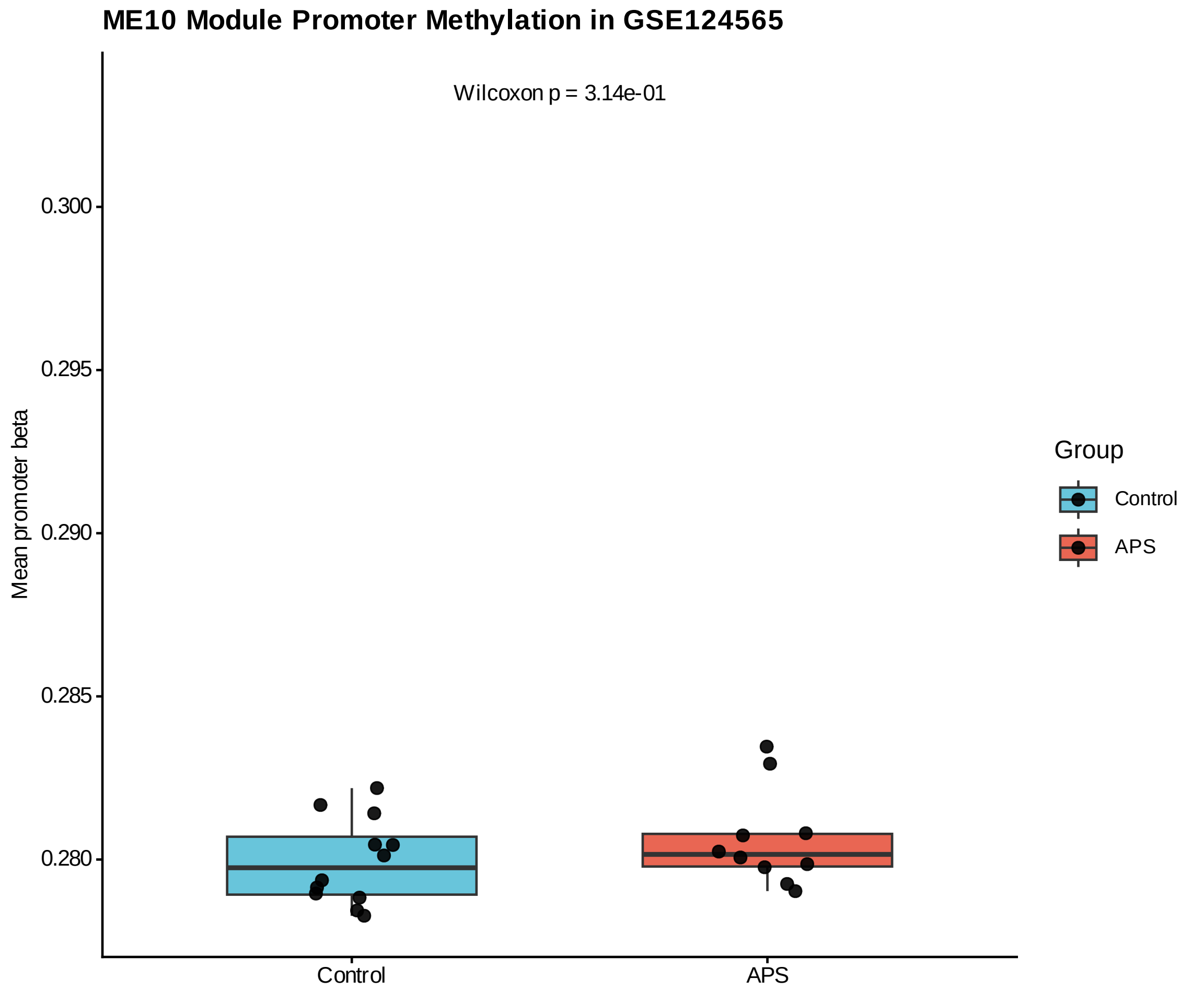
ME10 Module Promoter Methylation in GSE124565
Wilcoxon p = 3.14e-01
Mean promoter beta
0.300
0.295
0.290
0.285
0.280
Control
APS
Group
Control
APS

ME2 Module Promoter Methylation in GSE124565

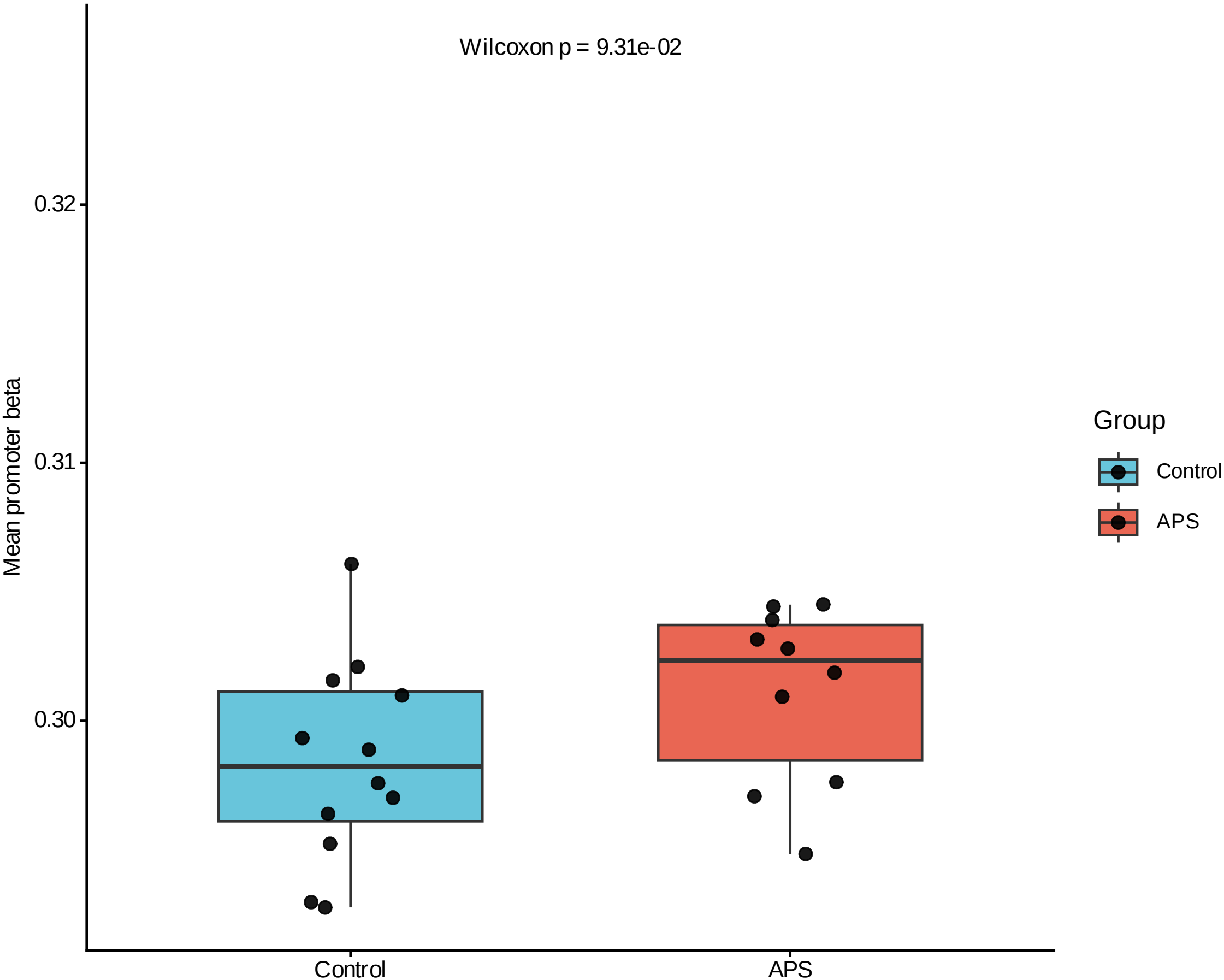

Supplement: Supplementary file 8 [file pone.0349155.s008.pdf]
