# Supplementary material for: Systems pharmacology reveals type I interferon and myeloid-like B cell reprogramming as candidate druggable axes in antiphospholipid syndrome
Source: PLoS One. 2026 Aug 26;21(8):e0349155. doi: 10.1371/journal.pone.0349155 (PMC13514046; doi:10.1371/journal.pone.0349155)
Supplement: Supplementary file 9 [file pone.0349155.s009.pdf]

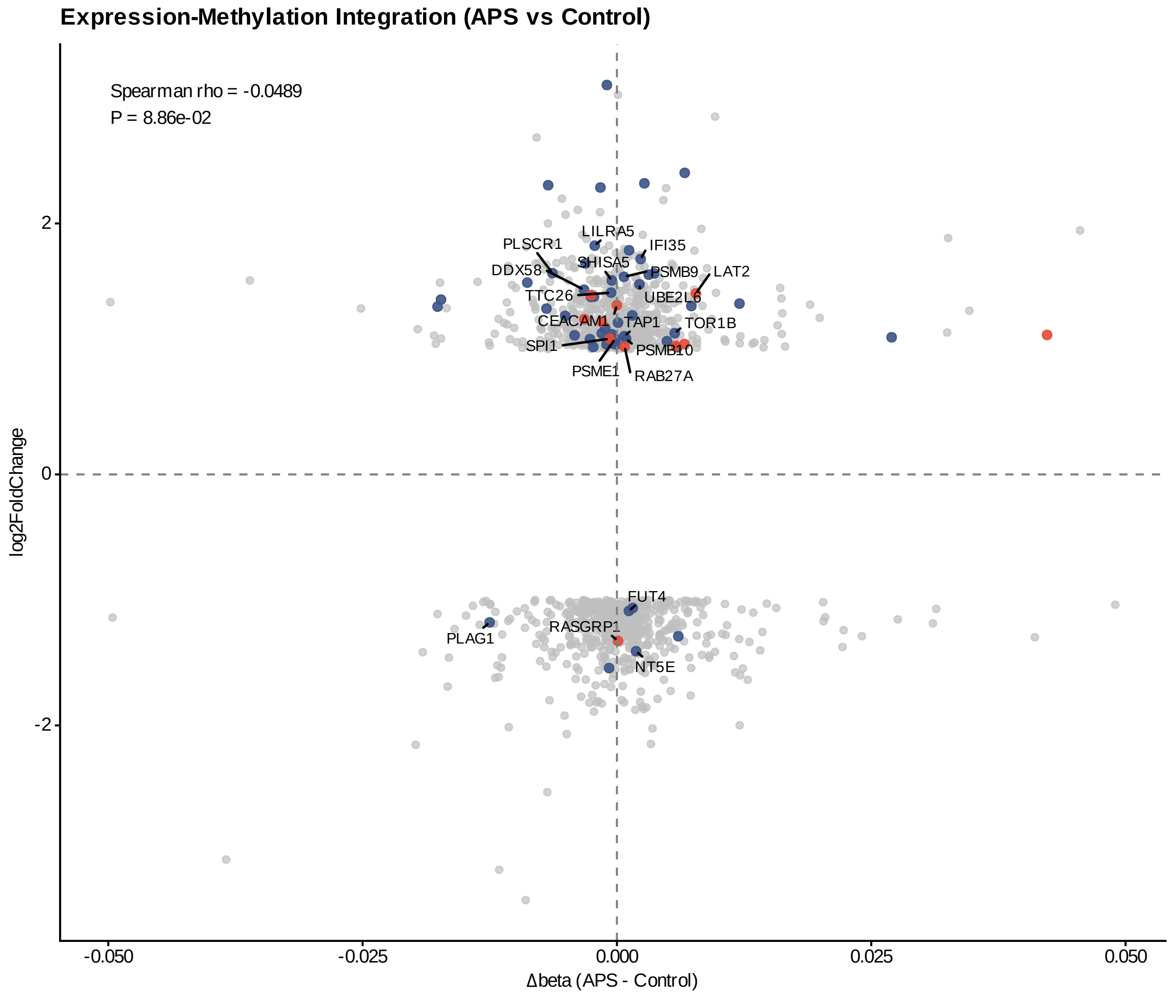

Expression-Methylation Integration (APS vs Control)
Spearman rho = -0.0489
P = 8.86e-02
log2FoldChange
Δbeta (APS - Control)
2
0
-2
-0.050
-0.025
0.000
0.025
0.050
LILRA5
PLSCR1
IFI35
SHISA5
DDX58
PSMB9
LAT2
TTC26
UBE2L6
CEACAM1
TAP1
TOR1B
SPI1
PSMB10
PSME1
RAB27A
FUT4
RASGRP1
PLAG1
NT5E

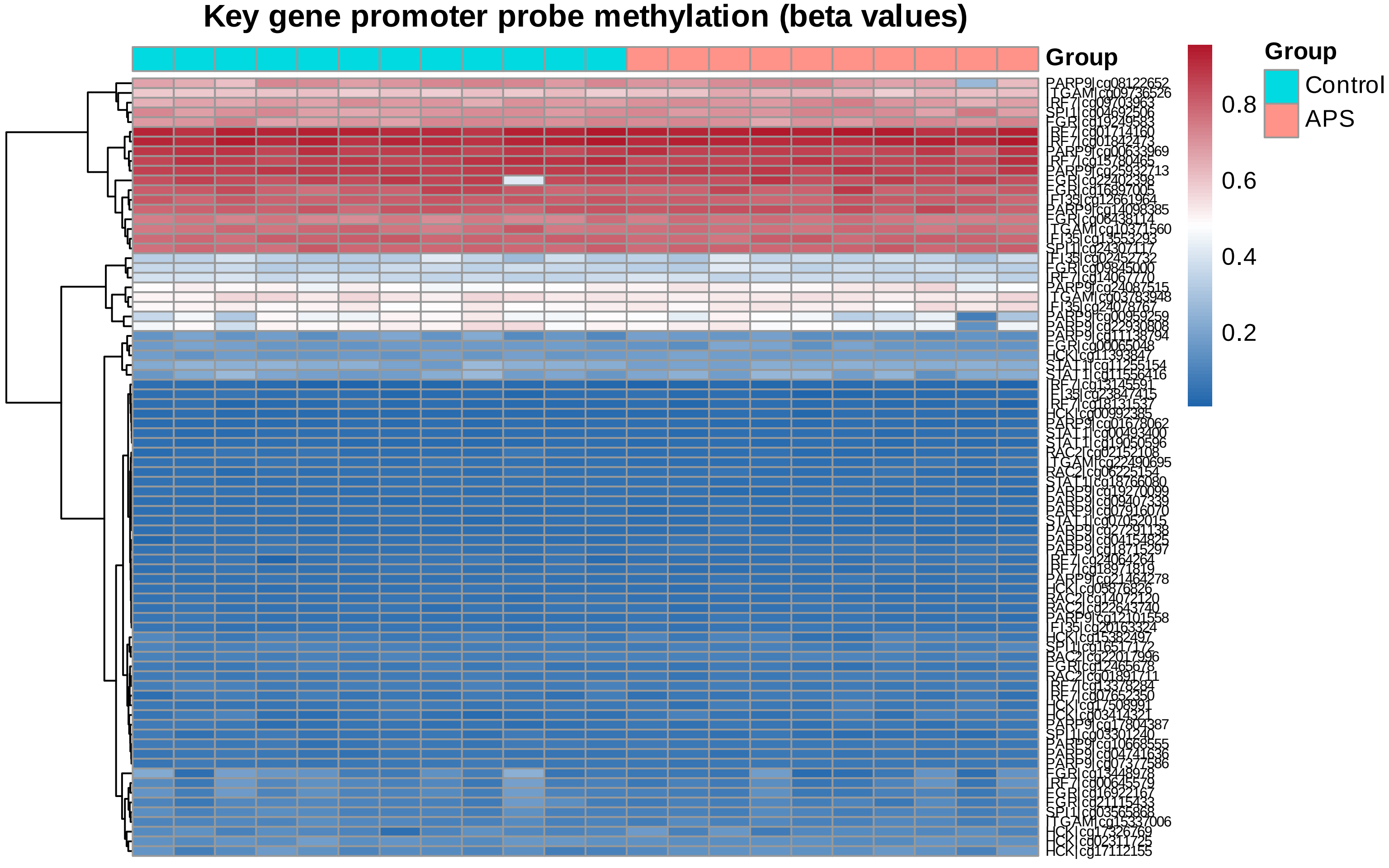

Key gene promoter probe methylation (beta values)
Group
Control
APS
0.8
0.6
0.4
0.2
PARP9|cg08122652
ITGAM|cg09736526
IRF7|cg09703963
SPI1|cg04692508
FGR|cg19249583
IRF7|cg01714160
IRF7|cg01842473
PARP9|cg00633969
IRF7|cg15780465
PARP9|cg25932713
FGR|cg22402398
FGR|cg16897005
IFI35|cg12661964
PARP9|cg14098385
FGR|cg06438114
ITGAM|cg10371560
IFI35|cg13553293
SPI1|cg24307117
IFI35|cg02452732
FGR|cg09845000
IRF7|cg14067770
PARP9|cg24087515
ITGAM|cg03783948
IFI35|cg24078767
PARP9|cg00959259
PARP9|cg22930808
PARP9|cg11138794
FGR|cg00065048
HCK|cg11393847
STAT1|cg11255154
STAT1|cg11556416
IRF7|cg13145591
IFI35|cg23847415
IRF7|cg18131537
HCK|cg00992385
PARP9|cg01678062
STAT1|cg00493400
STAT1|cg19050596
RAC2|cg02152108
ITGAM|cg22490695
RAC2|cg06225154
STAT1|cg18766080
PARP9|cg19270099
PARP9|cg09407339
PARP9|cg07916070
STAT1|cg07052015
PARP9|cg27291138
PARP9|cg04154825
PARP9|cg18715297
IRF7|cg24064264
IRF7|cg18971819
PARP9|cg21464278
HCK|cg05876826
RAC2|cg14072120
RAC2|cg22643740
PARP9|cg12101558
IFI35|cg20163324
HCK|cg15382497
SPI1|cg16517172
RAC2|cg22017996
FGR|cg12465678
RAC2|cg01891711
IRF7|cg13378284
IRF7|cg07652350
HCK|cg17508991
HCK|cg03414321
PARP9|cg17804387
SPI1|cg03301240
PARP9|cg10668555
PARP9|cg04741636
PARP9|cg07377586
FGR|cg13448978
IRF7|cg00645579
FGR|cg16922167
FGR|cg21115433
SPI1|cg03565868
ITGAM|cg15337006
HCK|cg17326769
HCK|cg02311725
HCK|cg17112155
