# Supplementary material for: Systems pharmacology reveals type I interferon and myeloid-like B cell reprogramming as candidate druggable axes in antiphospholipid syndrome
Source: PLoS One. 2026 Aug 26;21(8):e0349155. doi: 10.1371/journal.pone.0349155 (PMC13514046; doi:10.1371/journal.pone.0349155)
Supplement: Supplementary file 10 [file pone.0349155.s010.pdf]

Correlation of ME10/ME2 scores with immune infiltration

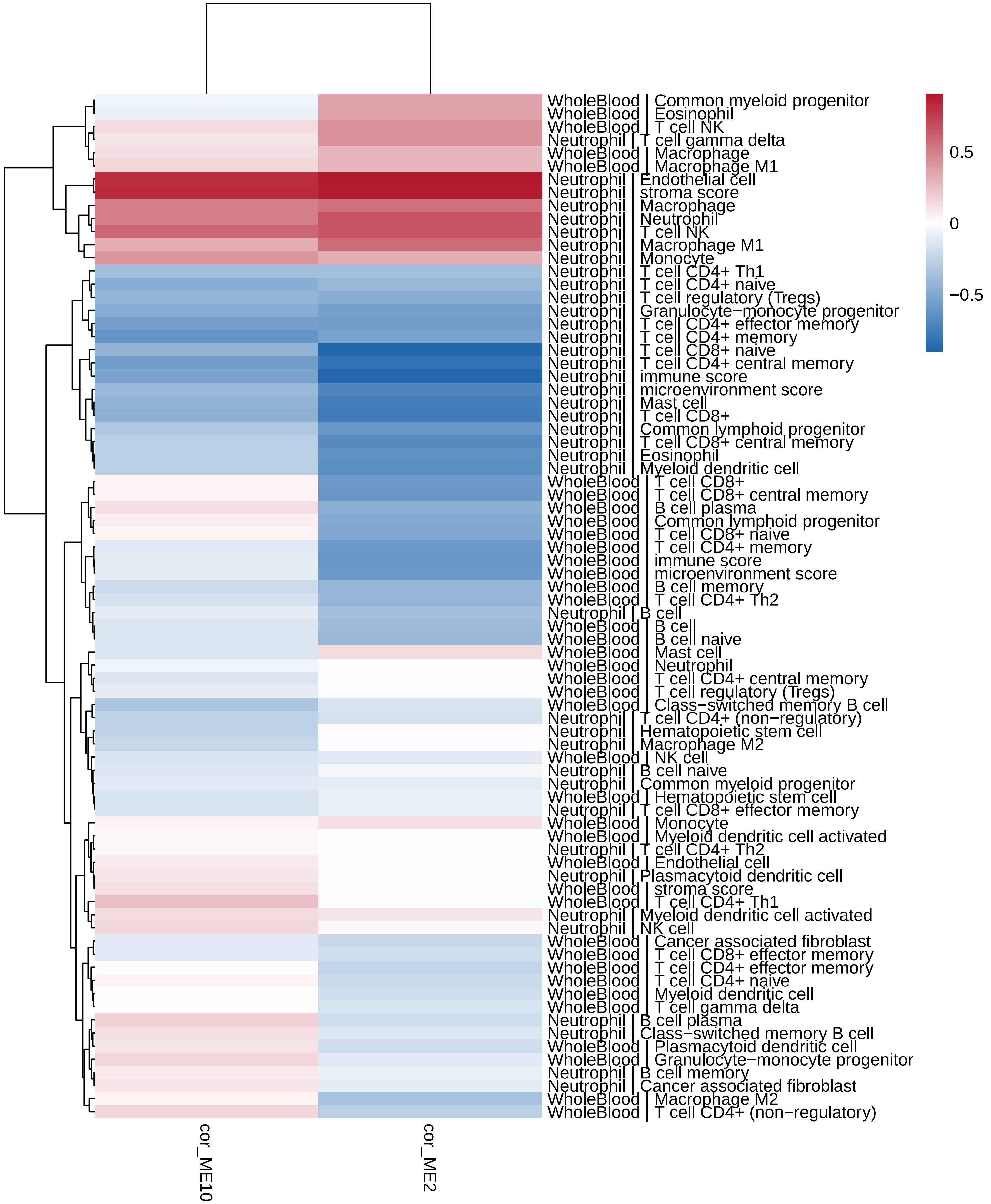

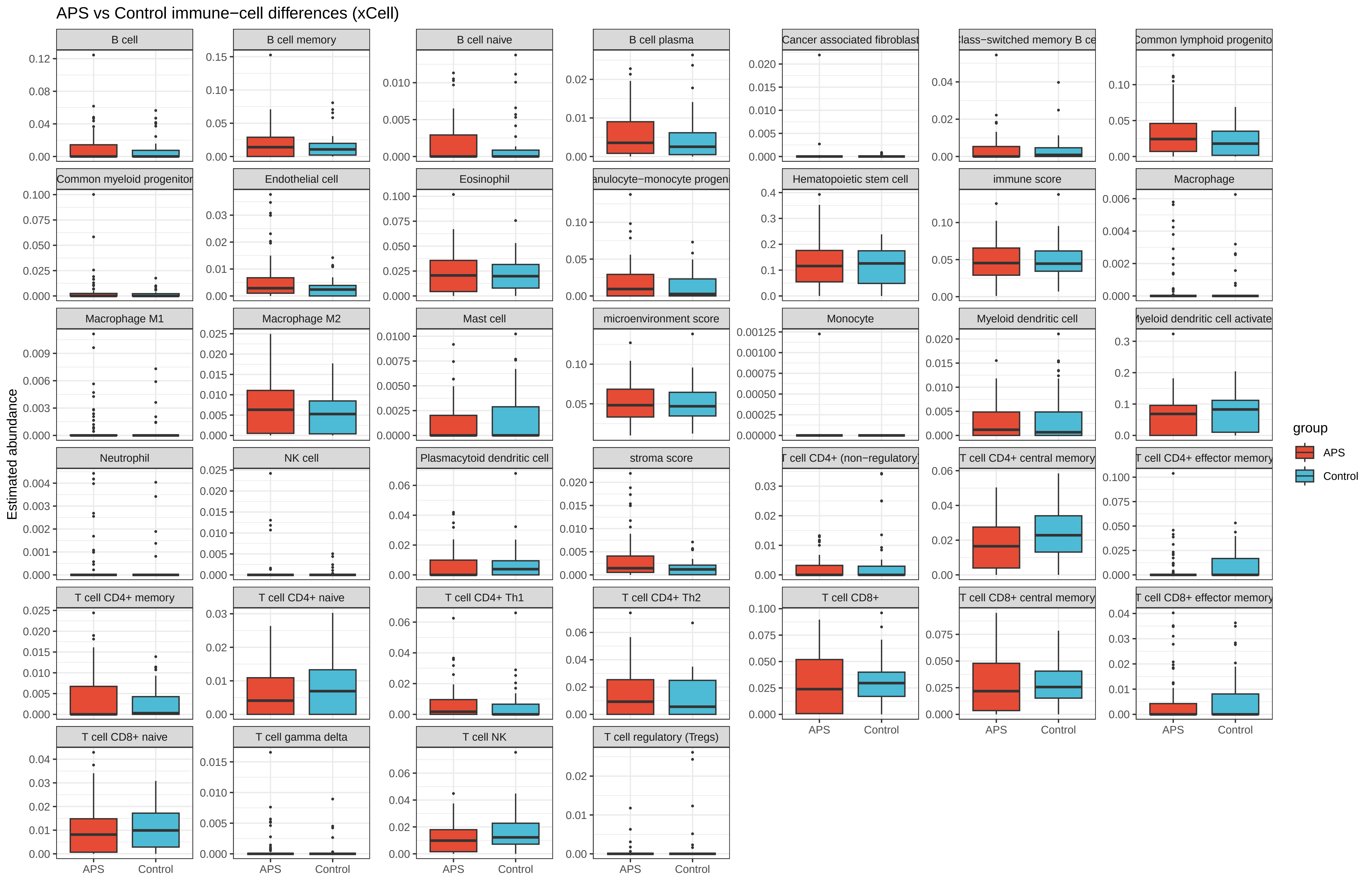

APS vs Control immune−cell differences (xCell)
Estimated abundance
B cell
B cell memory
B cell naive
B cell plasma
Cancer associated fibroblast
Class−switched memory B ce
Common lymphoid progenito
Common myeloid progenitor
Endothelial cell
Eosinophil
anulocyte−monocyte progen
Hematopoietic stem cell
immune score
Macrophage
Macrophage M1
Macrophage M2
Mast cell
microenvironment score
Monocyte
Myeloid dendritic cell
Myeloid dendritic cell activate
Neutrophil
NK cell
Plasmacytoid dendritic cell
stroma score
T cell CD4+ (non−regulatory)
T cell CD4+ central memory
T cell CD4+ effector memory
T cell CD4+ memory
T cell CD4+ naive
T cell CD4+ Th1
T cell CD4+ Th2
T cell CD8+
T cell CD8+ central memory
T cell CD8+ effector memory
T cell CD8+ naive
T cell gamma delta
T cell NK
T cell regulatory (Tregs)
APS
Control
group
APS
Control
