# Supplementary material for: Systems pharmacology reveals type I interferon and myeloid-like B cell reprogramming as candidate druggable axes in antiphospholipid syndrome
Source: PLoS One. 2026 Aug 26;21(8):e0349155. doi: 10.1371/journal.pone.0349155 (PMC13514046; doi:10.1371/journal.pone.0349155)
Supplement: Supplementary file 11 [file pone.0349155.s011.pdf]

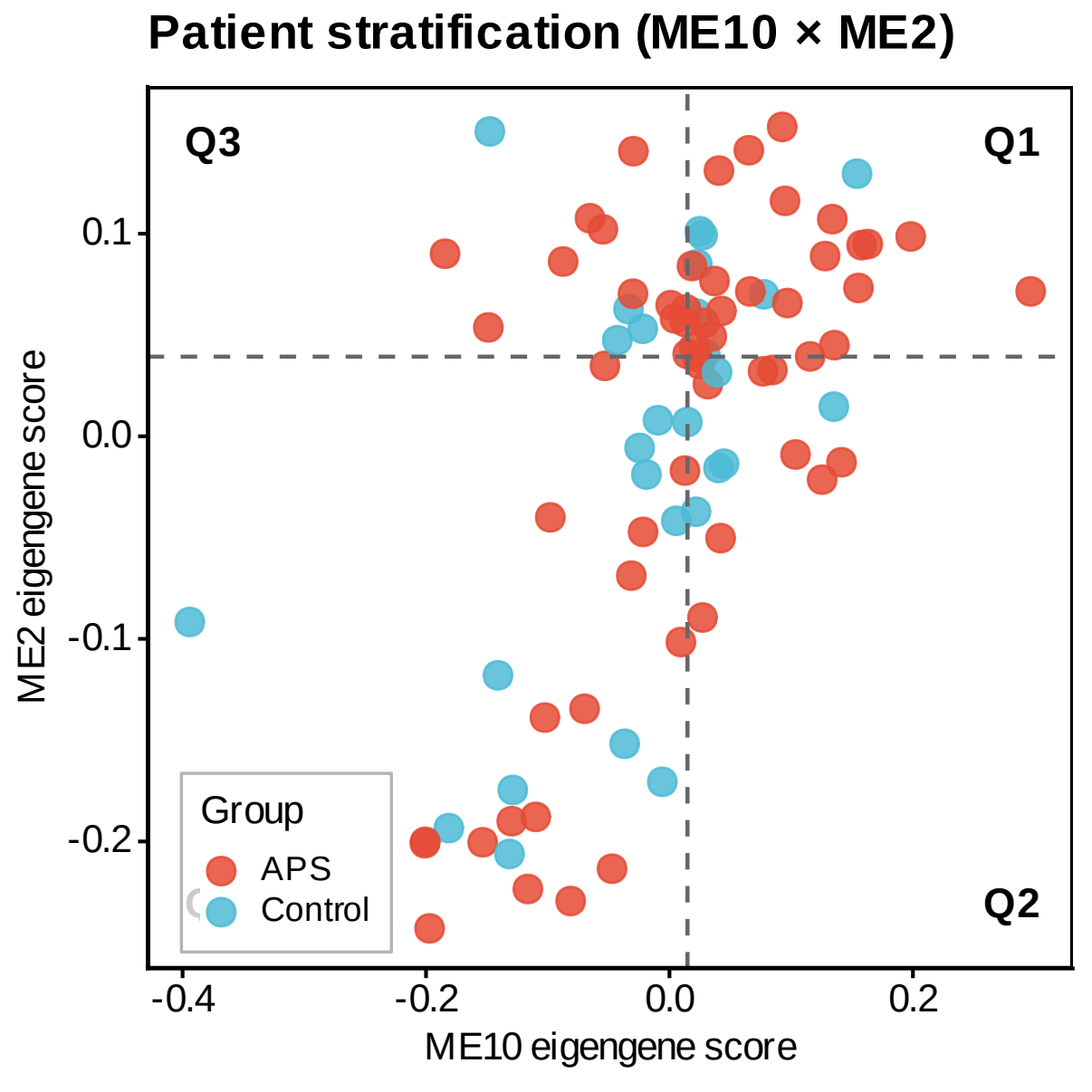

Patient stratification (ME10 × ME2)
Q3
Q1
Q2
Group
APS
Control
0.1
0.0
-0.1
-0.2
ME2 eigengene score
-0.4
-0.2
0.0
0.2
ME10 eigengene score

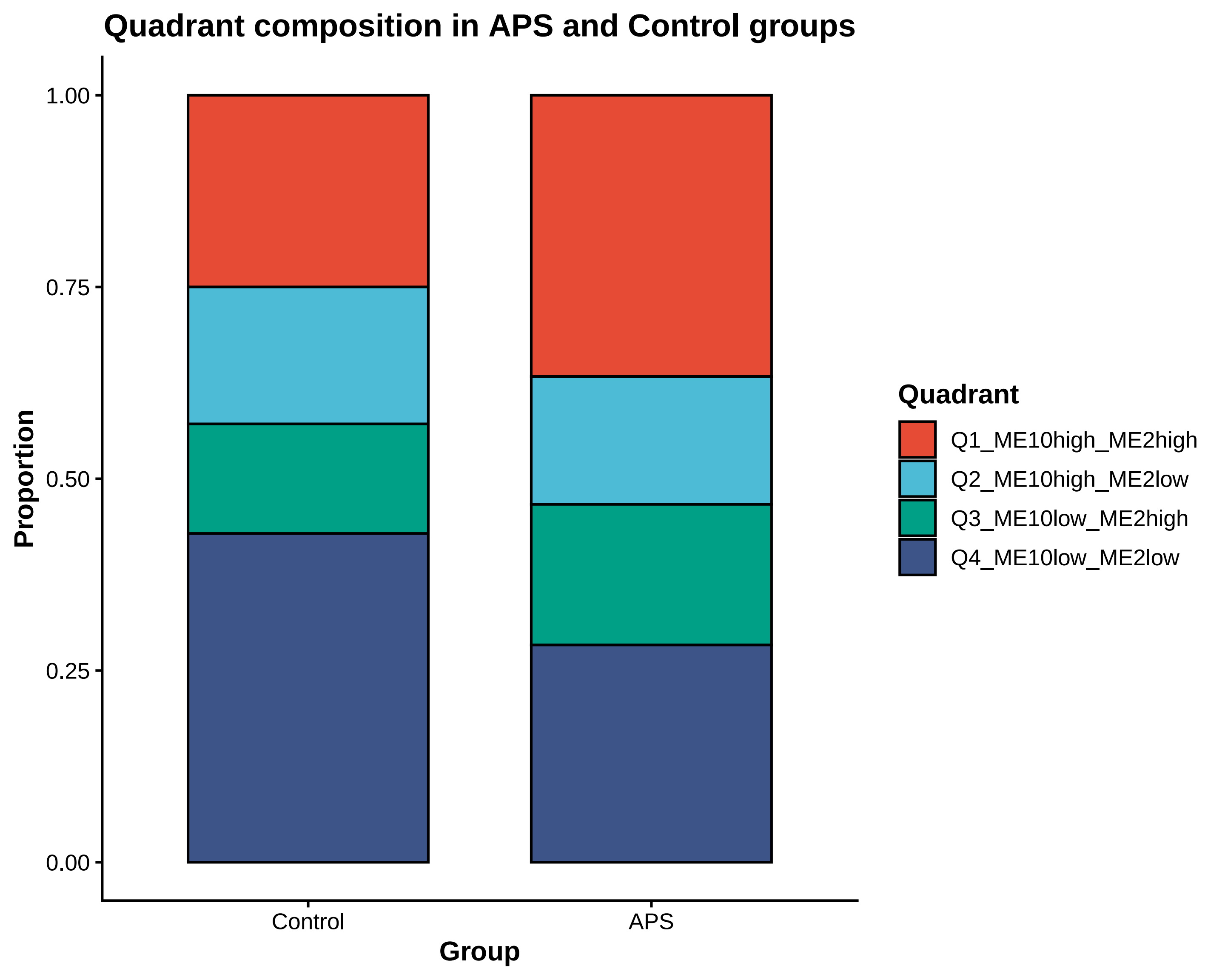
Quadrant composition in APS and Control groups
Proportion
1.00
0.75
0.50
0.25
0.00
Control
APS
Group
Quadrant
Q1_ME10high_ME2high
Q2_ME10high_ME2low
Q3_ME10low_ME2high
Q4_ME10low_ME2low
