# Supplementary material for: Systems pharmacology reveals type I interferon and myeloid-like B cell reprogramming as candidate druggable axes in antiphospholipid syndrome
Source: PLoS One. 2026 Aug 26;21(8):e0349155. doi: 10.1371/journal.pone.0349155 (PMC13514046; doi:10.1371/journal.pone.0349155)
Supplement: Supplementary file 12 [file pone.0349155.s012.pdf]

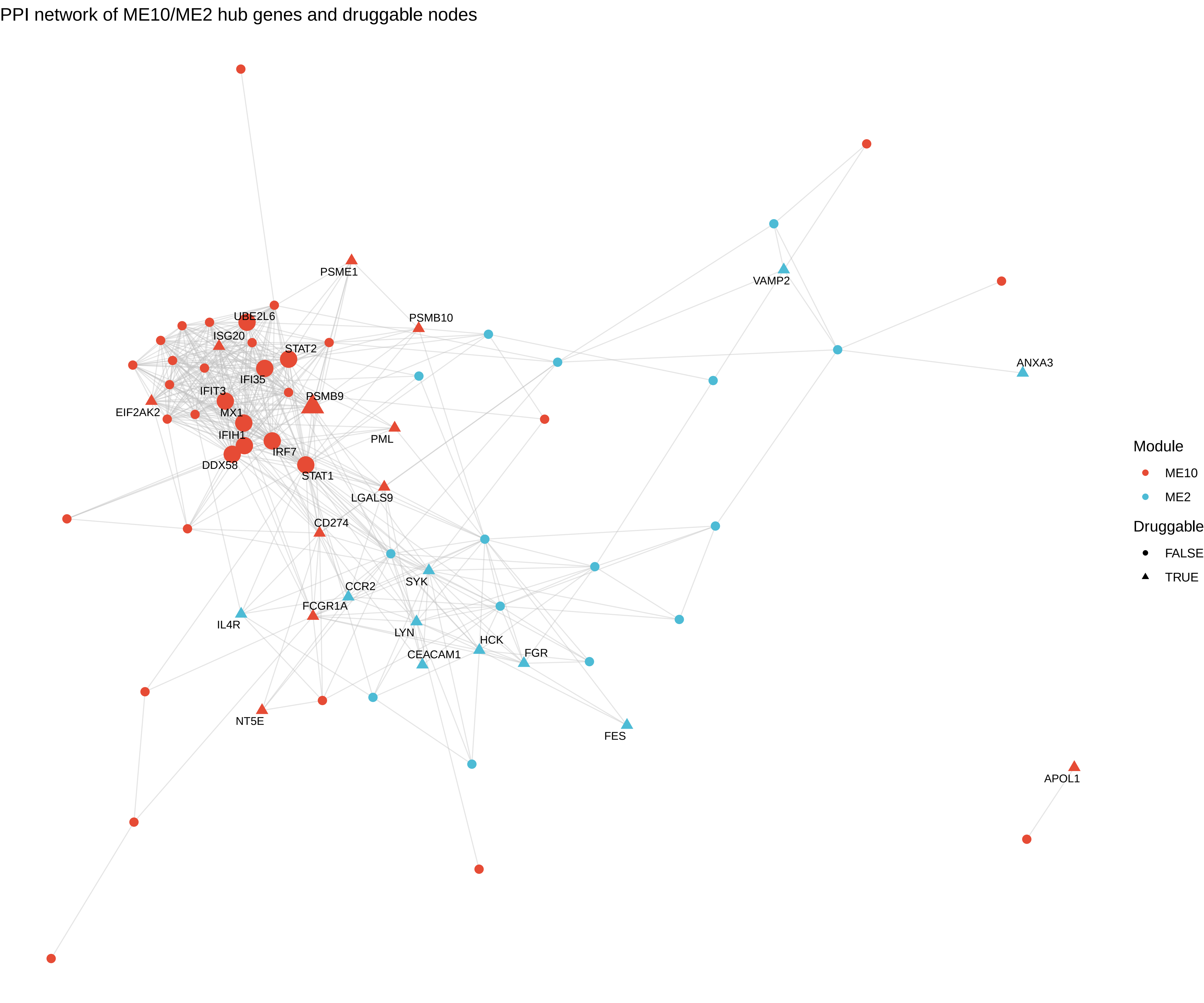

PPI network of ME10/ME2 hub genes and druggable nodes
PSME1
UBE2L6
PSMB10
ISG20
STAT2
IFI35
IFIT3
PSMB9
EIF2AK2
MX1
PML
IFIH1
IRF7
DDX58
STAT1
LGALS9
CD274
CCR2
SYK
FCGR1A
IL4R
LYN
HCK
CEACAM1
FGR
NT5E
FES
VAMP2
ANXA3
APOL1
Module
ME10
ME2
Druggable
FALSE
TRUE
