# Supplementary material for: Systems pharmacology reveals type I interferon and myeloid-like B cell reprogramming as candidate druggable axes in antiphospholipid syndrome
Source: PLoS One. 2026 Aug 26;21(8):e0349155. doi: 10.1371/journal.pone.0349155 (PMC13514046; doi:10.1371/journal.pone.0349155)
Supplement: Supplementary file 13 [file pone.0349155.s013.pdf]

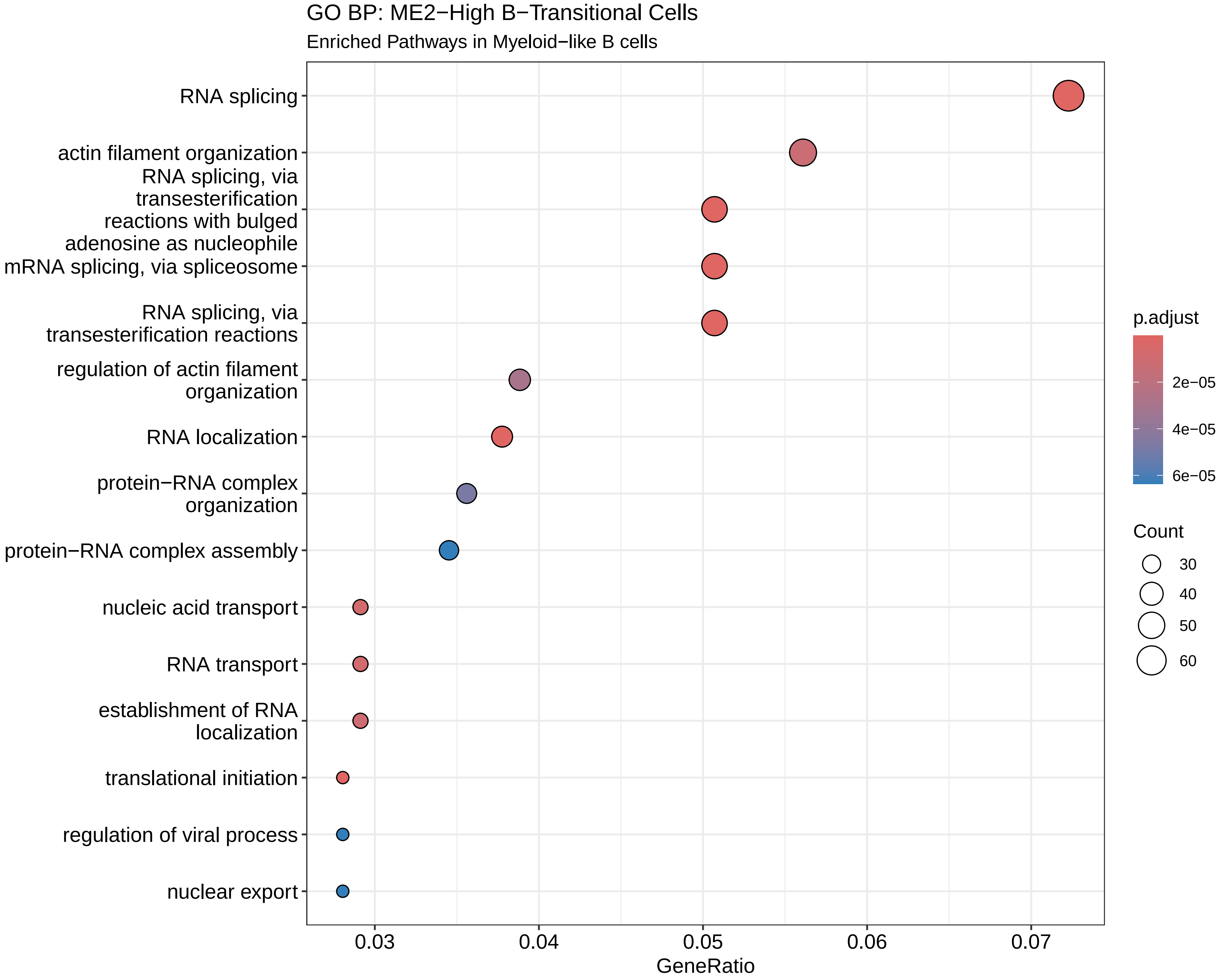
GO BP: ME2−High B−Transitional Cells
Enriched Pathways in Myeloid−like B cells
RNA splicing
actin filament organization
RNA splicing, via transesterification reactions with bulged adenosine as nucleophile
mRNA splicing, via spliceosome
RNA splicing, via transesterification reactions
regulation of actin filament organization
RNA localization
protein−RNA complex organization
protein−RNA complex assembly
nucleic acid transport
RNA transport
establishment of RNA localization
translational initiation
regulation of viral process
nuclear export
0.03
0.04
0.05
0.06
0.07
GeneRatio
p.adjust
2e−05
4e−05
6e−05
Count
30
40
50
60

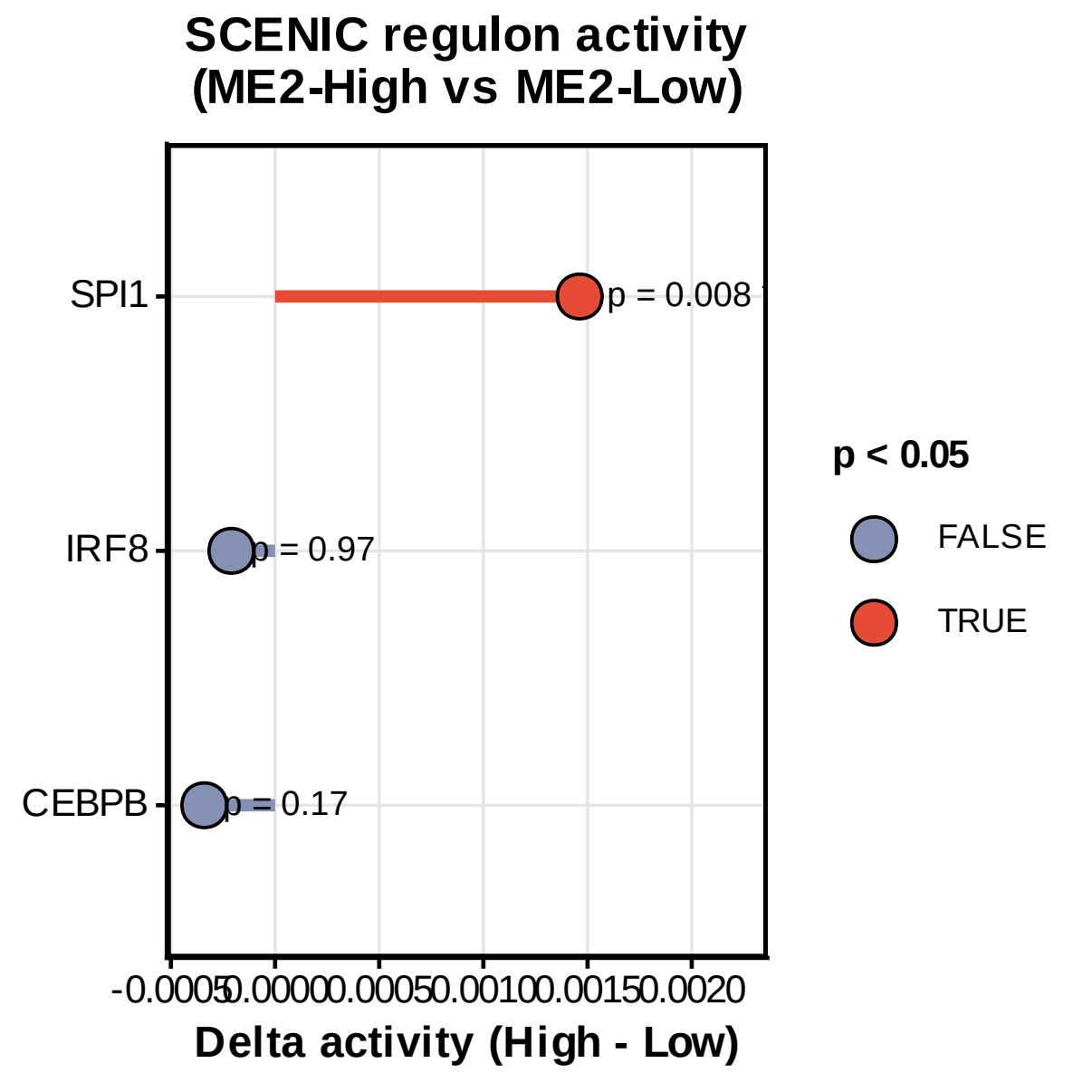
SCENIC regulon activity
(ME2-High vs ME2-Low)
SPI1
IRF8
CEBPB
p = 0.008
p = 0.97
p = 0.17
-0.0005
0.0000
0.0005
0.0010
0.0015
0.0020
Delta activity (High - Low)
p < 0.05
FALSE
TRUE
