# Supplementary material for: Systems pharmacology reveals type I interferon and myeloid-like B cell reprogramming as candidate druggable axes in antiphospholipid syndrome
Source: PLoS One. 2026 Aug 26;21(8):e0349155. doi: 10.1371/journal.pone.0349155 (PMC13514046; doi:10.1371/journal.pone.0349155)
Supplement: Supplementary file 14 [file pone.0349155.s014.pdf]

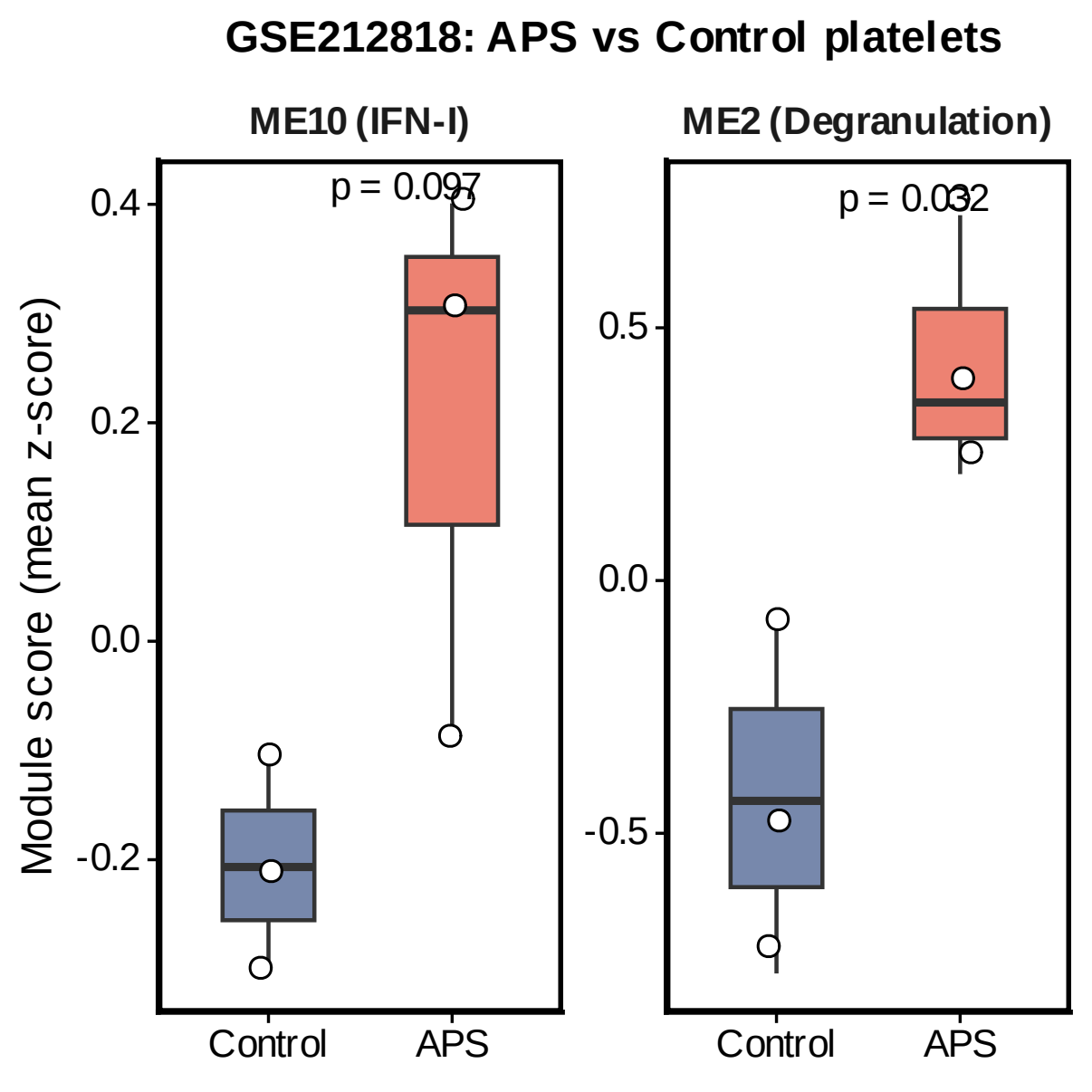
GSE212818: APS vs Control platelets
ME10 (IFN-I)
ME2 (Degranulation)
Module score (mean z-score)
p = 0.097
p = 0.032
0.4
0.2
0.0
-0.2
0.5
0.0
-0.5
Control
APS
Control
APS

GSE212818: Module genes in platelets

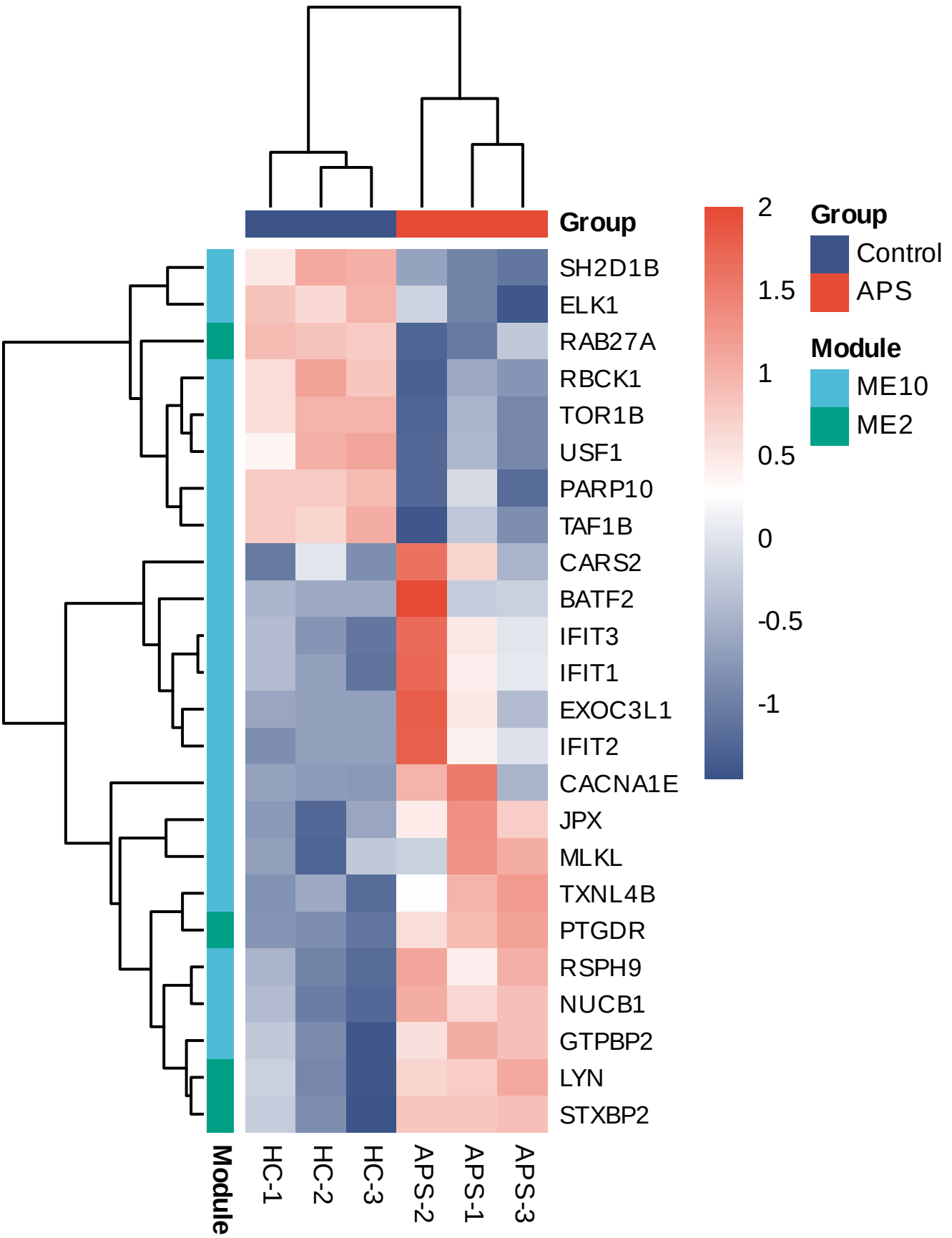

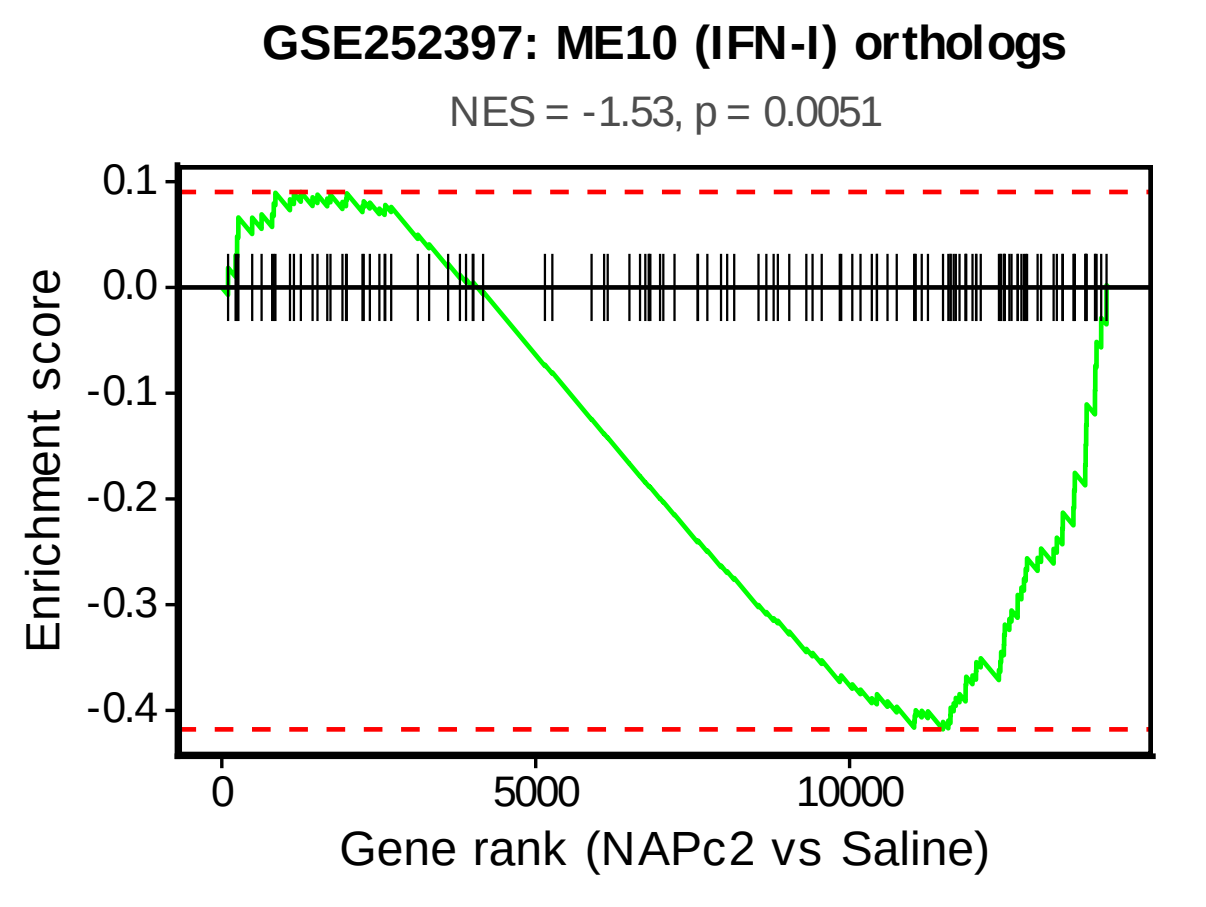
GSE252397: ME10 (IFN-I) orthologs
NES = -1.53, p = 0.0051
Enrichment score
0.1
0.0
-0.1
-0.2
-0.3
-0.4
0
5000
10000
Gene rank (NAPc2 vs Saline)

GSE252397: ME2 (Degranulation) orthologs

NES = -1.32, p = 0.1164

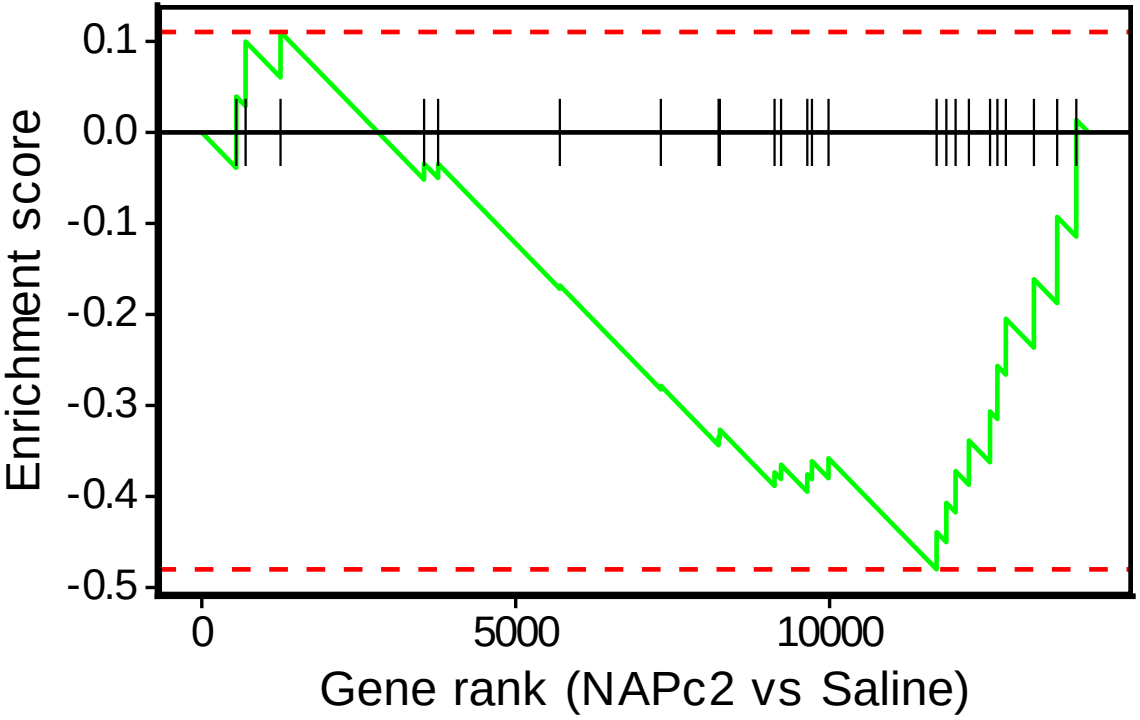

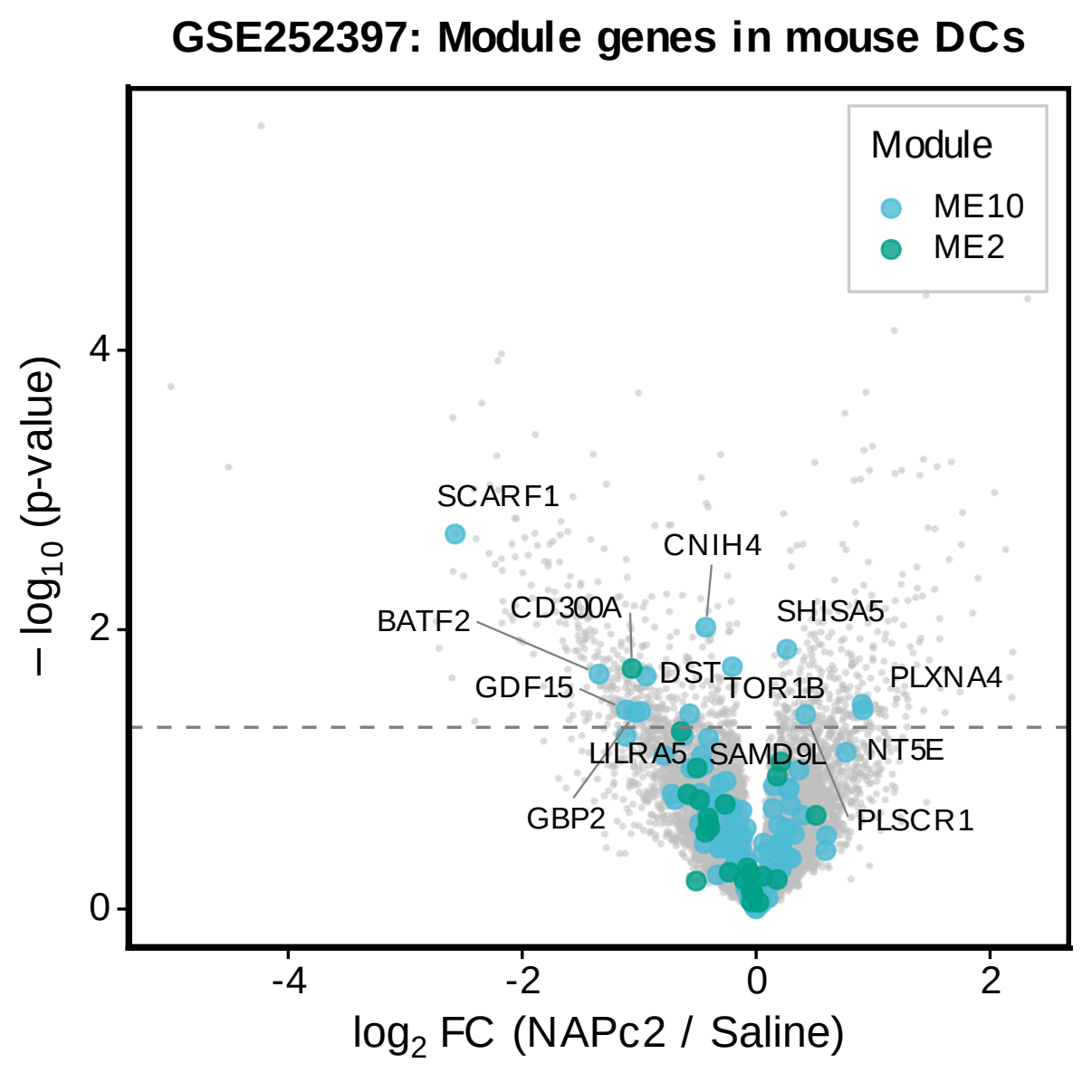

GSE252397: Module genes in mouse DCs
Module
ME10
ME2
SCARF1
CNIH4
CD300A
BATF2
SHISA5
DST
TOR1B
PLXNA4
GDF15
LILRA5
SAMD9L
NT5E
GBP2
PLSCR1
4
2
0
-4
-2
0
2
− log10 (p-value)
log2 FC (NAPc2 / Saline)
