# Supplementary material for: Systems pharmacology reveals type I interferon and myeloid-like B cell reprogramming as candidate druggable axes in antiphospholipid syndrome
Source: PLoS One. 2026 Aug 26;21(8):e0349155. doi: 10.1371/journal.pone.0349155 (PMC13514046; doi:10.1371/journal.pone.0349155)
Supplement: Supplementary file 15 [file pone.0349155.s015.pdf]

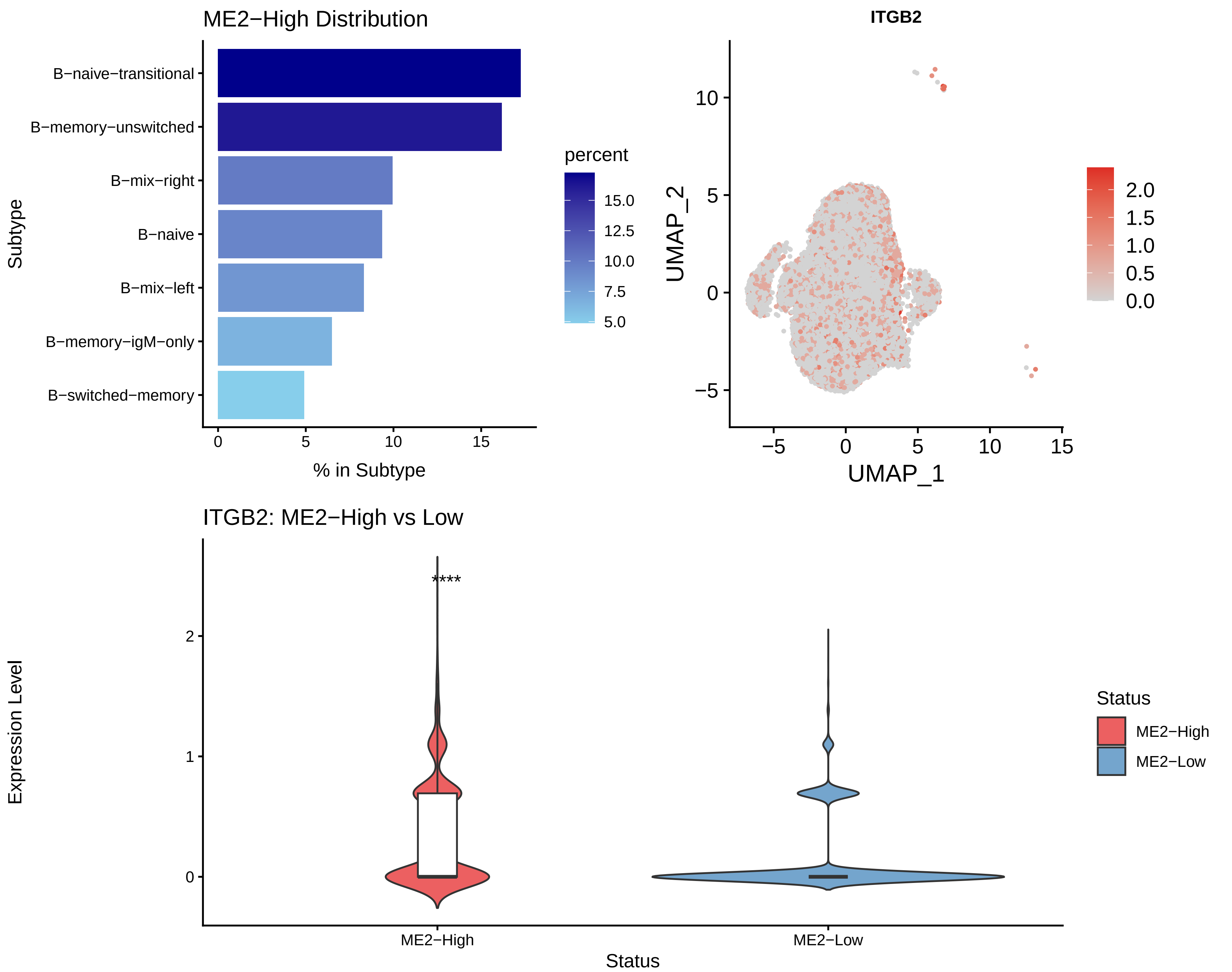
ME2−High Distribution
B−naive−transitional
B−memory−unswitched
B−mix−right
B−naive
B−mix−left
B−memory−igM−only
B−switched−memory
Subtype
0
5
10
15
% in Subtype
percent
15.0
12.5
10.0
7.5
5.0
ITGB2
10
5
0
−5
UMAP_2
−5
0
5
10
15
UMAP_1
2.0
1.5
1.0
0.5
0.0
ITGB2: ME2−High vs Low
****
2
1
0
Expression Level
ME2−High
ME2−Low
Status
Status
ME2−High
ME2−Low
