# Supplementary material for: Systems pharmacology reveals type I interferon and myeloid-like B cell reprogramming as candidate druggable axes in antiphospholipid syndrome
Source: PLoS One. 2026 Aug 26;21(8):e0349155. doi: 10.1371/journal.pone.0349155 (PMC13514046; doi:10.1371/journal.pone.0349155)
Supplement: Supplementary file 16 [file pone.0349155.s016.pdf]

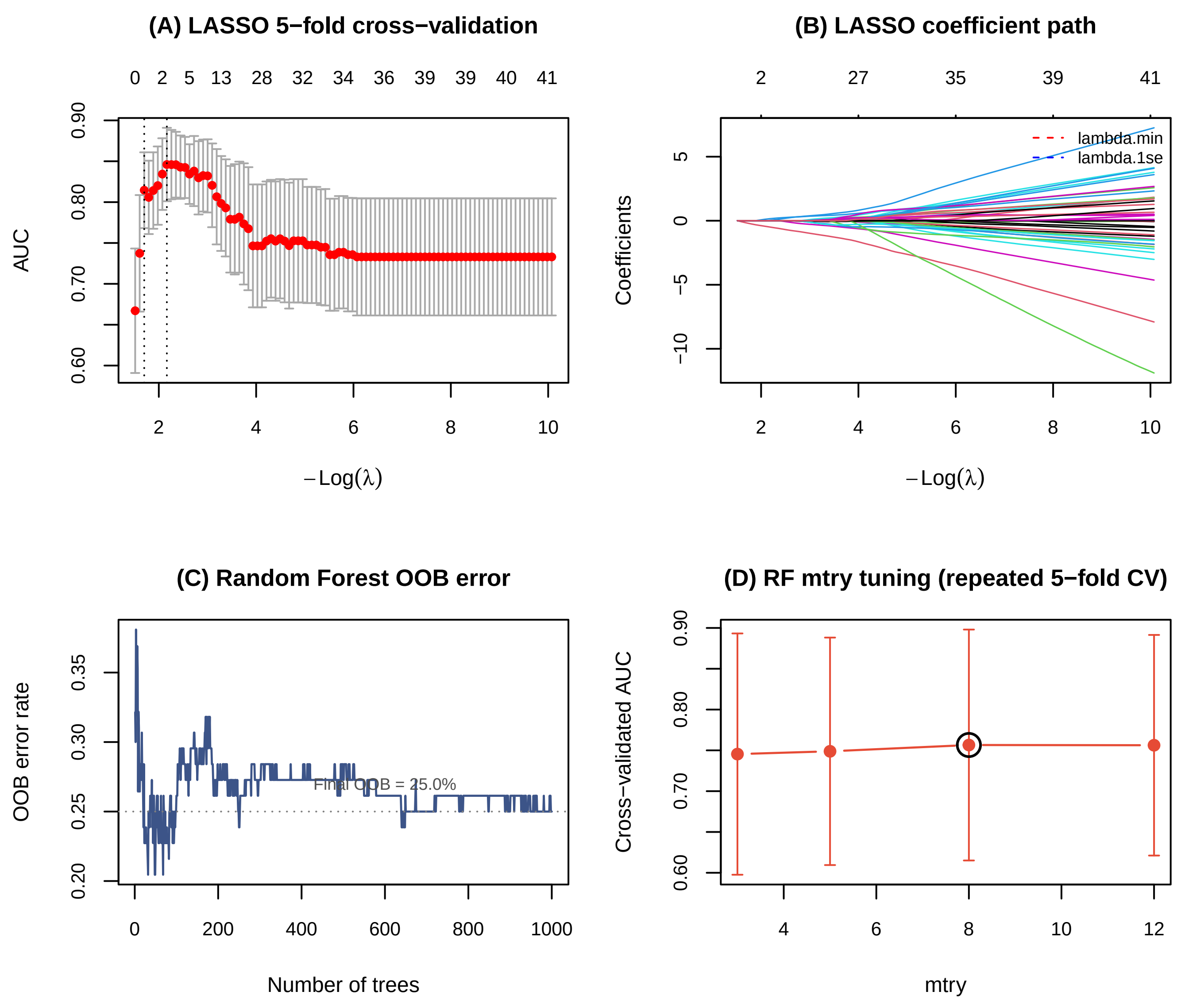

(A) LASSO 5−fold cross−validation
0 2 5 13 28 32 34 36 39 39 40 41
AUC
0.60 0.70 0.80 0.90
2 4 6 8 10
−Log(λ)
(B) LASSO coefficient path
2 27 35 39 41
lambda.min
lambda.1se
Coefficients
−10 −5 0 5
2 4 6 8 10
−Log(λ)
(C) Random Forest OOB error
Final OOB = 25.0%
OOB error rate
0.20 0.25 0.30 0.35
0 200 400 600 800 1000
Number of trees
(D) RF mtry tuning (repeated 5−fold CV)
Cross−validated AUC
0.60 0.70 0.80 0.90
4 6 8 10 12
mtry
