# Supplementary material for: Systems pharmacology reveals type I interferon and myeloid-like B cell reprogramming as candidate druggable axes in antiphospholipid syndrome
Source: PLoS One. 2026 Aug 26;21(8):e0349155. doi: 10.1371/journal.pone.0349155 (PMC13514046; doi:10.1371/journal.pone.0349155)
Supplement: Supplementary file 18 [file pone.0349155.s036.docx]

# Response to Reviewers

**Manuscript:** PONE-D-26-20131 **Title:** Systems Pharmacology Reveals Type I Interferon and Myeloid-Like B Cell Reprogramming as Druggable Axes in Antiphospholipid Syndrome **Journal:** PLOS ONE

We thank the Academic Editor and both Reviewers for their careful and constructive assessment. We are encouraged that both Reviewers found the study technically sound, the conclusions supported by the data, the data fully available, and the manuscript clearly written. We have addressed every point below. Changes are marked in the “Revised Manuscript with Track Changes” file; page/section locations refer to that file. Reviewer text is quoted in *italics*; our response follows.

Throughout, we have taken particular care to (i) correct the clinical framing of chloroquine, prednisone, and APS epidemiology; (ii) substantially expand the machine-learning methodology and its interpretation; and (iii) present all *in silico* results as explicitly hypothesis-generating.

## Academic Editor

*“…particular attention to the clinical accuracy of the Introduction, clarification and justification of the analytical methods, interpretation of the machine-learning results, cautious presentation of the in silico findings, and overall organization of the manuscript.”*

We have addressed each of these five areas: the Introduction epidemiology and treatment statements are corrected (Reviewer 2, points 1–2); the LASSO/Random Forest methodology is expanded with cross-validation, penalty selection, and overfitting control, and a new methodological figure (S16 Fig) is added (Reviewer 2, points 3–5); Random Forest results are now interpreted within the LASSO framework in the Discussion (Reviewer 2, point 6); drug-repositioning and docking findings are explicitly framed as purely computational and hypothesis-generating (Reviewer 2, point 7); and result statements have been removed from the Discussion (Reviewer 1, organization comment).

## Journal Requirements

1. **Style/formatting.** The manuscript has been formatted to PLOS ONE style, and Supporting Information callouts have been converted to PLOS convention (**S1 Fig … S16 Fig**, **S1 Table … S18 Table**) throughout the text and captions.
2. **Code sharing.** All author-generated analysis code is publicly available without restriction. The Data Availability statement has been updated to: *“All analysis code supporting the findings of this study is publicly available without restriction on GitHub (https://github.com/kukulele201/APS-multiomics-analysis) and archived at Zenodo (DOI: 10.5281/zenodo.21234537).”*
3. **Supporting Information captions.** Captions for all Supporting Information files (S1–S16 Figs; S1–S18 Tables) are provided at the end of the manuscript, and all in-text citations have been updated to match PLOS naming.
4. **Ethics statement.** A full ethics statement has been added to the Materials and Methods section: this study analyzed exclusively publicly available, fully de-identified human transcriptomic datasets from GEO; no new human or animal data were generated; under institutional policy such secondary analysis did not require additional ethics committee approval or informed consent; and the original contributing studies obtained their respective approvals and consent. The end-of-manuscript Ethics Statement declaration was harmonized accordingly.
5. **Recommended citations.** No specific prior works were mandated for citation.

## Reviewer #1

*“…why chloroquine is regarded as an APS first-line therapy? Oral vitamin K antagonists are the gold standard treatment for antiphospholipid syndrome. Hydroxychloroquine, rather than chloroquine, is suggested as an adjunctive therapy to anticoagulation.”*

We fully agree and thank the Reviewer for this important correction. We had incorrectly labeled chloroquine a “first-line APS therapy.” The clinical facts are that vitamin K antagonists remain the standard of care and that **hydroxychloroquine** — not chloroquine — is recommended as an *adjunct* to anticoagulation. We have removed every instance of “first-line APS therapy.” The Connectivity Map compound library contains “chloroquine,” a 4-aminoquinoline antimalarial that is the close pharmacological class-mate of hydroxychloroquine (shared mechanism of action). We now describe the CMap hit accordingly and reframe its role from “validation” to biological plausibility support, e.g.:

- **Abstract:** “…chloroquine — a 4-aminoquinoline antimalarial closely related to hydroxychloroquine, which is recommended as adjunctive therapy in APS — among top ME2 candidates … supporting the biological relevance of the screen.”
- **Results (CMap):** chloroquine reframed as related to hydroxychloroquine (the guideline-recommended adjunct), “supporting the biological relevance of the CMap screen.”
- **Discussion, Conclusions, Figure 4 legend:** parallel edits; “validates”/“first-line” removed.

The Introduction now also states explicitly that anticoagulation is standard and that hydroxychloroquine/low-dose aspirin are adjuncts in selected patients (see Reviewer 2, point 2).

*“…why prednisone was regarded as APS-relevant drug? Steroid use in APS is mostly restricted to the most severe cases, like catastrophic APS (CAPS).”*

Agreed. Prednisone was included among the docked compounds as a representative broadly-immunosuppressive agent that is used in **severe or catastrophic APS (CAPS)**, not as a routine therapy. We have clarified this in Methods: *“…prednisone (a corticosteroid reserved for severe or catastrophic APS), hydroxychloroquine and low-dose aspirin (adjuncts to anticoagulation), and fondaparinux (a parenteral anticoagulant)…”* We also emphasize (Reviewer 2, point 7) that the docking results are purely structural, hypothesis-generating predictions and do not imply therapeutic use of prednisone in routine APS.

*“The results presentation should be limited to Results section and Figures and avoided in the Discussion section.”*

We have removed restated numerical results from the Discussion (quadrant percentages, connectivity scores, docking energies, and specific p-values), retaining interpretation only. The corresponding numbers remain in the Results section and figures. Affected passages: the Drug Repositioning, fondaparinux–IRF7, and Patient-Stratification subsections of the Discussion.

## Reviewer #2 (Chiara Marcon)

*“…the reported prevalence of APS should be revised (it is not 1–5% of the general population), or the statement should be rephrased accordingly.”*

Corrected. The 1–5% figure refers to the population prevalence of **antiphospholipid antibodies**, not of the syndrome. The Introduction now reads: *“Antiphospholipid antibodies are detectable in approximately 1–5% of the general population, whereas APS itself is considerably rarer, with an estimated prevalence of approximately 40–50 per 100,000.”*

*“The claim that anticoagulation represents the only therapeutic option should be moderated to avoid an overly absolute formulation.”*

Moderated. The Introduction now states that *“APS is managed largely with anticoagulation, complemented by hydroxychloroquine and low-dose aspirin in selected patients; however, these strategies do not directly target the underlying pathogenic mechanisms.”* The Abstract wording was likewise softened (“lacks disease-modifying targeted therapies”).

*“…the application of LASSO in such a small cohort requires more detailed information regarding cross-validation strategy, λ selection, and feature selection procedures.”*

We have substantially expanded the Machine Learning subsection of Methods. Briefly: the 77 ME10/ME2 core genes present on both platforms were used as candidate features; features were z-score standardized on the training set and the same parameters applied to the validation set (to prevent information leakage); LASSO logistic regression (glmnet; binomial; α = 1) was fitted with **five-fold internal cross-validation**; the penalty maximizing cross-validated AUC (**λ.min**) was selected; and genes with non-zero coefficients at λ.min defined the sparse three-gene signature. These procedures, including the cross-validation curve and coefficient shrinkage path, are now shown in **S16 Fig (A–B)**.

*“The use of Random Forest should also be better justified, together with a clearer description of how overfitting was controlled, and the corresponding methodological figure should be included in this section.”* / *“…Random Forest outcomes are reported but insufficiently described in the Methods, particularly regarding the feature set used, hyperparameter tuning strategy, and potential overfitting risk given the limited sample size.”*

The Methods now describe Random Forest as a non-linear comparator trained on the same 77-gene feature set (randomForest; 1,000 trees; mtry = √p; importance by mean decrease in accuracy). Overfitting was controlled by (i) reporting out-of-bag error, (ii) **repeated five-fold cross-validation (10 repeats) with mtry tuning over {3, 5, 8, 12}** in the training cohort, and (iii) external validation in an independent tissue. The new **S16 Fig** presents the methodological workflow and diagnostics: LASSO cross-validation (A), coefficient path (B), Random Forest out-of-bag error versus number of trees (C), and mtry tuning by repeated cross-validation (D). Importantly, the internal cross-validated Random Forest AUC (0.76 ± 0.14) is markedly lower than its apparent training/validation AUC, directly illustrating the optimism that motivates our cautious interpretation below.

*“…the absence of any interpretation of the Random Forest results is a limitation; if included, these should be integrated within the LASSO framework and interpreted in light of sample size constraints and overfitting risk.”*

The Discussion now integrates the two models: *“…the higher Random Forest performance is most consistent with optimistic bias and overfitting — indeed its cross-validated AUC within the training cohort was substantially lower than its apparent value — so the sparse, interpretable LASSO model is preferred as the candidate diagnostic signature, with Random Forest serving primarily to corroborate feature importance. All diagnostic estimates require prospective validation in larger, clinically annotated cohorts.”*

*“…drug repositioning and molecular docking results should be presented with greater caution, explicitly emphasizing their purely in silico and hypothesis-generating nature.”*

We have added explicit caveats in both Results and Discussion. In Results (docking): scores are now “purely computational, hypothesis-generating structural plausibility evidence rather than confirmation of target engagement or therapeutic activity.” In the Discussion: “…all repositioning candidates are computational, hypothesis-generating predictions requiring experimental and clinical validation before any therapeutic inference.”

*“…the fondaparinux–IRF7 docking requires stronger biological justification, as the proposed functional interpretation is not immediately supported by mechanistic plausibility.”*

We agree the original framing overstated the mechanism. We have substantially rewritten this passage. We now (i) note that fondaparinux is a synthetic pentasaccharide acting indirectly through antithrombin, so a direct intracellular interaction with IRF7 is *not* established by docking; (ii) explicitly label the observation as speculative and hypothesis-generating; and (iii) provide the limited available biological anchoring (IRF7 as the master IFN-I regulator; documented pleiotropic immunomodulatory effects of heparinoids; recent epigenetic evidence in APS monocytes) while stating that dedicated experimental testing is required before any mechanistic or therapeutic claim can be made. The specific docking energy was removed from the Discussion per Reviewer 1’s organization comment.

## Additional corrections (internal consistency)

During revision we performed a full internal-consistency audit and corrected the following, none of which alter any analytical result:

1. **Whole-blood cohort composition.** The manuscript stated “53 APS, 35 controls,” whereas the underlying phenotype data and all downstream analyses (WGCNA, patient stratification, machine learning) used **60 APS and 28 controls** (total n = 88, unchanged). All affected statements (Methods, Results, and figure legends) are now 60/28.
2. **Docking target count.** The Methods and Results stated “82 module targets,” whereas the docking dataset and the Figure 6 legend contain **81 module targets** (54 ME10 + 27 ME2). Corrected to 81 throughout.
3. **Figure 4 and Figure 5 legends.** The submitted legends for these two figures were out of date relative to the final assembled panels (e.g., an outdated “n = 104” drug count and panel lettering that did not match the in-text callouts). Both legends have been replaced with descriptions that accurately match the current panels, including the correct count of **14 FDA-approved drugs** (Figure 4D) and panels A–E (Figure 4) / A–F (Figure 5) consistent with all in-text figure citations.

Figure 1 and Figure 3 legends. As with Figures 4 and 5, the submitted legends for Figures 1 and 3 described earlier panel versions and did not correspond to the final assembled panels. Both legends have been rewritten to match the current panels and all in-text callouts exactly (Figure 1A–F: study design, WGCNA module–trait heatmap, ME10 GO enrichment, ME2 submodule SM1 GO enrichment, core-gene selection, and core-gene expression; Figure 3A–E: pseudotime UMAP, key gene trends along pseudotime, transitional B-cell DEG volcano, doublet-exclusion validation, and SCENIC regulon activity).

Figure 6 docking value. The NT5E–hydroxychloroquine binding energy is now reported consistently as −9.1 kcal/mol in both the text and the Figure 6 legend, matching the value shown in the Figure 6A affinity heatmap and in Supporting Information Table S18.

4. **Diagnostic-model reporting.** The Figure 5 legend now reports training, validation, and cross-validated AUCs together (LASSO training 0.857 / validation 0.802; Random Forest training 1.000 / validation 0.926 / repeated-CV 0.757 ± 0.141), making the Random Forest overfitting explicit and consistent with the Discussion.

We believe these revisions fully address the Reviewers’ and Editor’s concerns and hope the manuscript is now suitable for publication in PLOS ONE. We thank the Reviewers again for improving the clarity and rigor of this work.

Sincerely, The Authors
