# Supplementary material for: Systems pharmacology reveals type I interferon and myeloid-like B cell reprogramming as candidate druggable axes in antiphospholipid syndrome
Source: PLoS One. 2026 Aug 26;21(8):e0349155. doi: 10.1371/journal.pone.0349155 (PMC13514046; doi:10.1371/journal.pone.0349155)
Supplement: Supplementary file 19 [file pone.0349155.s037.docx]

**Response to Reviewers — Round 2**

**Manuscript: PONE-D-26-20131R1**

**Title: Systems Pharmacology Reveals Type I Interferon and Myeloid-Like B Cell Reprogramming as Candidate Druggable Axes in Antiphospholipid Syndrome**

We thank the Academic Editor and both reviewers for their careful re-evaluation and for recognizing that the manuscript has been substantially improved. We are grateful to Reviewer #1 for recommending acceptance. We have addressed every remaining point raised by Reviewer #3 and by the Academic Editor. As all comments concerned the interpretation, presentation, and limitations of the computational analyses, we focused this revision on textual reframing, clearer acknowledgement of limitations, and more cautious interpretation rather than on adding new experimental analyses. All changes are marked in the "Revised Manuscript with Track Changes" file. Reviewer comments are reproduced below in italics, followed by our response and the specific wording introduced.

**RESPONSE TO THE ACADEMIC EDITOR**

The Editor asked us to ensure that (i) the conclusions accurately reflect the computational nature of the study, (ii) the methodological limitations are clearly acknowledged, and (iii) associations and predictions are not presented as experimentally established causal or clinically actionable findings.

We have implemented this guidance throughout the manuscript. Strong claim verbs ("identifies druggable targets," "foundation for precision medicine," "actionable candidates") have been replaced with hypothesis-generating language ("prioritizes candidate targets," "generates hypotheses"). Molecular docking is now consistently framed as a structural-plausibility assessment rather than a validation strategy. The four-quadrant stratification is described as a computational classification that requires prospective clinical validation before any clinical use. The proposed regulatory role of SPI1 is explicitly qualified as association rather than established causation. The specific edits are itemized in the reviewer responses below.

**RESPONSE TO REVIEWER #1**

"I would like to thank the Authors for their comprehensive response in addressing all my questions. I recommend it for publication."

We sincerely thank Reviewer #1 for the positive assessment and recommendation for publication. No further changes were requested.

**RESPONSE TO REVIEWER #3**

We thank Reviewer #3 for the constructive and detailed evaluation and for acknowledging that the revised manuscript is "clearer and scientifically stronger" and that "the computational workflow is comprehensive and technically sound." We address each of the six remaining points below.

**Comment 1.** *"The conclusions remain stronger than the presented evidence… Expressions such as 'identifies druggable targets', 'providing a foundation for pathway-guided precision medicine', and 'revealing actionable drug repositioning candidates' suggest a level of biological validation that is not provided… I recommend consistently replacing these statements with more cautious wording such as 'prioritizes candidate targets', 'suggests potential therapeutic candidates', 'generates hypotheses for future investigation', 'identifies candidate molecular pathways.'"*

**Response:** We agree and have systematically moderated these statements throughout the manuscript, adopting the reviewer's suggested vocabulary.

- Abstract: "…to identify druggable targets in APS" → "…to prioritize candidate therapeutic targets in APS"; and the closing sentence "This framework nominates druggable targets… providing a foundation for pathway-guided precision medicine in APS" → "This framework prioritizes candidate targets across both IFN-I and degranulation pathways, generating hypotheses for pathway-guided therapeutic development that require experimental and clinical validation."

- Introduction: "delivering actionable drug repositioning candidates validated across independent datasets" → "generating drug-repositioning hypotheses supported by concordance across independent datasets"; and "Our approach identifies druggable targets… revealing diverse therapeutic opportunities" → "Our approach prioritizes candidate targets… suggesting diverse therapeutic opportunities — requiring experimental validation —".

- Discussion (opening): "molecular docking to nominate druggable targets… which our dual-module approach directly addresses" → "molecular docking to nominate candidate targets… which our dual-module approach seeks to inform".

- Conclusion: "This integrative systems pharmacology study identifies druggable targets…" → "…prioritizes candidate therapeutic targets…".

- Consistency sweep: to apply this vocabulary uniformly, we also softened the title ("…as Druggable Axes" → "…as Candidate Druggable Axes"), the Introduction ("integrates multi-omics data to identify druggable targets" → "…to prioritize candidate therapeutic targets"), the Results subheading ("Network-Based Drug Repositioning Identifies Therapeutic Candidates" → "…Prioritizes Candidate Therapeutics"), the target-prioritization text ("ranked druggable targets" / "demonstrated druggability" → "prioritized candidate targets" / "database-annotated druggability"), and the Abstract ("External validation confirmed ME2 pathway modulation…" → "External datasets supported ME2 pathway modulation…", since these are secondary analyses of public data).

**Comment 2.** *"Biological significance of molecular docking remains limited… Docking scores alone do not demonstrate biological interaction, target engagement or therapeutic efficacy. No molecular dynamics simulations, binding affinity calculations, biochemical validation or functional assays are provided. Consequently, the docking results should be presented primarily as supportive structural observations rather than an independent validation strategy… discuss this limitation more explicitly in the Discussion."*

**Response:** We agree and have reframed molecular docking as a supportive structural observation throughout.

- Section and figure framing: the Results subheading "Molecular Docking Validation" is now "Molecular Docking: Structural Plausibility Assessment"; the Discussion subheading "Drug Repositioning and Validation" is now "Drug Repositioning and Structural Plausibility"; and "molecular docking validation" in the Discussion is now "molecular docking for structural plausibility assessment". (The Figure 6 title was already revised in the previous round to "Molecular Docking Assesses Structural Plausibility of Drug-Target Interactions.")

- Introduction aim (v): "validate predicted drug-target interactions through molecular docking" → "assess the structural plausibility of predicted drug-target interactions through molecular docking".

- Discussion limitation (ii) has been expanded to state explicitly: "molecular docking provides supportive structural observations only and does not demonstrate physical binding, target engagement, or therapeutic efficacy — no molecular dynamics simulations, binding free-energy calculations, or functional assays were performed, and although the expression-matched negative-control panel showed no module-specific selectivity, the docking results should be regarded as supportive structural observations rather than an independent validation strategy."

- We also corrected the Figure 1 legend, which still described "molecular docking validation"; it now reads "molecular docking for structural plausibility assessment," consistent with the rest of the manuscript.

**Comment 3.** *"Machine learning validation remains limited… the external validation cohort consists of only 18 samples and originates from a different biological source (whole blood for training versus neutrophils for validation)… it cannot be considered equivalent to validation in an independent clinical cohort generated using the same biological material. This limitation deserves stronger emphasis in the Discussion."*

**Response:** We agree and have added an explicit statement to the Discussion: "Importantly, external validation relied on only 18 neutrophil samples drawn from a different biological source than the whole-blood training data; such cross-tissue validation demonstrates signal portability but cannot substitute for validation in an independent clinical cohort profiled from the same biological material. All diagnostic estimates should therefore be regarded as preliminary and require prospective validation in larger, clinically annotated cohorts." This complements the existing acknowledgment (previous round) that the higher apparent Random Forest AUC reflects optimistic bias/overfitting given the small validation sample. We additionally adjusted the Methods wording from "overfitting was controlled by" to "overfitting was mitigated and assessed by," since a small external cohort cannot fully control overfitting.

**Comment 4.** *"WGCNA performed on a very small discovery cohort… Why was WGCNA performed using the smaller neutrophil dataset rather than the substantially larger whole-blood cohort? A brief explanation of this design choice would improve the manuscript."*

**Response:** We thank the reviewer for this helpful question and have added the rationale to both the Methods and Results. Module discovery was intentionally performed in the purified-neutrophil cohort rather than in whole blood because co-expression networks derived from whole blood are strongly confounded by inter-individual variation in leukocyte composition, whereas a homogeneous, purified cell population yields modules that better reflect cell-intrinsic co-regulation; the choice is also biologically motivated, given the central role of neutrophil effector programs (NETosis and degranulation) in APS pathogenesis. The larger whole-blood cohort (n = 88) was correspondingly reserved as an independent cohort for module-preservation testing, which is where its greater sample size is most valuable. We have added this explanation to the Methods (WGCNA subsection) and clarified the Results sentence to read: "we performed module discovery by WGCNA in the purified neutrophil cohort (n = 18) and then validated module preservation in the larger whole blood cohort (n = 88)." The Abstract and the Figure 1 legend were likewise clarified to indicate that the neutrophil cohort was used for module discovery and whole blood for module-preservation testing (the Figure 1 legend previously stated that both cohorts "underwent WGCNA for module identification").

**Comment 5.** *"Clinical interpretation of patient stratification… the four molecular quadrants are currently defined solely by transcriptomic signatures. No association with thrombosis, pregnancy morbidity, antibody profile, disease activity, treatment response, or other clinical parameters is presented. Therefore, the proposed stratification should be described as a computational classification requiring prospective clinical validation rather than a clinically actionable framework."*

**Response:** We agree and have reframed the stratification accordingly. In the Results, "A pathway-guided treatment framework maps each quadrant to targeted therapies" now reads "This quadrant scheme is a hypothetical, computationally derived framework mapping each molecular subtype to candidate targeted therapies," and we now state explicitly: "Because the quadrants are defined solely by transcriptomic signatures and were not linked to thrombosis, pregnancy morbidity, antibody profile, disease activity, or treatment response (clinical annotations were unavailable in the public datasets), this stratification represents a computational classification requiring prospective clinical validation before any clinical application." The corresponding Discussion limitation and the Conclusion were revised to the same effect, and the Figure 5E legend "Treatment decision tree" is now "Hypothesized, pathway-guided decision tree… (requiring prospective clinical validation)." For consistency with this reframing, the Figure 5 title and the corresponding Results section heading were changed from "Clinical Translation…" to "Computational Patient Stratification and Candidate Pathway Mapping."

**Comment 6.** *"Interpretation of SPI1… the presented evidence consists of correlation analysis, SCENIC regulon inference, and in silico perturbation. These analyses support association but do not establish causality. The manuscript should consistently distinguish between correlation and regulation throughout the Results and Discussion."*

**Response:** We agree and have sharpened this distinction. In the Results, "This is consistent with SPI1 playing a regulatory role in the myeloid-like transcriptional program" now reads "This association is consistent with, but does not by itself establish, a regulatory role for SPI1…," and "a key SPI1-dependent target" is now "a candidate SPI1-associated target." In the Discussion, the sentence summarizing the SPI1 evidence now closes: "…these analyses are correlative and support association rather than experimentally established causal regulation." These additions reinforce the caveat already present in the Results (previous round), which noted that establishing causality would require experimental SPI1 knockdown in primary B cells.

We hope these revisions address the remaining concerns and bring the framing of our predictive and mechanistic statements into line with the computational nature of the evidence. We thank the Editor and reviewers again for their time and constructive input.
