# Supplementary material for: Systems pharmacology reveals type I interferon and myeloid-like B cell reprogramming as candidate druggable axes in antiphospholipid syndrome
Source: PLoS One. 2026 Aug 26;21(8):e0349155. doi: 10.1371/journal.pone.0349155 (PMC13514046; doi:10.1371/journal.pone.0349155)
Supplement: Peer review history

# Peer review: Decision Letter 0

**DOI:** 10.1371/journal.pone.0349155.r001

**Authors:** Tomasz Kaminski

6 Jul 2026

Dear Dr. Wang,

Thank you for submitting your manuscript to PLOS ONE. After careful consideration, we feel that it has merit but does not fully meet PLOS ONE’s publication criteria as it currently stands. Therefore, we invite you to submit a revised version of the manuscript that addresses the points raised during the review process.

Please submit your revised manuscript by Aug 20 2026 11:59PM. If you will need more time than this to complete your revisions, please reply to this message or contact the journal office at plosone@plos.org. When you're ready to submit your revision, log on to https://www.editorialmanager.com/pone/ and select the 'Submissions Needing Revision' folder to locate your manuscript file.

- A letter that responds to each point raised by the academic editor and reviewer(s). You should upload this letter as a separate file labeled 'Response to Reviewers'.
- A marked-up copy of your manuscript that highlights changes made to the original version. You should upload this as a separate file labeled 'Revised Manuscript with Track Changes'.
- An unmarked version of your revised paper without tracked changes. You should upload this as a separate file labeled 'Manuscript'.

If you would like to make changes to your financial disclosure, please include your updated statement in your cover letter. Guidelines for resubmitting your figure files are available below the reviewer comments at the end of this letter.

If applicable, we recommend that you deposit your laboratory protocols in protocols.io to enhance the reproducibility of your results. Protocols.io assigns your protocol its own identifier (DOI) so that it can be cited independently in the future. For instructions see: https://journals.plos.org/plosone/s/submission-guidelines#loc-laboratory-protocols. Additionally, PLOS ONE offers an option for publishing peer-reviewed Lab Protocol articles, which describe protocols hosted on protocols.io. Read more information on sharing protocols at https://plos.org/protocols?utm_medium=editorial-email&utm_source=authorletters&utm_campaign=protocols.

As the corresponding author, your ORCID iD is verified in the submission system and will appear in the published article. PLOS supports the use of ORCID, and we encourage all coauthors to register for an ORCID iD and use it as well. Please encourage your coauthors to verify their ORCID iD within the submission system before final acceptance, as unverified ORCID iDs will not appear in the published article. *Only*  the individual author can complete the verification step; PLOS staff *cannot*  verify ORCID iDs on behalf of authors.

We look forward to receiving your revised manuscript.

Kind regards,

Tomasz W. Kaminski

Academic Editor

PLOS One

Journal Requirements:

When submitting your revision, we need you to address these additional requirements.

1. Please ensure that your manuscript meets PLOS ONE's style requirements, including those for file naming. The PLOS ONE style templates can be found at

https://journals.plos.org/plosone/s/file?id=wjVg/PLOSOne_formatting_sample_main_body.pdf and

https://journals.plos.org/plosone/s/file?id=ba62/PLOSOne_formatting_sample_title_authors_affiliations.pdf

2. Please note that PLOS One has specific guidelines on code sharing for submissions in which author-generated code underpins the findings in the manuscript. In these cases, we expect all author-generated code to be made available without restrictions upon publication of the work. Please review our guidelines at https://journals.plos.org/plosone/s/materials-and-software-sharing#loc-sharing-code and ensure that your code is shared in a way that follows best practice and facilitates reproducibility and reuse.

3. Please include captions for your Supporting Information files at the end of your manuscript, and update any in-text citations to match accordingly. Please see our Supporting Information guidelines for more information: http://journals.plos.org/plosone/s/supporting-information.

4. Please include your full ethics statement in the ‘Methods’ section of your manuscript file. In your statement, please include the full name of the IRB or ethics committee who approved or waived your study, as well as whether or not you obtained informed written or verbal consent. If consent was waived for your study, please include this information in your statement as well.

5. If the reviewer comments include a recommendation to cite specific previously published works, please review and evaluate these publications to determine whether they are relevant and should be cited. There is no requirement to cite these works unless the editor has indicated otherwise.

Additional Editor Comments:

Dear Authors,

Thank you for submitting your manuscript. The study addresses an important and clinically relevant topic, and the search for new therapeutic options in antiphospholipid syndrome is of clear interest.

However, based on the reviewers’ comments and editorial assessment, the manuscript requires major revision before it can be further considered. Please carefully address all reviewer comments, with particular attention to the clinical accuracy of the Introduction, clarification and justification of the analytical methods, interpretation of the machine-learning results, cautious presentation of the in silico findings, and overall organization of the manuscript.

Please provide a detailed point-by-point response to the reviewers and revise the manuscript accordingly.

Best regards,

Tomasz W Kaminski

Academic Editor

[Note: HTML markup is below. Please do not edit.]

Reviewers' comments:

Reviewer's Responses to Questions

**Comments to the Author**

1. Is the manuscript technically sound, and do the data support the conclusions?

Reviewer #1: Yes

Reviewer #2: Yes

**********

2. Has the statistical analysis been performed appropriately and rigorously?

Reviewer #1: I Don't Know

Reviewer #2: Yes

**********

3. Have the authors made all data underlying the findings in their manuscript fully available?

The PLOS Data policy

Reviewer #1: Yes

Reviewer #2: Yes

**********

4. Is the manuscript presented in an intelligible fashion and written in standard English?

Reviewer #1: Yes

Reviewer #2: Yes

**********

Reviewer #1: The topic of the study addresses a very important issue concerning the search for new therapeutic options in antiphospholipid syndrome. This is particularly relevant, since, as the authors themselves have noted, the available treatments are only symptomatic. However, some clarifications are needed before the work could be properly evaluated. Moreover the manuscript requires some reorganization. Therefore, I suggest some major revisions to be performed by the Authors.

Firstly, why chloroquine is regarded as an APS first-line therapy? Oral vitamin K antagonists are the gold standard treatment for antiphospholipid syndrome. Hydroxychloroquine, rather than chloroquine, is suggested as an adjunctive therapy to anticoagulation.

Secondly, why prednisone was regarded as APS-relevant drug? Steroid use in APS is mostly restricted to the most severe cases, like catastrophic APS (CAPS).

Minor comments regarding the organization of the manuscript:

The results presentation should be limited to Results section and Figures and avoided in the Discussion section.

Reviewer #2: In the Introduction, the reported prevalence of APS should be revised (it is not 1–5% of the general population), or the statement should be rephrased accordingly. The claim that anticoagulation represents the only therapeutic option should be moderated to avoid an overly absolute formulation. In the Methods, the section is overall appropriate; however, the application of LASSO in such a small cohort requires more detailed information regarding cross-validation strategy, λ selection, and feature selection procedures. The use of Random Forest should also be better justified, together with a clearer description of how overfitting was controlled, and the corresponding methodological figure should be included in this section. In the Results, Random Forest outcomes are reported but insufficiently described in the Methods, particularly regarding the feature set used, hyperparameter tuning strategy, and potential overfitting risk given the limited sample size. In the Discussion, the absence of any interpretation of the Random Forest results is a limitation; if included, these should be integrated within the LASSO framework and interpreted in light of sample size constraints and overfitting risk. Furthermore, drug repositioning and molecular docking results should be presented with greater caution, explicitly emphasizing their purely in silico and hypothesis-generating nature. Finally, the fondaparinux–IRF7 docking requires stronger biological justification, as the proposed functional interpretation is not immediately supported by mechanistic plausibility.

**********

what does this mean?). If published, this will include your full peer review and any attached files.

If you choose “no”, your identity will remain anonymous but your review may still be made public.

**Do you want your identity to be public for this peer review?**  For information about this choice, including consent withdrawal, please see our Privacy Policy

Reviewer #1: No

Reviewer #2: **Yes:** Chiara Marcon

**********

[NOTE: If reviewer comments were submitted as an attachment file, they will be attached to this email and accessible via the submission site. Please log into your account, locate the manuscript record, and check for the action link "View Attachments". If this link does not appear, there are no attachment files.]

To ensure your figures meet our technical requirements, please review our figure guidelines: https://journals.plos.org/plosone/s/figures

You may also use PLOS’s free figure tool, NAAS, to help you prepare publication quality figures: https://journals.plos.org/plosone/s/figures#loc-tools-for-figure-preparation.

NAAS will assess whether your figures meet our technical requirements by comparing each figure against our figure specifications.

# Author comment: Author response to Decision Letter 1

**DOI:** 10.1371/journal.pone.0349155.r002

12 Jul 2026

Response to Reviewers

Manuscript: PONE-D-26-20131 Title: Systems Pharmacology Reveals Type I Interferon and Myeloid-Like B Cell Reprogramming as Druggable Axes in Antiphospholipid Syndrome Journal: PLOS ONE

We thank the Academic Editor and both Reviewers for their careful and constructive assessment. We are encouraged that both Reviewers found the study technically sound, the conclusions supported by the data, the data fully available, and the manuscript clearly written. We have addressed every point below. Changes are marked in the “Revised Manuscript with Track Changes” file; page/section locations refer to that file. Reviewer text is quoted in italics; our response follows.

Throughout, we have taken particular care to (i) correct the clinical framing of chloroquine, prednisone, and APS epidemiology; (ii) substantially expand the machine-learning methodology and its interpretation; and (iii) present all in silico results as explicitly hypothesis-generating.

________________________________________

Academic Editor

“…particular attention to the clinical accuracy of the Introduction, clarification and justification of the analytical methods, interpretation of the machine-learning results, cautious presentation of the in silico findings, and overall organization of the manuscript.”

We have addressed each of these five areas: the Introduction epidemiology and treatment statements are corrected (Reviewer 2, points 1–2); the LASSO/Random Forest methodology is expanded with cross-validation, penalty selection, and overfitting control, and a new methodological figure (S16 Fig) is added (Reviewer 2, points 3–5); Random Forest results are now interpreted within the LASSO framework in the Discussion (Reviewer 2, point 6); drug-repositioning and docking findings are explicitly framed as purely computational and hypothesis-generating (Reviewer 2, point 7); and result statements have been removed from the Discussion (Reviewer 1, organization comment).

Journal Requirements

1. Style/formatting. The manuscript has been formatted to PLOS ONE style, and Supporting Information callouts have been converted to PLOS convention (S1 Fig … S16 Fig, S1 Table … S18 Table) throughout the text and captions.

2. Code sharing. All author-generated analysis code is publicly available without restriction. The Data Availability statement has been updated to: “All analysis code supporting the findings of this study is publicly available without restriction on GitHub (https://github.com/kukulele201/APS-multiomics-analysis) and archived at Zenodo (DOI: 10.5281/zenodo.21234537).”

3. Supporting Information captions. Captions for all Supporting Information files (S1–S16 Figs; S1–S18 Tables) are provided at the end of the manuscript, and all in-text citations have been updated to match PLOS naming.

4. Ethics statement. A full ethics statement has been added to the Materials and Methods section: this study analyzed exclusively publicly available, fully de-identified human transcriptomic datasets from GEO; no new human or animal data were generated; under institutional policy such secondary analysis did not require additional ethics committee approval or informed consent; and the original contributing studies obtained their respective approvals and consent. The end-of-manuscript Ethics Statement declaration was harmonized accordingly.

5. Recommended citations. No specific prior works were mandated for citation.

________________________________________

Reviewer #1

“…why chloroquine is regarded as an APS first-line therapy? Oral vitamin K antagonists are the gold standard treatment for antiphospholipid syndrome. Hydroxychloroquine, rather than chloroquine, is suggested as an adjunctive therapy to anticoagulation.”

We fully agree and thank the Reviewer for this important correction. We had incorrectly labeled chloroquine a “first-line APS therapy.” The clinical facts are that vitamin K antagonists remain the standard of care and that hydroxychloroquine — not chloroquine — is recommended as an adjunct to anticoagulation. We have removed every instance of “first-line APS therapy.” The Connectivity Map compound library contains “chloroquine,” a 4-aminoquinoline antimalarial that is the close pharmacological class-mate of hydroxychloroquine (shared mechanism of action). We now describe the CMap hit accordingly and reframe its role from “validation” to biological plausibility support, e.g.:

• Abstract: “…chloroquine — a 4-aminoquinoline antimalarial closely related to hydroxychloroquine, which is recommended as adjunctive therapy in APS — among top ME2 candidates … supporting the biological relevance of the screen.”

• Results (CMap): chloroquine reframed as related to hydroxychloroquine (the guideline-recommended adjunct), “supporting the biological relevance of the CMap screen.”

• Discussion, Conclusions, Figure 4 legend: parallel edits; “validates”/“first-line” removed.

The Introduction now also states explicitly that anticoagulation is standard and that hydroxychloroquine/low-dose aspirin are adjuncts in selected patients (see Reviewer 2, point 2).

“…why prednisone was regarded as APS-relevant drug? Steroid use in APS is mostly restricted to the most severe cases, like catastrophic APS (CAPS).”

Agreed. Prednisone was included among the docked compounds as a representative broadly-immunosuppressive agent that is used in severe or catastrophic APS (CAPS), not as a routine therapy. We have clarified this in Methods: “…prednisone (a corticosteroid reserved for severe or catastrophic APS), hydroxychloroquine and low-dose aspirin (adjuncts to anticoagulation), and fondaparinux (a parenteral anticoagulant)…” We also emphasize (Reviewer 2, point 7) that the docking results are purely structural, hypothesis-generating predictions and do not imply therapeutic use of prednisone in routine APS.

“The results presentation should be limited to Results section and Figures and avoided in the Discussion section.”

We have removed restated numerical results from the Discussion (quadrant percentages, connectivity scores, docking energies, and specific p-values), retaining interpretation only. The corresponding numbers remain in the Results section and figures. Affected passages: the Drug Repositioning, fondaparinux–IRF7, and Patient-Stratification subsections of the Discussion.

________________________________________

Reviewer #2 (Chiara Marcon)

“…the reported prevalence of APS should be revised (it is not 1–5% of the general population), or the statement should be rephrased accordingly.”

Corrected. The 1–5% figure refers to the population prevalence of antiphospholipid antibodies, not of the syndrome. The Introduction now reads: “Antiphospholipid antibodies are detectable in approximately 1–5% of the general population, whereas APS itself is considerably rarer, with an estimated prevalence of approximately 40–50 per 100,000.”

“The claim that anticoagulation represents the only therapeutic option should be moderated to avoid an overly absolute formulation.”

Moderated. The Introduction now states that “APS is managed largely with anticoagulation, complemented by hydroxychloroquine and low-dose aspirin in selected patients; however, these strategies do not directly target the underlying pathogenic mechanisms.” The Abstract wording was likewise softened (“lacks disease-modifying targeted therapies”).

“…the application of LASSO in such a small cohort requires more detailed information regarding cross-validation strategy, λ selection, and feature selection procedures.”

We have substantially expanded the Machine Learning subsection of Methods. Briefly: the 77 ME10/ME2 core genes present on both platforms were used as candidate features; features were z-score standardized on the training set and the same parameters applied to the validation set (to prevent information leakage); LASSO logistic regression (glmnet; binomial; α = 1) was fitted with five-fold internal cross-validation; the penalty maximizing cross-validated AUC (λ.min) was selected; and genes with non-zero coefficients at λ.min defined the sparse three-gene signature. These procedures, including the cross-validation curve and coefficient shrinkage path, are now shown in S16 Fig (A–B).

“The use of Random Forest should also be better justified, together with a clearer description of how overfitting was controlled, and the corresponding methodological figure should be included in this section.” / “…Random Forest outcomes are reported but insufficiently described in the Methods, particularly regarding the feature set used, hyperparameter tuning strategy, and potential overfitting risk given the limited sample size.”

The Methods now describe Random Forest as a non-linear comparator trained on the same 77-gene feature set (randomForest; 1,000 trees; mtry = √p; importance by mean decrease in accuracy). Overfitting was controlled by (i) reporting out-of-bag error, (ii) repeated five-fold cross-validation (10 repeats) with mtry tuning over {3, 5, 8, 12} in the training cohort, and (iii) external validation in an independent tissue. The new S16 Fig presents the methodological workflow and diagnostics: LASSO cross-validation (A), coefficient path (B), Random Forest out-of-bag error versus number of trees (C), and mtry tuning by repeated cross-validation (D). Importantly, the internal cross-validated Random Forest AUC (0.76 ± 0.14) is markedly lower than its apparent training/validation AUC, directly illustrating the optimism that motivates our cautious interpretation below.

“…the absence of any interpretation of the Random Forest results is a limitation; if included, these should be integrated within the LASSO framework and interpreted in light of sample size constraints and overfitting risk.”

The Discussion now integrates the two models: “…the higher Random Forest performance is most consistent with optimistic bias and overfitting — indeed its cross-validated AUC within the training cohort was substantially lower than its apparent value — so the sparse, interpretable LASSO model is preferred as the candidate diagnostic signature, with Random Forest serving primarily to corroborate feature importance. All diagnostic estimates require prospective validation in larger, clinically annotated cohorts.”

“…drug repositioning and molecular docking results should be presented with greater caution, explicitly emphasizing their purely in silico and hypothesis-generating nature.”

We have added explicit caveats in both Results and Discussion. In Results (docking): scores are now “purely computational, hypothesis-generating structural plausibility evidence rather than confirmation of target engagement or therapeutic activity.” In the Discussion: “…all repositioning candidates are computational, hypothesis-generating predictions requiring experimental and clinical validation before any therapeutic inference.”

“…the fondaparinux–IRF7 docking requires stronger biological justification, as the proposed functional interpretation is not immediately supported by mechanistic plausibility.”

We agree the original framing overstated the mechanism. We have substantially rewritten this passage. We now (i) note that fondaparinux is a synthetic pentasaccharide acting indirectly through antithrombin, so a direct intracellular interaction with IRF7 is not established by docking; (ii) explicitly label the observation as speculative and hypothesis-generating; and (iii) provide the limited available biological anchoring (IRF7 as the master IFN-I regulator; documented pleiotropic immunomodulatory effects of heparinoids; recent epigenetic evidence in APS monocytes) while stating that dedicated experimental testing is required before any mechanistic or therapeutic claim can be made. The specific docking energy was removed from the Discussion per Reviewer 1’s organization comment.

________________________________________

Additional corrections (internal consistency)

During revision we performed a full internal-consistency audit and corrected the following, none of which alter any analytical result:

1. Whole-blood cohort composition. The manuscript stated “53 APS, 35 controls,” whereas the underlying phenotype data and all downstream analyses (WGCNA, patient stratification, machine learning) used 60 APS and 28 controls (total n = 88, unchanged). All affected statements (Methods, Results, and figure legends) are now 60/28.

2. Docking target count. The Methods and Results stated “82 module targets,” whereas the docking dataset and the Figure 6 legend contain 81 module targets (54 ME10 + 27 ME2). Corrected to 81 throughout.

3. Figure 4 and Figure 5 legends. The submitted legends for these two figures were out of date relative to the final assembled panels (e.g., an outdated “n = 104” drug count and panel lettering that did not match the in-text callouts). Both legends have been replaced with descriptions that accurately match the current panels, including the correct count of 14 FDA-approved drugs (Figure 4D) and panels A–E (Figure 4) / A–F (Figure 5) consistent with all in-text figure citations.

Figure 1 and Figure 3 legends. As with Figures 4 and 5, the submitted legends for Figures 1 and 3 described earlier panel versions and did not correspond to the final assembled panels. Both legends have been rewritten to match the current panels and all in-text callouts exactly (Figure 1A–F: study design, WGCNA module–trait heatmap, ME10 GO enrichment, ME2 submodule SM1 GO enrichment, core-gene selection, and core-gene expression; Figure 3A–E: pseudotime UMAP, key gene trends along pseudotime, transitional B-cell DEG volcano, doublet-exclusion validation, and SCENIC regulon activity).

Figure 6 docking value. The NT5E–hydroxychloroquine binding energy is now reported consistently as −9.1 kcal/mol in both the text and the Figure 6 legend, matching the value shown in the Figure 6A affinity heatmap and in Supporting Information Table S18.

4. Diagnostic-model reporting. The Figure 5 legend now reports training, validation, and cross-validated AUCs together (LASSO training 0.857 / validation 0.802; Random Forest training 1.000 / validation 0.926 / repeated-CV 0.757 ± 0.141), making the Random Forest overfitting explicit and consistent with the Discussion.

________________________________________

We believe these revisions fully address the Reviewers’ and Editor’s concerns and hope the manuscript is now suitable for publication in PLOS ONE. We thank the Reviewers again for improving the clarity and rigor of this work.

Sincerely, The Authors

Attachment Submitted filename: 03_Response_to_Reviewers.docx

# Peer review: Decision Letter 1

**DOI:** 10.1371/journal.pone.0349155.r003

**Authors:** Tomasz Kaminski

29 Jul 2026

Dear Dr. Wang,

Thank you for submitting your manuscript to PLOS ONE. After careful consideration, we feel that it has merit but does not fully meet PLOS ONE’s publication criteria as it currently stands. Therefore, we invite you to submit a revised version of the manuscript that addresses the points raised during the review process.

Please submit your revised manuscript by Sep 12 2026 11:59PM. If you will need more time than this to complete your revisions, please reply to this message or contact the journal office at plosone@plos.org. When you're ready to submit your revision, log on to https://www.editorialmanager.com/pone/ and select the 'Submissions Needing Revision' folder to locate your manuscript file.

- A letter that responds to each point raised by the academic editor and reviewer(s). You should upload this letter as a separate file labeled 'Response to Reviewers'.
- A marked-up copy of your manuscript that highlights changes made to the original version. You should upload this as a separate file labeled 'Revised Manuscript with Track Changes'.
- An unmarked version of your revised paper without tracked changes. You should upload this as a separate file labeled 'Manuscript'.

If you would like to make changes to your financial disclosure, please include your updated statement in your cover letter. Guidelines for resubmitting your figure files are available below the reviewer comments at the end of this letter.

If applicable, we recommend that you deposit your laboratory protocols in protocols.io to enhance the reproducibility of your results. Protocols.io assigns your protocol its own identifier (DOI) so that it can be cited independently in the future. For instructions see: https://journals.plos.org/plosone/s/submission-guidelines#loc-laboratory-protocols. Additionally, PLOS ONE offers an option for publishing peer-reviewed Lab Protocol articles, which describe protocols hosted on protocols.io. Read more information on sharing protocols at https://plos.org/protocols?utm_medium=editorial-email&utm_source=authorletters&utm_campaign=protocols.

As the corresponding author, your ORCID iD is verified in the submission system and will appear in the published article. PLOS supports the use of ORCID, and we encourage all coauthors to register for an ORCID iD and use it as well. Please encourage your coauthors to verify their ORCID iD within the submission system before final acceptance, as unverified ORCID iDs will not appear in the published article. *Only*  the individual author can complete the verification step; PLOS staff *cannot*  verify ORCID iDs on behalf of authors.

We look forward to receiving your revised manuscript.

Kind regards,

Tomasz W. Kaminski

Academic Editor

PLOS One

Journal Requirements:

If the reviewer comments include a recommendation to cite specific previously published works, please review and evaluate these publications to determine whether they are relevant and should be cited. There is no requirement to cite these works unless the editor has indicated otherwise.

Please review your reference list to ensure that it is complete and correct. If you have cited papers that have been retracted, please include the rationale for doing so in the manuscript text, or remove these references and replace them with relevant current references. Any changes to the reference list should be mentioned in the rebuttal letter that accompanies your revised manuscript. If you need to cite a retracted article, indicate the article’s retracted status in the References list and also include a citation and full reference for the retraction notice.

Additional Editor Comments:

Dear Authors,

Thank you for submitting the revised version of your manuscript. The reviewers recognize that the manuscript has been substantially improved, and one reviewer now recommends acceptance.

However, the second reviewer has identified several remaining concerns regarding the interpretation, presentation, and limitations of the computational analyses. These comments do not require additional experimental work, but they should be addressed carefully before the manuscript can be considered for publication.

I am therefore inviting a minor revision. Please provide a detailed point-by-point response to all remaining reviewer comments and revise the manuscript accordingly. In particular, please ensure that the conclusions accurately reflect the computational nature of the study, that the methodological limitations are clearly acknowledged, and that associations and predictions are not presented as experimentally established causal or clinically actionable findings.

Please clearly indicate all changes made in the revised manuscript.

Sincerely,

Tomasz W Kaminski

[Note: HTML markup is below. Please do not edit.]

Reviewers' comments:

Reviewer's Responses to Questions

**Comments to the Author**

Reviewer #1: All comments have been addressed

Reviewer #3: (No Response)

**********

2. Is the manuscript technically sound, and do the data support the conclusions?

Reviewer #1: Yes

Reviewer #3: Partly

**********

3. Has the statistical analysis been performed appropriately and rigorously?

Reviewer #1: Yes

Reviewer #3: No

**********

4. Have the authors made all data underlying the findings in their manuscript fully available?

The PLOS Data policy

Reviewer #1: Yes

Reviewer #3: Yes

**********

5. Is the manuscript presented in an intelligible fashion and written in standard English?

Reviewer #1: Yes

Reviewer #3: Yes

**********

Reviewer #1: I would like to thank the Authors for their comprehensive response in addressing all my questions. I recommend it for publication.

Reviewer #3: The authors have substantially revised the manuscript and have addressed the majority of the concerns raised during the previous review round. In particular, the clinical background has been corrected, the machine learning methodology has been considerably expanded, the discussion of drug repositioning and molecular docking has become more cautious, and several internal inconsistencies have been resolved. Overall, the revised manuscript is clearer and scientifically stronger than the original submission.

The study presents an interesting integrative bioinformatics framework combining WGCNA, single-cell RNA sequencing, Connectivity Map analysis, DrugBank annotation, molecular docking and machine learning to identify potential therapeutic targets in antiphospholipid syndrome (APS). The computational workflow is comprehensive and technically sound.

Nevertheless, several important issues remain that should be addressed before publication.

1. The conclusions remain stronger than the presented evidence

Although the authors have moderated several statements, the manuscript still occasionally overstates the implications of purely computational findings.

Expressions such as

• "identifies druggable targets",

• "providing a foundation for pathway-guided precision medicine",

• "revealing actionable drug repositioning candidates"

suggest a level of biological validation that is not provided in this study.

The entire workflow is based on publicly available transcriptomic datasets combined with computational prediction methods. No experimental validation of the identified pathways, biomarkers or therapeutic targets is presented.

I recommend consistently replacing these statements with more cautious wording such as:

• "prioritizes candidate targets",

• "suggests potential therapeutic candidates",

• "generates hypotheses for future investigation",

• "identifies candidate molecular pathways."

This change would better reflect the actual level of evidence.

2. Biological significance of molecular docking remains limited

The discussion has been improved, but I remain unconvinced that the molecular docking analysis substantially strengthens the manuscript.

Docking scores alone do not demonstrate biological interaction, target engagement or therapeutic efficacy. No molecular dynamics simulations, binding affinity calculations, biochemical validation or functional assays are provided.

Consequently, the docking results should be presented primarily as supportive structural observations rather than an independent validation strategy.

The authors may also consider discussing this limitation more explicitly in the Discussion section.

3. Machine learning validation remains limited

The revised machine learning section is considerably improved.

However, the external validation cohort consists of only 18 samples and originates from a different biological source (whole blood for training versus neutrophils for validation).

While cross-tissue validation is interesting, it cannot be considered equivalent to validation in an independent clinical cohort generated using the same biological material.

This limitation deserves stronger emphasis in the Discussion.

4. WGCNA performed on a very small discovery cohort

The authors appropriately acknowledge that the WGCNA discovery dataset consists of only 18 samples.

Although module preservation analysis partially addresses this concern, an important methodological question remains:

Why was WGCNA performed using the smaller neutrophil dataset rather than the substantially larger whole-blood cohort?

A brief explanation of this design choice would improve the manuscript.

5. Clinical interpretation of patient stratification

The proposed ME10/ME2 stratification is interesting.

However, the four molecular quadrants are currently defined solely by transcriptomic signatures.

No association with

• thrombosis,

• pregnancy morbidity,

• antibody profile,

• disease activity,

• treatment response,

or other clinical parameters is presented.

Therefore, the proposed stratification should be described as a computational classification requiring prospective clinical validation rather than a clinically actionable framework.

6. Interpretation of SPI1

The manuscript repeatedly suggests that SPI1 regulates the observed myeloid-like transcriptional program.

However, the presented evidence consists of:

• correlation analysis,

• SCENIC regulon inference,

• in silico perturbation.

These analyses support association but do not establish causality.

The manuscript should consistently distinguish between correlation and regulation throughout the Results and Discussion.

**********

what does this mean?). If published, this will include your full peer review and any attached files.

If you choose “no”, your identity will remain anonymous but your review may still be made public.

**Do you want your identity to be public for this peer review?**  For information about this choice, including consent withdrawal, please see our Privacy Policy

Reviewer #1: No

Reviewer #3: No

**********

[NOTE: If reviewer comments were submitted as an attachment file, they will be attached to this email and accessible via the submission site. Please log into your account, locate the manuscript record, and check for the action link "View Attachments". If this link does not appear, there are no attachment files.]

To ensure your figures meet our technical requirements, please review our figure guidelines: https://journals.plos.org/plosone/s/figures

You may also use PLOS’s free figure tool, NAAS, to help you prepare publication quality figures: https://journals.plos.org/plosone/s/figures#loc-tools-for-figure-preparation.

NAAS will assess whether your figures meet our technical requirements by comparing each figure against our figure specifications.

# Author comment: Author response to Decision Letter 2

**DOI:** 10.1371/journal.pone.0349155.r004

1 Aug 2026

Response to Reviewers — Round 2

Manuscript: PONE-D-26-20131R1

Title: Systems Pharmacology Reveals Type I Interferon and Myeloid-Like B Cell Reprogramming as Candidate Druggable Axes in Antiphospholipid Syndrome

We thank the Academic Editor and both reviewers for their careful re-evaluation and for recognizing that the manuscript has been substantially improved. We are grateful to Reviewer #1 for recommending acceptance. We have addressed every remaining point raised by Reviewer #3 and by the Academic Editor. As all comments concerned the interpretation, presentation, and limitations of the computational analyses, we focused this revision on textual reframing, clearer acknowledgement of limitations, and more cautious interpretation rather than on adding new experimental analyses. All changes are marked in the "Revised Manuscript with Track Changes" file. Reviewer comments are reproduced below in italics, followed by our response and the specific wording introduced.

RESPONSE TO THE ACADEMIC EDITOR

The Editor asked us to ensure that (i) the conclusions accurately reflect the computational nature of the study, (ii) the methodological limitations are clearly acknowledged, and (iii) associations and predictions are not presented as experimentally established causal or clinically actionable findings.

We have implemented this guidance throughout the manuscript. Strong claim verbs ("identifies druggable targets," "foundation for precision medicine," "actionable candidates") have been replaced with hypothesis-generating language ("prioritizes candidate targets," "generates hypotheses"). Molecular docking is now consistently framed as a structural-plausibility assessment rather than a validation strategy. The four-quadrant stratification is described as a computational classification that requires prospective clinical validation before any clinical use. The proposed regulatory role of SPI1 is explicitly qualified as association rather than established causation. The specific edits are itemized in the reviewer responses below.

RESPONSE TO REVIEWER #1

"I would like to thank the Authors for their comprehensive response in addressing all my questions. I recommend it for publication."

We sincerely thank Reviewer #1 for the positive assessment and recommendation for publication. No further changes were requested.

RESPONSE TO REVIEWER #3

We thank Reviewer #3 for the constructive and detailed evaluation and for acknowledging that the revised manuscript is "clearer and scientifically stronger" and that "the computational workflow is comprehensive and technically sound." We address each of the six remaining points below.

Comment 1. "The conclusions remain stronger than the presented evidence… Expressions such as 'identifies druggable targets', 'providing a foundation for pathway-guided precision medicine', and 'revealing actionable drug repositioning candidates' suggest a level of biological validation that is not provided… I recommend consistently replacing these statements with more cautious wording such as 'prioritizes candidate targets', 'suggests potential therapeutic candidates', 'generates hypotheses for future investigation', 'identifies candidate molecular pathways.'"

Response: We agree and have systematically moderated these statements throughout the manuscript, adopting the reviewer's suggested vocabulary.

- Abstract: "…to identify druggable targets in APS" → "…to prioritize candidate therapeutic targets in APS"; and the closing sentence "This framework nominates druggable targets… providing a foundation for pathway-guided precision medicine in APS" → "This framework prioritizes candidate targets across both IFN-I and degranulation pathways, generating hypotheses for pathway-guided therapeutic development that require experimental and clinical validation."

- Introduction: "delivering actionable drug repositioning candidates validated across independent datasets" → "generating drug-repositioning hypotheses supported by concordance across independent datasets"; and "Our approach identifies druggable targets… revealing diverse therapeutic opportunities" → "Our approach prioritizes candidate targets… suggesting diverse therapeutic opportunities — requiring experimental validation —".

- Discussion (opening): "molecular docking to nominate druggable targets… which our dual-module approach directly addresses" → "molecular docking to nominate candidate targets… which our dual-module approach seeks to inform".

- Conclusion: "This integrative systems pharmacology study identifies druggable targets…" → "…prioritizes candidate therapeutic targets…".

- Consistency sweep: to apply this vocabulary uniformly, we also softened the title ("…as Druggable Axes" → "…as Candidate Druggable Axes"), the Introduction ("integrates multi-omics data to identify druggable targets" → "…to prioritize candidate therapeutic targets"), the Results subheading ("Network-Based Drug Repositioning Identifies Therapeutic Candidates" → "…Prioritizes Candidate Therapeutics"), the target-prioritization text ("ranked druggable targets" / "demonstrated druggability" → "prioritized candidate targets" / "database-annotated druggability"), and the Abstract ("External validation confirmed ME2 pathway modulation…" → "External datasets supported ME2 pathway modulation…", since these are secondary analyses of public data).

Comment 2. "Biological significance of molecular docking remains limited… Docking scores alone do not demonstrate biological interaction, target engagement or therapeutic efficacy. No molecular dynamics simulations, binding affinity calculations, biochemical validation or functional assays are provided. Consequently, the docking results should be presented primarily as supportive structural observations rather than an independent validation strategy… discuss this limitation more explicitly in the Discussion."

Response: We agree and have reframed molecular docking as a supportive structural observation throughout.

- Section and figure framing: the Results subheading "Molecular Docking Validation" is now "Molecular Docking: Structural Plausibility Assessment"; the Discussion subheading "Drug Repositioning and Validation" is now "Drug Repositioning and Structural Plausibility"; and "molecular docking validation" in the Discussion is now "molecular docking for structural plausibility assessment". (The Figure 6 title was already revised in the previous round to "Molecular Docking Assesses Structural Plausibility of Drug-Target Interactions.")

- Introduction aim (v): "validate predicted drug-target interactions through molecular docking" → "assess the structural plausibility of predicted drug-target interactions through molecular docking".

- Discussion limitation (ii) has been expanded to state explicitly: "molecular docking provides supportive structural observations only and does not demonstrate physical binding, target engagement, or therapeutic efficacy — no molecular dynamics simulations, binding free-energy calculations, or functional assays were performed, and although the expression-matched negative-control panel showed no module-specific selectivity, the docking results should be regarded as supportive structural observations rather than an independent validation strategy."

- We also corrected the Figure 1 legend, which still described "molecular docking validation"; it now reads "molecular docking for structural plausibility assessment," consistent with the rest of the manuscript.

Comment 3. "Machine learning validation remains limited… the external validation cohort consists of only 18 samples and originates from a different biological source (whole blood for training versus neutrophils for validation)… it cannot be considered equivalent to validation in an independent clinical cohort generated using the same biological material. This limitation deserves stronger emphasis in the Discussion."

Response: We agree and have added an explicit statement to the Discussion: "Importantly, external validation relied on only 18 neutrophil samples drawn from a different biological source than the whole-blood training data; such cross-tissue validation demonstrates signal portability but cannot substitute for validation in an independent clinical cohort profiled from the same biological material. All diagnostic estimates should therefore be regarded as preliminary and require prospective validation in larger, clinically annotated cohorts." This complements the existing acknowledgment (previous round) that the higher apparent Random Forest AUC reflects optimistic bias/overfitting given the small validation sample. We additionally adjusted the Methods wording from "overfitting was controlled by" to "overfitting was mitigated and assessed by," since a small external cohort cannot fully control overfitting.

Comment 4. "WGCNA performed on a very small discovery cohort… Why was WGCNA performed using the smaller neutrophil dataset rather than the substantially larger whole-blood cohort? A brief explanation of this design choice would improve the manuscript."

Response: We thank the reviewer for this helpful question and have added the rationale to both the Methods and Results. Module discovery was intentionally performed in the purified-neutrophil cohort rather than in whole blood because co-expression networks derived from whole blood are strongly confounded by inter-individual variation in leukocyte composition, whereas a homogeneous, purified cell population yields modules that better reflect cell-intrinsic co-regulation; the choice is also biologically motivated, given the central role of neutrophil effector programs (NETosis and degranulation) in APS pathogenesis. The larger whole-blood cohort (n = 88) was correspondingly reserved as an independent cohort for module-preservation testing, which is where its greater sample size is most valuable. We have added this explanation to the Methods (WGCNA subsection) and clarified the Results sentence to read: "we performed module discovery by WGCNA in the purified neutrophil cohort (n = 18) and then validated module preservation in the larger whole blood cohort (n = 88)." The Abstract and the Figure 1 legend were likewise clarified to indicate that the neutrophil cohort was used for module discovery and whole blood for module-preservation testing (the Figure 1 legend previously stated that both cohorts "underwent WGCNA for module identification").

Comment 5. "Clinical interpretation of patient stratification… the four molecular quadrants are currently defined solely by transcriptomic signatures. No association with thrombosis, pregnancy morbidity, antibody profile, disease activity, treatment response, or other clinical parameters is presented. Therefore, the proposed stratification should be described as a computational classification requiring prospective clinical validation rather than a clinically actionable framework."

Response: We agree and have reframed the stratification accordingly. In the Results, "A pathway-guided treatment framework maps each quadrant to targeted therapies" now reads "This quadrant scheme is a hypothetical, computationally derived framework mapping each molecular subtype to candidate targeted therapies," and we now state explicitly: "Because the quadrants are defined solely by transcriptomic signatures and were not linked to thrombosis, pregnancy morbidity, antibody profile, disease activity, or treatment response (clinical annotations were unavailable in the public datasets), this stratification represents a computational classification requiring prospective clinical validation before any clinical application." The corresponding Discussion limitation and the Conclusion were revised to the same effect, and the Figure 5E legend "Treatment decision tree" is now "Hypothesized, pathway-guided decision tree… (requiring prospective clinical validation)." For consistency with this reframing, the Figure 5 title and the corresponding Results section heading were changed from "Clinical Translation…" to "Computational Patient Stratification and Candidate Pathway Mapping."

Comment 6. "Interpretation of SPI1… the presented evidence consists of correlation analysis, SCENIC regulon inference, and in silico perturbation. These analyses support association but do not establish causality. The manuscript should consistently distinguish between correlation and regulation throughout the Results and Discussion."

Response: We agree and have sharpened this distinction. In the Results, "This is consistent with SPI1 playing a regulatory role in the myeloid-like transcriptional program" now reads "This association is consistent with, but does not by itself establish, a regulatory role for SPI1…," and "a key SPI1-dependent target" is now "a candidate SPI1-associated target." In the Discussion, the sentence summarizing the SPI1 evidence now closes: "…these analyses are correlative and support association rather than experimentally established causal regulation." These additions reinforce the caveat already present in the Results (previous round), which noted that establishing causality would require experimental SPI1 knockdown in primary B cells.

We hope these revisions address the remaining concerns and bring the framing of our predictive and mechanistic statements into line with the computational nature of the evidence. We thank the Editor and reviewers again for their time and constructive input.

Attachment Submitted filename: 03_Response_to_Reviewers_auresp_2.docx

# Editor report: Decision Letter 2

**DOI:** 10.1371/journal.pone.0349155.r005

**Authors:** Tomasz Kaminski

3 Aug 2026

Systems Pharmacology Reveals Type I Interferon and Myeloid-Like B Cell Reprogramming as Candidate Druggable Axes in Antiphospholipid Syndrome

PONE-D-26-20131R2

Dear Dr. Wang,

We’re pleased to inform you that your manuscript has been judged scientifically suitable for publication and will be formally accepted for publication once it meets all outstanding technical requirements.

Within one week, you’ll receive an e-mail detailing the required amendments. When these have been addressed, you’ll receive a formal acceptance letter and your manuscript will be scheduled for publication.

An invoice will be generated when your article is formally accepted. Please note, if your institution has a publishing partnership with PLOS and your article meets the relevant criteria, all or part of your publication costs will be covered. Please make sure your user information is up-to-date by logging into Editorial Manager at Editorial Manager® and clicking the ‘Update My Information' link at the top of the page. For questions related to billing, please contact billing support.

If your institution or institutions have a press office, please notify them about your upcoming paper to help maximize its impact. If they’ll be preparing press materials, please inform our press team as soon as possible -- no later than 48 hours after receiving the formal acceptance. Your manuscript will remain under strict press embargo until 2 pm Eastern Time on the date of publication. For more information, please contact onepress@plos.org.

Kind regards,

Tomasz W. Kaminski

Academic Editor

PLOS One

Additional Editor Comments:

Dear Authors,

Thank you for your careful revisions. The manuscript has improved substantially, and the reviewers’ main concerns have been adequately addressed. I believe that the manuscript may now be accepted, with a few minor technical corrections to be addressed during the proofreading stage.

a) Verify the Zenodo DOI and replace the current DOI if it is incorrect. Confirm that the final DOI opens the archived version of the analysis code.

b) Reconcile the reported total of 26,936 B cells with the doublet analysis reporting 138 myeloid-like B cells plus 27,748 other B cells. Update the Abstract, Methods, Results, figure legends, and supplementary files consistently.

c) Recheck the numbers reported for GSE205465, GSE252972, and GSE252397 against the samples actually included in the analysis. Identify any excluded samples by accession or sample ID, provide the reason for exclusion, and correct all related Methods, Results, and figure legends.

These are minor reporting issues and do not require substantial additional analysis.

Best regards,

Tomasz W Kaminski

Reviewers' comments:

# Editor report: Acceptance letter

**DOI:** 10.1371/journal.pone.0349155.r006

**Authors:** Tomasz Kaminski

PONE-D-26-20131R2

PLOS One

Dear Dr. Wang,

I'm pleased to inform you that your manuscript has been deemed suitable for publication in PLOS One. Congratulations! Your manuscript is now being handed over to our production team.

At this stage, our production department will prepare your paper for publication. This includes ensuring the following:

* All references, tables, and figures are properly cited

* All relevant supporting information is included in the manuscript submission,

* There are no issues that prevent the paper from being properly typeset

You will receive further instructions from the production team, including instructions on how to review your proof when it is ready. Please keep in mind that we are working through a large volume of accepted articles, so please give us a few days to review your paper and let you know the next and final steps.

Lastly, if your institution or institutions have a press office, please let them know about your upcoming paper now to help maximize its impact. If they'll be preparing press materials, please inform our press team within the next 48 hours. Your manuscript will remain under strict press embargo until 2 pm Eastern Time on the date of publication. For more information, please contact onepress@plos.org.

You will receive an invoice from PLOS for your publication fee after your manuscript has reached the completed accept phase. If you receive an email requesting payment before acceptance or for any other service, this may be a phishing scheme. Learn how to identify phishing emails and protect your accounts at https://explore.plos.org/phishing.

If we can help with anything else, please email us at customercare@plos.org.

Thank you for submitting your work to PLOS One and supporting open access.

Kind regards,

PLOS ONE Editorial Office Staff

on behalf of

Dr. Tomasz W. Kaminski

Academic Editor

PLOS One
